# Design, Synthesis and Biological Evaluation of Rhodanine-Based Hydrazide Derivatives as Antibacterial, Antifungal, and Antitubercular Agents

**DOI:** 10.3390/antibiotics15090853

**Published:** 2026-09-01

**Authors:** Agata Paneth, Izabela Korona-Głowniak, Agnieszka Głogowska, Jacek Szczepański, Dominik Włodarczyk, Katarzyna Suśniak, Ewa Augustynowicz-Kopeć, Nazar Trotsko

**Affiliations:** 1 Department of Organic Chemistry, Medical University of Lublin, Chodzki 4A, 20-093 Lublin, Poland; agata.paneth@umlub.edu.pl (A.P.); jaacek.szczepanski.93@gmail.com (J.S.); 54246@umlub.edu.pl (D.W.); 2 Department of Pharmaceutical Microbiology, Medical University in Lublin, Chodzki 1, 20-093 Lublin, Poland; izabela.korona-glowniak@umlub.edu.pl (I.K.-G.); katarzyna.susniak@umlub.edu.pl (K.S.); 3 Department of Microbiology, National Tuberculosis and Lung Diseases Research Institute, Plocka 26, 01-138 Warsaw, Poland; a.glogowska@igichp.edu.pl (A.G.); e.kopec@igichp.edu.pl (E.A.-K.)

**Keywords:** rhodanine, thiazolidin-4-one, hydrazides, antimicrobial activity, antifungal activity, antitubercular activity

## Abstract

**Background/Objectives**: The rapid emergence of antimicrobial resistance has created an urgent need for new chemotypes with activity against bacterial, fungal, and mycobacterial pathogens. Rhodanine-based compounds are recognized as privileged scaffolds in medicinal chemistry because of their broad spectrum of biological activities, making them attractive candidates for the development of novel antimicrobial agents. **Methods**: A library of thirty-two novel rhodanine-based hydrazide derivatives (**21**–**52**) was synthesized by condensation of 5-ethoxymethylidenerhodanine derivatives with aromatic acid hydrazides and characterized by IR, ^1^H NMR, and ^13^C NMR spectroscopy. Their drug-likeness and pharmacokinetics properties were evaluated using SwissADME, while antimicrobial activity was assessed against Gram-positive and Gram-negative bacteria, *Candida* spp., and both drug-susceptible and drug-resistant *Mycobacterium tuberculosis* strains using minimum inhibitory concentration (MIC) assays. **Results**: The synthesized compounds demonstrated selective activity against Gram-positive bacteria, whereas only limited activity was observed against Gram-negative species. The most potent derivatives (**36**, **40**, **46**, and **50**) inhibited *Micrococcus luteus* with MIC values as low as 31.3 mg/L. Several compounds also demonstrated noteworthy antifungal activity, particularly against *Candida parapsilosis* (MIC = 15.6–31.3 µg/mL). Pyridyl-containing analogues displayed moderate antitubercular activity and, importantly, compounds **49** and **52** retaining activity against both drug-susceptible and drug-resistant *M. tuberculosis* strains. In silico analysis indicated favorable drug-like properties for most compounds, with good compliance with Lipinski’s rule of five and predicted oral bioavailability. Preliminary structure–activity relationship analysis revealed that a 3-phenylrhodanine core combined with *para*-halogen or *para*-nitro substituents significantly enhanced antimicrobial potency. **Conclusions**: The present study identifies rhodanine-based hydrazide derivatives as promising multifunctional antimicrobial scaffolds with activity against Gram-positive bacteria, pathogenic yeasts, and *M. tuberculosis*. The identified structure–activity relationships provide a rational basis for further lead optimization and support continued development of this chemotype as a potential source of new antimicrobial agents.

## 1. Introduction

Antimicrobial resistance (AMR) is recognized as one of the greatest global threats to public health, substantially reducing the effectiveness of available antimicrobial therapies and contributing to increased morbidity, mortality, and healthcare costs [1,2,3]. The rapid emergence and worldwide dissemination of multidrug-resistant (MDR) pathogens, including methicillin-resistant *Staphylococcus aureus* (MRSA), vancomycin-resistant *Enterococcus* species (VRE), carbapenem-resistant *Enterobacterales* (CRE), and multidrug-resistant strains of *Pseudomonas aeruginosa* and *Acinetobacter baumannii*, have severely limited therapeutic options and highlighted the urgent need for new antimicrobial agents capable of overcoming existing resistance mechanisms [4,5,6,7].

To address this challenge, current antimicrobial drug discovery focuses on the identification of new chemotypes, the optimization of known pharmacophores, and the design of hybrid molecules that combine complementary biological properties within a single scaffold [8,9]. Such strategies aim not only to improve antimicrobial potency but also to reduce the likelihood of resistance development and broaden the spectrum of activity [2,8,9].

Among privileged heterocyclic scaffolds, rhodanine (2-thioxothiazolidin-4-one) has attracted considerable attention owing to its synthetic accessibility, structural versatility, and broad range of biological activities. Rhodanine derivatives have demonstrated antimicrobial, antiviral, anticancer, antidiabetic, anti-inflammatory, and neuroprotective properties, while the scaffold readily accommodates structural modifications that facilitate systematic structure–activity relationship (SAR) studies and medicinal chemistry optimization [10,11,12].

Particularly promising are rhodanine-based hybrids, which combine the rhodanine core with additional pharmacophores to enhance biological activity and improve physicochemical properties. Numerous derivatives have demonstrated promising anticancer activities through interactions with kinases, apoptosis-related proteins, and other molecular targets involved in tumor progression [12,13]. In addition, rhodanine-based compounds have shown considerable promise as antidiabetic agents, particularly as inhibitors of protein tyrosine phosphatase 1B (PTP1B), α-glucosidase, and other enzymes relevant to glucose metabolism [14]. Antiviral and antimicrobial applications remain another active research area, with numerous derivatives exhibiting activity against multidrug-resistant bacterial strains, fungi, and viral pathogens [15,16].

**Figure 1:**
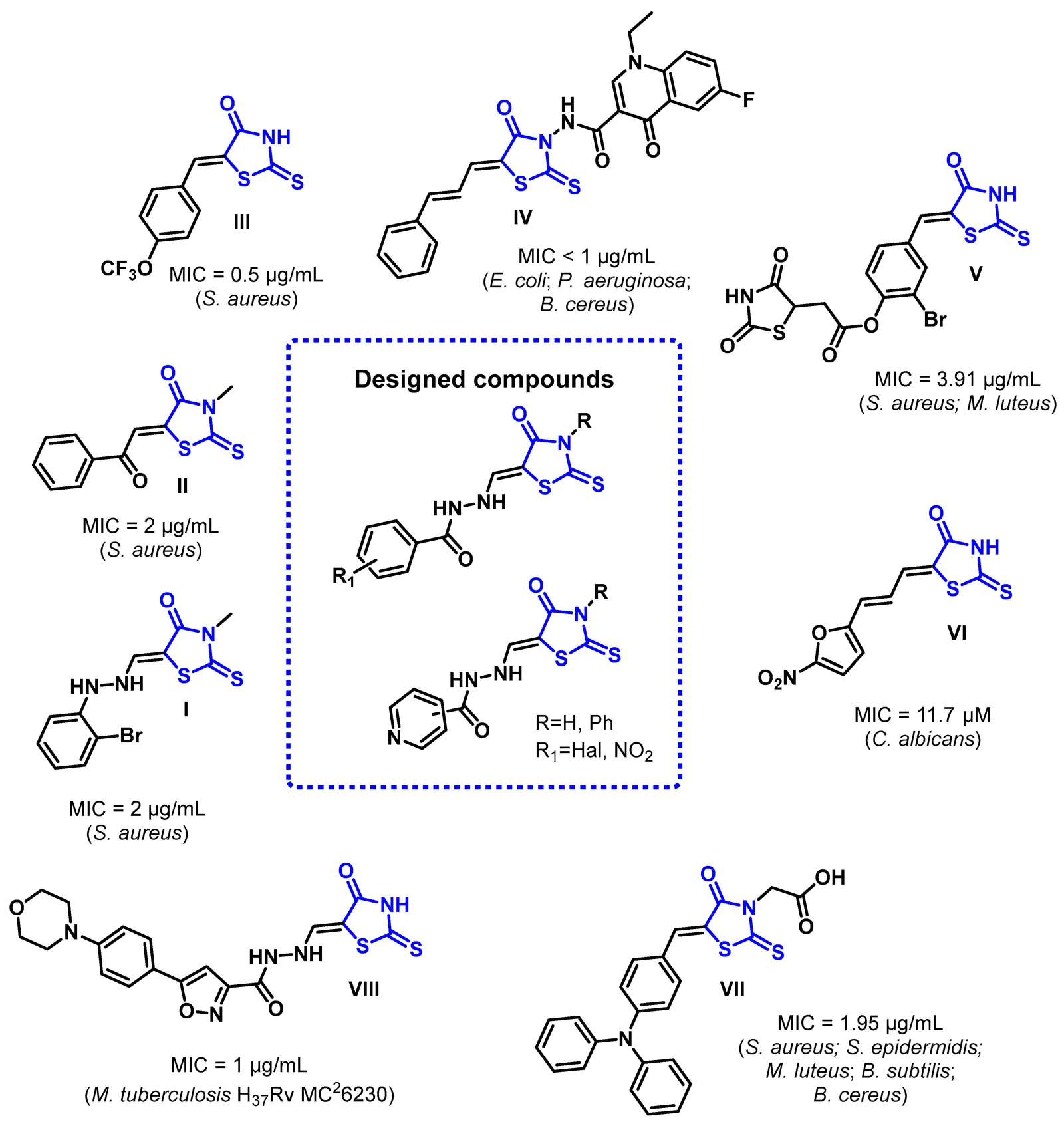
Literature reported rhodanine derivatives with antibacterial, antifungal, and antimycobacterial activity and rationale for the designing of rhodanine-hydrazide hybrids.

In terms of antimicrobial activity, numerous rhodanine derivatives have demonstrated potent antimicrobial activity, particularly against Gram-positive bacteria, fungal pathogens, and *Mycobacterium tuberculosis*. For example, 3-unsubstituted rhodanine and 3-methylrhodanine derivatives (compounds **I**–**III**) exhibited strong activity against *S. aureus* (MIC 0.5–2 µg/mL), whereas modification at the C-5 position (compounds **V** and **VII**) expanded antibacterial potency against additional Gram-positive pathogens [17,18,19]. Hybridization of the rhodanine scaffold with other pharmacophores has proven particularly successful. A rhodanine-quinolone hybrid (**IV**) displayed excellent activity against both Gram-positive and Gram-negative bacteria, including *Escherichia coli* and *Pseudomonas aeruginosa* (MIC < 1 µg/mL) [20], while 5-hetarylidene rhodanines (**VI**) demonstrated significant antifungal activity against *Candida albicans* (MIC = 11.7 µM) [21]. Likewise, rhodanine-hydrazide conjugates have emerged as promising antimycobacterial agents, with selected derivatives exhibiting potent activity against *M. tuberculosis* (**VIII**, MIC = 1 µg/mL) [22] (Figure 1).

Despite these encouraging findings, the antimicrobial potential of rhodanine-hydrazide hybrids remains insufficiently explored. Available studies are limited in scope, typically focusing on individual microbial groups or small numbers of compounds, and comprehensive evaluations encompassing antibacterial, antifungal, and antimycobacterial activities within a single, structurally related series are scarce. Furthermore, systematic analyses correlating structural modifications with antimicrobial activity and predicted pharmacokinetic properties remain limited, restricting the rational optimization of this promising chemotype.

Therefore, the aim of the present study was to synthesize a new series of rhodanine-hydrazide hybrids and comprehensively evaluate their antibacterial, antifungal, and antimycobacterial activities. In addition, in silico ADME analysis was performed to assess their drug-likeness and pharmacokinetic potential, while structure–activity relationship (SAR) analysis was conducted to identify the structural features associated with enhanced antimicrobial activity. Collectively, these findings provide new insights into the biological potential of rhodanine-hydrazide hybrids and establish a foundation for their further optimization as lead structures for combating drug-resistant pathogens.

## 2. Results

### 2.1. Chemical Synthesis

**Scheme 1:**
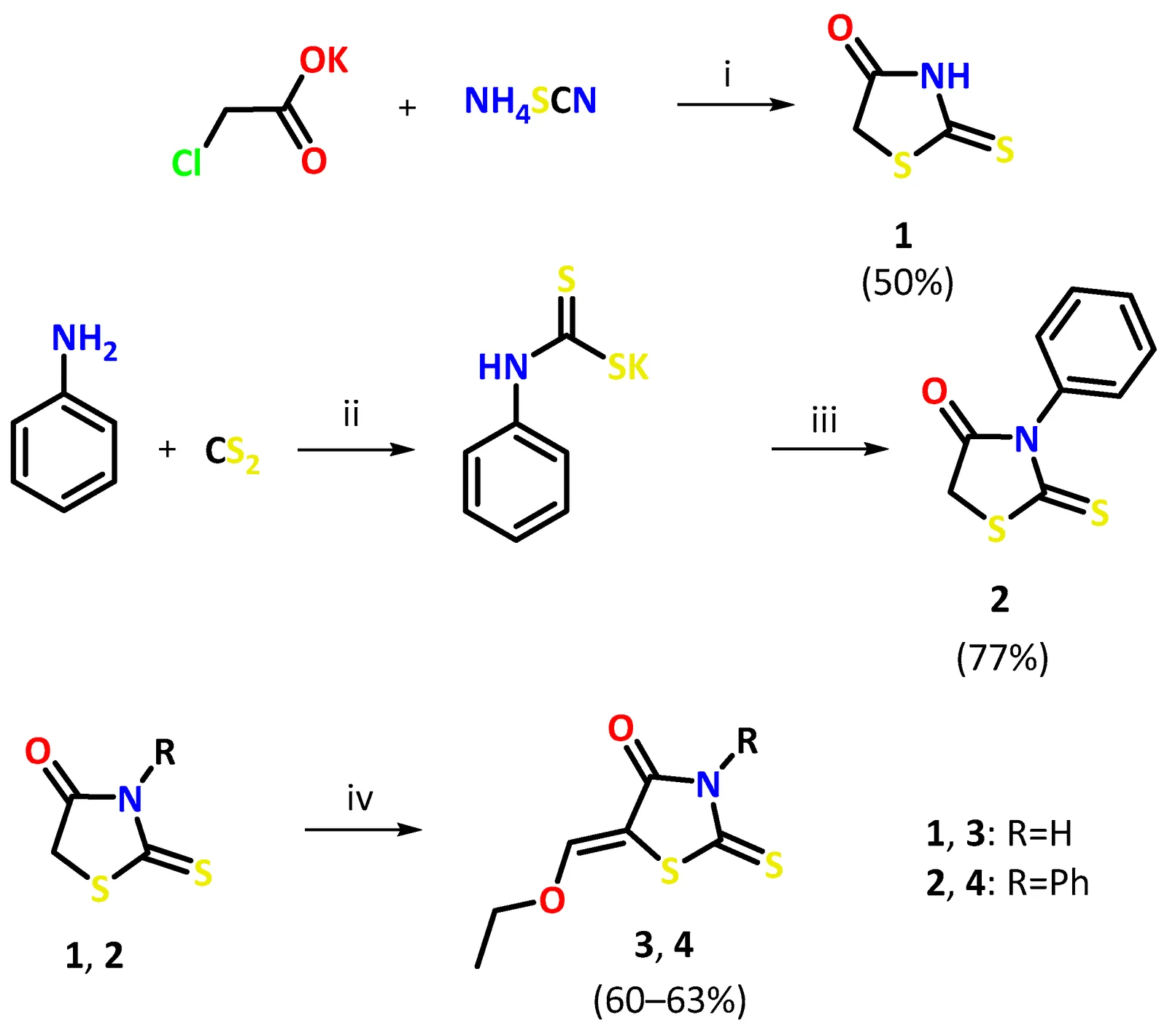
Synthesis of rhodanine (**1**), 3-phenylrhodanine (**2**) and 5-ethoxymethylidenerhodanines (**3**, **4**). Reagents and conditions: (i) HCl, heated 90 °C, 5 min; (ii) KOH; (iii) ClCH_2_COOK; HCl, heated 90 °C, 5 min; (iv) CH(OEt)_3_, Ac_2_O, heated under reflux, 3 h.

**Scheme 2:**
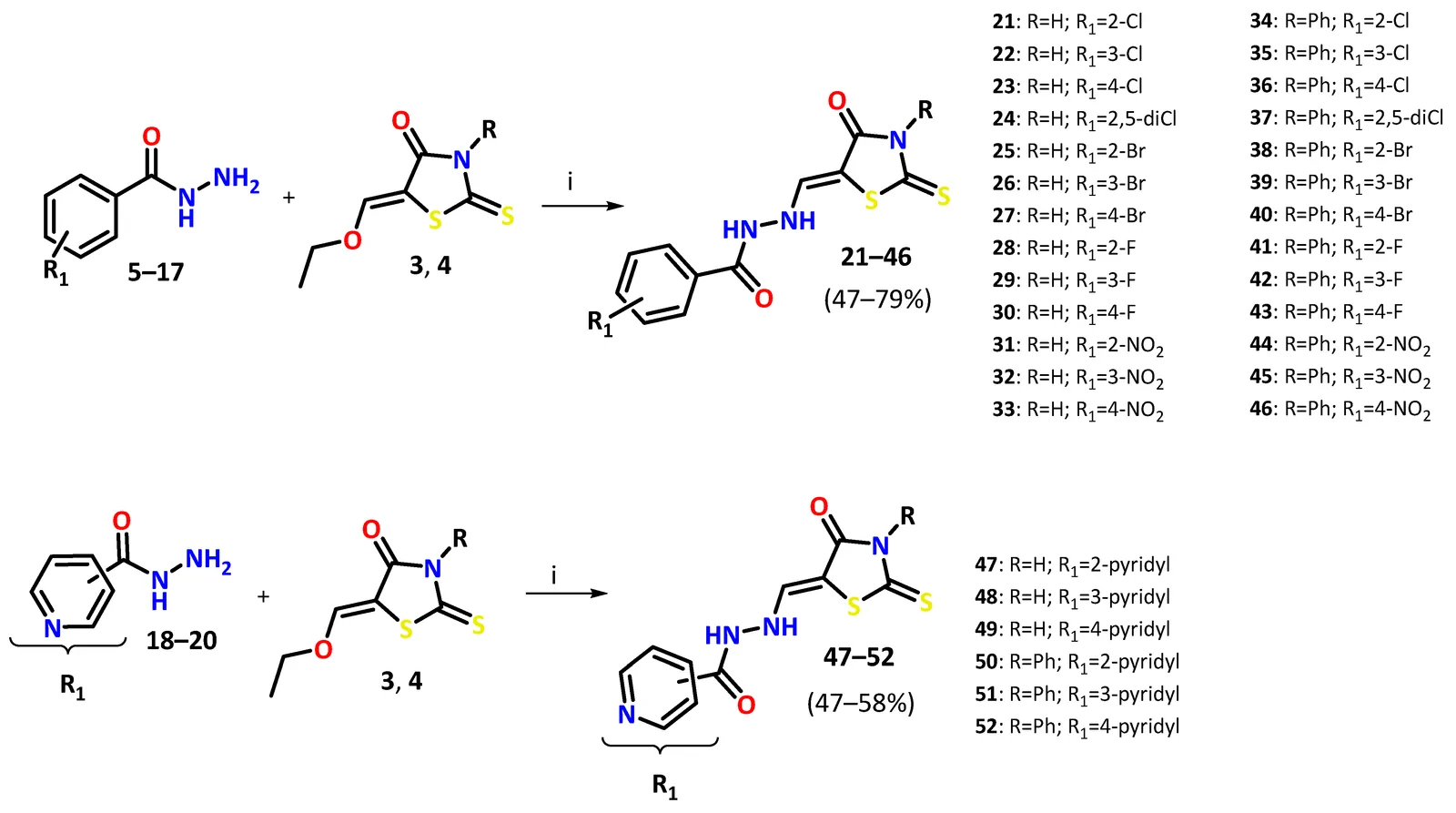
Synthesis of rhodanine-hydrazide hybrids (**21**–**52**). Reagents and conditions: (i) anhydrous ethanol, heated under reflux, 30 min–3 h.

The starting compounds used in this study were rhodanine (**1**) and 3-phenylrhodanine (**2**). Rhodanine **1** was synthesized from the potassium salt of chloroacetic acid and ammonium thiocyanate according to a previously reported procedure [23]. 3-Phenylrhodanine **2** was prepared by reacting aniline with carbon disulfide under basic conditions, followed by the addition of chloroacetic acid neutralized with potassium carbonate. The resulting reaction mixture was treated with hydrochloric acid (1:1 molar ratio) and heated at 90 °C to afford 3-phenylrhodanine, following the method described in [24]. Subsequently, rhodanine (**1**) and 3-phenylrhodanine (**2**) were condensed with triethyl orthoformate in acetic anhydride to give the corresponding 5-ethoxymethylidene derivatives, 5-ethoxymethylidenerhodanine (**3**) and 5-ethoxymethylidene-3-phenylrhodanine (**4**), according to a previously reported procedure [25]. These activated intermediates were then subjected to nucleophilic condensation with aromatic carboxylic acid hydrazides (**5**–**20**), affording the target rhodanine-hydrazide hybrids (**21**–**52**) in moderate to good yields (47–79%). The overall synthetic route is presented in Scheme 1 and Scheme 2.

The structures of the newly synthesized rhodanine-hydrazide hybrids (**21**–**52**) were confirmed by IR, ^1^H NMR, and ^13^C NMR spectroscopy.

The IR spectra displayed the characteristic absorption bands expected for the synthesized derivatives. Broad bands corresponding to NH stretching vibrations were observed at 3168–3421 cm^−1^, while the carbonyl groups of the rhodanine and hydrazide moieties gave strong absorptions in the range of 1641–1704 cm^−1^. Aromatic C–H stretching vibrations appeared at 3012–3091 cm^−1^, confirming the presence of aromatic substituents. The characteristic C=S stretching band was detected between 1177 and 1235 cm^−1^, with a slight shift toward higher wavenumbers for the 3-phenyl-substituted derivatives compared with the corresponding 3-unsubstituted analogues.

The ^1^H NMR spectra were fully consistent with the proposed structures. Signals corresponding to aromatic protons were observed in the expected region (7.24–9.10 ppm), whereas the exocyclic methine proton (CH=) appeared as a singlet at 7.66–8.07 ppm for the 3-unsubstituted derivatives and at 7.83–7.93 ppm for the 3-phenyl-substituted analogues. The hydrazide NH protons were detected as two characteristic singlets between 9.97 and 11.56 ppm. In compounds bearing an unsubstituted rhodanine nitrogen (**21**–**33** and **47**–**49**), an additional broad singlet corresponding to the rhodanine NH proton was observed at 12.89–13.02 ppm.

The ^13^C NMR spectra further confirmed the proposed structures. Characteristic resonances assigned to the thiocarbonyl carbon (C=S) appeared at 194.9–195.3 ppm for the 3-unsubstituted derivatives and at 193.2–193.7 ppm for the 3-phenyl-substituted analogues. The carbonyl carbon atoms of the rhodanine and hydrazide fragments resonated in the expected ranges of 167.3–169.9 ppm and 163.3–167.2 ppm, respectively, while the exocyclic methine carbon appeared at approximately 93.1 ppm and 90.5 ppm for the corresponding series. In fluorinated derivatives (**28**–**30** and **41**–**43**) characteristic ^13^C-^19^F coupling constants were observed, in agreement with literature data reported for structurally related compounds [26].

Overall, the spectroscopic data were fully consistent with the proposed structures of the synthesized rhodanine-hydrazide hybrids. Detailed IR, ^1^H NMR and ^13^C NMR spectra and signal assignments are provided in the Section 4 and Supplementary Materials (Figures S1–S96).

### 2.2. In Silico Prediction of ADME Parameters

The drug-likeness and predicted pharmacokinetic properties of all synthesized rhodanine-hydrazide hybrids (**21**–**52**) were evaluated using SwissADME web server [27]. The analysis included physicochemical descriptors, Lipinski’s Rule of Five [28], gastrointestinal (GI) absorption, blood–brain barrier (BBB) permeability, P-glycoprotein (P-gp) substrate prediction, aqueous solubility, and bioavailability radar assessment. Complete calculated parameters are provided in Supplementary Table S1, whereas representative bioavailability radar plots [29] are shown in Figures S97 and S98. Summary of the key predicted ADME and drug-likeness properties of the synthesized rhodanine-hydrazide hybrids are summarized in Table 1.

As summarized in Table 1, all synthesized compounds complied with Lipinski’s Rule of Five, indicating favorable drug-likeness and the absence of major physicochemical limitations associated with oral drug candidates. Molecular weights ranged from 280.3 to 434.3 g/mol, the calculated logP values remained within the recommended range, and most compounds were predicted to be soluble or moderately soluble in water, suggesting an overall balanced physicochemical profile. Most derivatives were predicted to exhibit high gastrointestinal absorption, whereas reduced GI absorption was predicted only for the nitro-substituted derivatives (**31**–**33** and **44**–**46**) and the pyridyl-containing analogues (**47**–**49**), most likely due to their increased polarity. None of the synthesized compounds were predicted to cross the blood–brain barrier or to serve as substrates of P-glycoprotein, indicating a low likelihood of central nervous system exposure or transporter-mediated efflux.

### 2.3. Antimicrobial Activity

The antimicrobial activity of the synthesized rhodanine-hydrazide hybrids (**21**–**52**) was evaluated against a panel of Gram-positive and Gram-negative bacteria, as well as *Candida* spp. The complete MIC, MBC, and MFC data are provided in Tables S2 and S3, whereas Table 2 and Table 3 summarize the most active compounds identified in this study.

Overall, the investigated compounds exhibited a clear preference for Gram-positive bacteria, while their activity against Gram-negative organisms was negligible. Most active derivatives inhibited Gram-positive strains at MIC values between 31.3 and 125 µg/mL, while MIC values against Gram-negative bacteria generally exceeded 1000 µg/mL, indicating poor efficacy against these organisms.

As summarized in Table 2, *Micrococcus luteus* was the most susceptible bacterial species. Compounds **36**, **40**, and **50** exhibited the highest antibacterial activity (MIC = 31.3 µg/mL), whereas compounds **35**, **39**, **43**, and **46** inhibited *M. luteus* at 62.5 µg/mL. Good activity was also observed against *Staphylococcus epidermidis* and *Enterococcus faecalis*, particularly for compounds **40** and **46**, which displayed MIC values of 31.3–62.5 µg/mL. By contrast, *Bacillus cereus* was consistently less susceptible, with MIC values typically ranging from 125 to 1000 µg/mL.

Comparison of the active derivatives revealed clear preliminary structure–activity relationships. Incorporation of a 3-phenyl substituent generally enhanced antibacterial potency relative to the corresponding unsubstituted analogues. Moreover, derivatives bearing *para*-halogen (Cl and Br) or *para*-nitro substituents on the benzohydrazide moiety consistently belonged to the most active compounds in the series. Conversely, replacement of the phenyl ring with a pyridyl group, particularly in compounds **47**–**49**, resulted in a marked loss of antibacterial activity.

The investigated compounds exhibited very limited activity against Gram-negative bacteria. Only compounds **44** and **50** showed weak inhibitory effects (MIC = 500–1000 µg/mL), whereas all remaining derivatives were inactive against *Escherichia coli*, *Salmonella typhimurium*, *Proteus mirabilis*, *Klebsiella pneumoniae*, and *Pseudomonas aeruginosa*. This pronounced difference in susceptibility is consistent with the well-established permeability barrier provided by the outer membrane of Gram-negative bacteria, which limits intracellular accumulation of many antimicrobial agents.

Moderate antifungal activity was also observed (Table 3). Among the tested strains, *Candida parapsilosis* was the most susceptible, with compounds **34** and **44** displaying MIC values of 15.6 µg/mL. Several additional derivatives (**27**, **32**, **33**, **37**, **38, 43**, **45**, **50**, and **52**) inhibited this species at 31.3 µg/mL. In contrast, activity against *Candida albicans* was moderate (MIC = 125–500 µg/mL), whereas *Candida glabrata* was considerably less susceptible, requiring substantially higher inhibitory concentrations.

Analysis of MBC/MIC and MFC/MIC ratios (Tables S2 and S3) demonstrated that most active compounds exerted a bactericidal effect against Gram-positive bacteria (MBC/MIC ≤ 4). In contrast, antifungal activity was predominantly fungistatic, as reflected by MFC/MIC ratios exceeding 4 for the majority of active derivatives.

Overall, compounds **36**, **40**, **44**, **46**, and **50** emerged as the most active compounds of the series, combining the highest antibacterial activity against Gram-positive pathogens with moderate antifungal potency. These findings indicate that 3-phenyl-substituted rhodanine-hydrazide derivatives bearing electron-withdrawing *para* substituents constitute the most favorable structural motif for compounds worthy of further investigation and optimization.

### 2.4. Structure–Activity Relationship (SAR)

The biological evaluation revealed several important structure–activity relationships within the investigated series. Firstly, the introduction of a 3-phenyl substituent (compounds **34**–**46** and **50**–**52**) substantially improved antimicrobial activity, indicating that increased lipophilicity and the presence of an additional aromatic ring favor interactions with the biological target.

Secondly, the nature and substitution pattern of the benzohydrazide moiety also had a pronounced influence on antimicrobial potency. Among the halogenated derivatives (Cl, Br, and F), a clear positional effect was observed, with *para*-substituted analogues (e.g., **23**, **27**, **36** or **40**) consistently displaying higher activity than the corresponding *meta-* (**22**, **26**, **35**, and **39**) and *ortho*-substituted derivatives (**21**, **25**, **28**, **34** or **41**). This trend suggests that both steric and electronic factors associated with the substitution pattern play an important role in determining biological activity. Notably, the introduction of a second chlorine atom in 2,4-dichloro derivatives (**24** and **37**) did not result in further enhancement of antimicrobial efficacy, suggesting that additional halogenation may disrupt the optimal molecular arrangement or introduce unfavorable steric interactions.

The presence of electron-withdrawing substituents was generally associated with increased antimicrobial activity. In particular, *para*-nitro derivatives (**33** and **46**) were among the most potent compounds in the series, while *para*-halogenated analogues, especially those bearing chlorine or bromine atoms, also demonstrated consistently the most pronounced antibacterial activity. These observations suggest that both the electronic properties and the substitution pattern of the aromatic ring contribute significantly to the observed biological activity.

**Figure 2:**
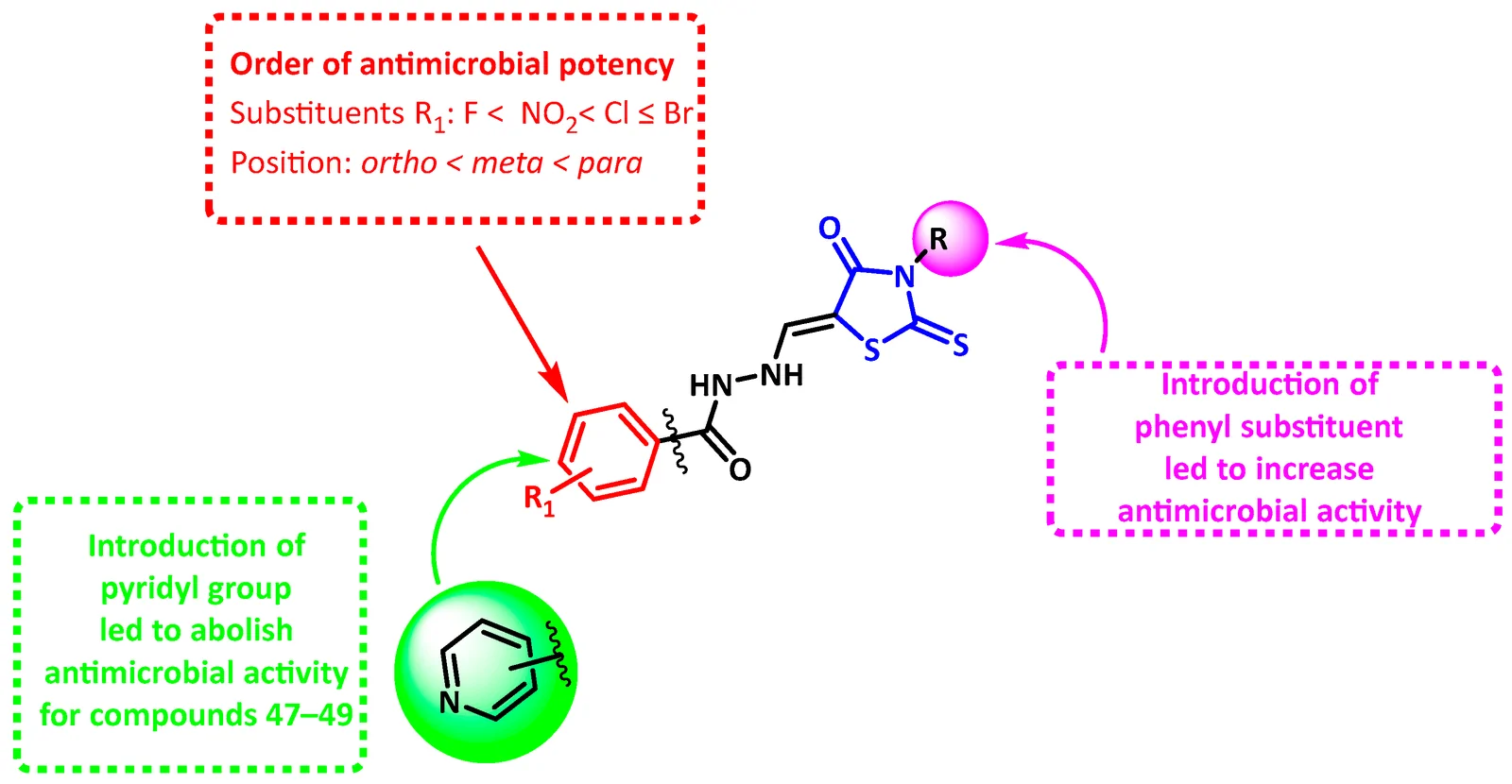
SAR for antimicrobial activity of rhodanine-hydrazide hybrids (**21**–**52**).

Modification of the terminal phenyl ring by replacement with a pyridyl moiety produced divergent effects on biological activity. The unsubstituted pyridyl derivatives (**47**–**49**) displayed negligible activity against Gram-positive bacteria, whereas the corresponding 3-phenyl analogues (**50**–**52**) maintained moderate (**51** and **52**) to strong (**50**) antibacterial activity. This finding indicates that the hydrophobic aromatic character of the phenyl substituent may be more favorable for antibacterial activity than the introduction of a heteroaromatic pyridyl ring within this chemical scaffold. The major SAR relationships identified for this series are summarized in Figure 2.

### 2.5. Antitubercular Activity

The presence of a pyridyl moiety within the investigated scaffold is particularly noteworthy, as nitrogen-containing heteroaromatic rings, including pyridine and pyrazine, represent important structural motifs found in clinically used antitubercular agents, such as isoniazid (INH) and pyrazinamide [30,31]. Considering that several compounds from this series exhibited promising antibacterial activity, their evaluation against *Mycobacterium tuberculosis* was undertaken to determine whether these structural modifications could also confer antimycobacterial properties.

The antitubercular activity of the tested compounds (**47**–**52**) was evaluated against *M. tuberculosis* H_37_Rv, a drug-sensitive reference strain, as well as clinically relevant resistant strains, including a multidrug-resistant (MDR) strain resistant to isoniazid and rifampicin and an isoniazid (INH)-monoresistant strain.

Among the tested derivatives, compound **47** exhibited the highest activity against the reference strain and the drug-sensitive isolate (*M. tuberculosis* 192), with MIC values of 64 µg/mL. However, its potency decreased against resistant strains, with MIC values increasing to 128 µg/mL for the INH-monoresistant strain and 256 µg/mL for the MDR strain.

Compounds **49** and **52** demonstrated moderate but consistent antimycobacterial activity across all tested strains, with MIC values of 128 µg/mL, regardless of resistance profile (Table 4). This observation suggests that their antibacterial activity against *M. tuberculosis* may be retained despite the presence of resistance mechanisms affecting first-line antitubercular drugs.

Slightly lower activity was observed for compound **48**, with MIC values of 256 µg/mL against all strains. Similarly, compound **50** exhibited moderate activity against susceptible strains (MIC = 128 µg/mL), while its efficacy decreased against resistant isolates (MIC = 256 µg/mL). In contrast, **51** was the least active compound, with MIC values of 512 µg/mL across all tested strains.

As expected, the reference drugs showed substantially higher potency against susceptible strains. Isoniazid and rifampicin (RIF) exhibited very low MIC values (0.0625–0.125 µg/mL) against H_37_Rv and the drug-sensitive isolate. In contrast, their activity was markedly reduced against resistant strains, with isoniazid displaying significantly decreased potency against the MDR strain (MIC = 32 µg/mL), while rifampicin was ineffective against this strain (MIC = 512 µg/mL).

Overall, although the investigated compounds exhibited considerably lower potency compared with established antitubercular drugs, several derivatives maintained measurable activity against resistant *M. tuberculosis* strains. Notably, compounds **49** and **52** bearing a 4-pyridyl substituent, together with compound **47** containing a 2-pyridyl moiety, represent promising starting points for further structural optimization aimed at improving antimycobacterial potency.

## 3. Discussion

The growing prevalence of antimicrobial resistance continues to stimulate the search for novel antibacterial scaffolds capable of overcoming existing resistance mechanisms. In the present study, a series of rhodanine-hydrazide hybrids (**21**–**52**) was synthesized and evaluated for their antimicrobial, antifungal, and antimycobacterial activities. The results confirm that structural modifications within the rhodanine scaffold significantly influence antimicrobial activity and provide valuable insights into the structure–activity relationships of this class of compounds.

The synthesized derivatives demonstrated a clear preference for Gram-positive bacteria over Gram-negative organisms. This selectivity is consistent with previous reports describing rhodanine-containing compounds and is likely associated with fundamental differences in bacterial cell envelope architecture. In Gram-negative bacteria, the outer membrane represents an additional permeability barrier that limits the penetration of many lipophilic molecules, thereby reducing their antibacterial activity. In contrast, the absence of this barrier in Gram-positive bacteria may facilitate intracellular accumulation of the compounds and contribute to the lower MIC values observed in this study. Nevertheless, additional investigations, including permeability and uptake studies, would be required to confirm this hypothesis.

Among the investigated compounds, derivatives bearing a phenyl substituent at position 3 of the rhodanine ring generally exhibited superior antibacterial activity compared with their unsubstituted analogues. The most active compounds, including **36**, **40**, **46**, and **50**, displayed MIC values as low as 31.3 µg/mL against *M. luteus*. The beneficial effect of the additional phenyl ring may be attributed to increasing lipophilicity, which could improve π–π interactions with biological targets or facilitate penetration into bacterial cells. Similar observations have been reported for other rhodanine-based antimicrobial agents, although the precise molecular basis of this effect remains to be established.

The nature and position of substituents within the aromatic hydrazide fragment also had a pronounced impact on antibacterial activity. In particular, halogenated derivatives bearing *para*-substituted chloro and bromo groups exhibited the most favorable antibacterial profiles. Halogen substituents may modulate lipophilicity, electronic distribution, and molecular conformation, thereby influencing interactions with biological macromolecules. The observed trend, in which *para*-substituted derivatives generally outperformed their *ortho*-substituted analogues, suggests that steric factors significantly influence biological activity. *Ortho* substitution may hinder optimal molecular orientation within the binding site, whereas *para* substitution allows more favorable interactions while minimizing steric congestion.

Nitro-substituted derivatives, especially compounds **33** and **46**, were also among the most active members of the series. The strong electron-withdrawing nature of the nitro group may enhance interactions with polar amino acid residues through electrostatic effects and hydrogen-bonding interactions. In addition, *para*-nitro substitution appeared more advantageous than *ortho* or *meta* substitution, indicating that both electronic and steric factors contribute to the observed activity profile.

Replacement of the terminal phenyl ring with a pyridyl moiety produced variable biological effects. The pyridyl-containing derivatives lacking a 3-phenyl substituent (**47**–**49**) exhibited little or no activity against Gram-positive bacteria, whereas the corresponding 3-phenyl analogues (**50**–**52**) retained moderate antibacterial activity. These findings suggest that maintaining an appropriate balance between lipophilicity and polar structural features is important for antibacterial efficacy. However, the relative contributions of membrane permeability, target affinity, and other physicochemical properties remain to be elucidated.

The antifungal evaluation revealed moderate but selective activity against *Candida* species, with *C. parapsilosis* being the most susceptible organism. Several compounds, including **34**, **37**, **43**, **44**, **50**, and **52**, displayed MIC values between 15.6 and 31.3 µg/mL. The greater sensitivity of *C. parapsilosis* compared with *C. albicans* and *C. glabrata* likely reflects species-dependent differences in cell wall architecture, membrane permeability, or intracellular targets. The observation that both phenyl-substituted and selected unsubstituted rhodanine derivatives showed antifungal activity suggests that multiple structural features may contribute to activity against fungal pathogens. However, dedicated mechanistic studies would be required to clarify the basis of these differences.

An important aspect of the present study is the antimycobacterial evaluation of pyridyl-containing derivatives. While the tested compounds were substantially less potent than isoniazid and rifampicin against drug-susceptible strains, several derivatives retained measurable activity against drug-resistant *M. tuberculosis* isolates. In particular, compounds **49** and **52** exhibited comparable MIC values across both susceptible and resistant strains, whereas compound **47** showed the greatest activity against susceptible isolates. The preservation of activity against multidrug-resistant and isoniazid-resistant strains may indicate a mechanism of action different from that of first-line antitubercular agents. Given the urgent need for novel antimycobacterial chemotypes, these findings are noteworthy and warrant further investigation.

The in silico ADME analysis further supported the pharmaceutical potential of the synthesized compounds. Most derivatives satisfied Lipinski’s rule of five without violations and were predicted to exhibit predicted gastrointestinal absorption. None of the compounds were predicted to cross the blood–brain barrier, which may reduce the risk of central nervous system-related adverse effects. However, compounds containing nitro or pyridyl substituents showed increased polarity and reduced predicted oral bioavailability, suggesting that future structural optimization should focus on balancing pharmacokinetic properties with antimicrobial potency.

Overall, the present study demonstrates that rhodanine-hydrazide hybrids constitute a promising class of antimicrobial agents with selective activity against Gram-positive bacteria, selected fungal pathogens, and *M. tuberculosis*. The results indicate that introduction of a phenyl substituent at position 3 of the rhodanine scaffold, as well as *para*-halogen or *para*-nitro substitution within the hydrazide fragment, are favorable structural modifications for antimicrobial activity. Future studies should focus on elucidating the molecular mechanisms of action, evaluating cytotoxicity toward mammalian cells, and further optimizing the scaffold to enhance potency against clinically relevant multidrug-resistant pathogens.

## 4. Materials and Methods

### 4.1. General Experimental Details

All reagents and solvents were obtained from commercial suppliers, including Merck (Darmstadt, Germany), Fisher Scientific (Waltham, MA, USA), and Polish Reagents (Gliwice, Poland). The chemicals were employed without any additional purification procedures.

Melting points were measured using an Electrothermal Standard 120 VAC melting point apparatus (Cole-Parmer, Wertheim, Germany) and are presented as observed values without correction. Nuclear magnetic resonance spectra (^1^H and ^13^C NMR) were acquired on a Bruker Avance DPX 300 and Bruker Avance DPX 600 instruments (Billerica, MA, USA), using DMSO-*d*_6_ as the solvent and tetramethylsilane (TMS) as the internal reference. Chemical shifts (δ) are expressed in parts per million (ppm).

Infrared spectra were collected with a Nicolet 6700 FT-IR spectrometer (Thermo Scientific, Philadelphia, PA, USA). Elemental composition analyses were carried out on an AMZ 851 CHX analyzer (PG, Gdańsk, Poland). The experimentally determined elemental contents differed by no more than ±0.4% from the calculated theoretical values.

### 4.2. General Synthetic Procedure of N’-[(4-Oxo-2-thioxo-1,3-thiazolidin-5-ylidene)methyl]benzohydrazides (***21***–***46***) and N’-[(4-Oxo-2-thioxo-1,3-thiazolidin-5-ylidene)methyl]pyridinecarbohydrazides (***47***–***52***)

To 0.003 mol of 5-ethoxymethylidenerhodanine (**3**) or 5-ethoxymethylidene-3-phenylrhodanine (**4**), 0.003 mol of the appropriate aromatic acid hydrazide (**5**–**20**) and 10–15 mL of anhydrous ethanol was added. The reaction mixture was heated under reflux for 30 min to 3 h, then allowed to cool. The resulting precipitate was filtered and dried, and the product was recrystallized from an appropriated solvent.

2-Chloro-N’-[(4-oxo-2-thioxo-1,3-thiazolidin-5-ylidene)methyl]benzohydrazide (**21**)

Yield 55% (AcOH); m.p. = 230–231 °C. ^1^H NMR (600 MHz, DMSO-*d*_6_) δ (ppm): 7.53–7.61 m (5H, 2-Cl-C_6_H_4_ and CH=); 10.13 s, 11.01 s (2H, 2 × NH); 13.00 s (1H, NH rhodanine). ^13^C NMR (150 MHz, DMSO-*d*_6_) δ (ppm): 93.1; 127.9; 129.6; 130.7; 131.1; 132.6; 133.6; 140.5; 166.4; 169.9; 195.0. FT-IR (ν, cm^−1^): 3249 (NH); 3043 (CH_ar_); 1701, 1663 (C=O); 1193 (C=S). Anal. calc. for C_11_H_8_ClN_3_O_2_S_2_ (313.783) (%): C 42.11, H 2.57, N 13.39. Found: C 42.02, H 2.61, N 13.31.

3-Chloro-N’-[(4-oxo-2-thioxo-1,3-thiazolidin-5-ylidene)methyl]benzohydrazide (**22**)

Yield 54% (EtOH); m.p. = 227–228 °C. ^1^H NMR (600 MHz, DMSO-*d*_6_) δ (ppm): 7.63 t, 7.69 s, 7.74 d, 7.88 d (4H, 3-Cl-C_6_H_4_, *J* = 7.8 Hz); 7.93 s (1H, CH=); 10.02 s, 11.16 s (2H, 2 × NH); 12.97 s (1H, NH rhodanine). ^13^C NMR (150 MHz, DMSO-*d*_6_) δ (ppm): 93.1; 126.9; 127.9; 131.4; 132.9; 133.8; 134.1; 140.9; 165.3; 169.8; 195.0. FT-IR (ν, cm^−1^): 3215 (NH); 3077, 3036 (CH_ar_); 1663 (C=O); 1199 (C=S). Anal. calc. for C_11_H_8_ClN_3_O_2_S_2_ (313.783) (%): C 42.11, H 2.57, N 13.39. Found: C 42.01, H 2.59, N 13.34.

4-Chloro-N’-[(4-oxo-2-thioxo-1,3-thiazolidin-5-ylidene)methyl]benzohydrazide (**23**)

Yield 52% (EtOH:water (5:4)); m.p. = 225–226 °C. ^1^H NMR (300 MHz, DMSO-*d*_6_) δ (ppm): 7.65–7.68 m (3H, 4-Cl-C_6_H_4_ i CH=); 7.92 d (2H, 4-Cl-C_6_H_4_, *J* = 8.4 Hz); 9.97 s, 11.09 s (2H, 2 × NH); 12.93 s (1H, NH rhodanine). ^13^C NMR (150 MHz, DMSO-*d*_6_) δ (ppm): 93.1; 129.5; 130.0; 130.6; 138.0; 140.9; 165.7; 169.8; 195.0. FT-IR (ν, cm^−1^): 3415 (NH); 3051 (CH_ar_); 1666 (C=O); 1218 (C=S). Anal. calc. for C_11_H_8_ClN_3_O_2_S_2_ (313.783) (%): C 42.11, H 2.57, N 13.39. Found: C 42.04, H 2.62, N 13.30.

2,5-Dichloro-N’-[(4-oxo-2-thioxo-1,3-thiazolidin-5-ylidene)methyl]benzohydrazide (**24**)

Yield 51% (MeOH); m.p. = 228–230 °C. ^1^H NMR (600 MHz, DMSO-*d*_6_) δ (ppm): 7.66 bs (4H, 2,5-diCl-C_6_H_3_ and CH=); 10.08 s, 11.12 s (2H, 2 × NH); 13.01 s (1H, NH rhodanine). ^13^C NMR (150 MHz, DMSO-*d*_6_) δ (ppm): 93.2; 124.2; 125.1; 129.5; 130.1; 130.5; 132.3; 142.9; 165.0; 169.8; 195.3. FT-IR (ν, cm^−1^): 3245 (NH); 3043 (CH_ar_); 1704, 1662 (C=O); 1200 (C=S). Anal. calc. for C_11_H_7_Cl_2_N_3_O_2_S_2_ (348.228) (%): C 37.94, H 2.03, N 12.07. Found: C 37.86, H 2.09, N 11.98.

2-Bromo-N’-[(4-oxo-2-thioxo-1,3-thiazolidin-5-ylidene)methyl]benzohydrazide (**25**)

Yield 56% (AcOH); m.p. = 231–232 °C. ^1^H NMR (600 MHz, DMSO-*d*_6_) δ (ppm): 7.47–7.49 m, 7.59 s, 7.76 d (5H, 2-Br-C_6_H_4_, and CH=, *J* = 7.9 Hz); 10.17 s, 11.01 s (2H, 2 × NH); 13.00 s (1H, NH rhodanine). ^13^C NMR (150 MHz, DMSO-*d*_6_) δ (ppm): 93.1; 120.0; 128.3; 129.6; 132.6; 133.9; 135.7; 140.3; 167.2; 169.8; 194.9. FT-IR (ν, cm^−1^): 3257 (NH); 3045 (CH_ar_); 1702, 1668 (C=O); 1203 (C=S). Anal. calc. for C_11_H_8_BrN_3_O_2_S_2_ (358.234) (%): C 36.88, H 2.25, N 11.73. Found: C 36.76, H 2.29, N 11.64.

*3*-Bromo-N’-[(4-oxo-2-thioxo-1,3-thiazolidin-5-ylidene)methyl]benzohydrazide (**26**)

Yield 48% (AcOH); m.p. = 227–228 °C. ^1^H NMR (600 MHz, DMSO-*d*_6_) δ (ppm): 7.56 t, 7.69 s, 7.87 d, 7.92 d (4H, 3-Br-C_6_H_4_, *J* = 7.5 Hz); 8.07 s (1H, CH=); 10.01 s, 11.15 s (2H, 2 × NH); 12.97 s (1H, NH rhodanine). ^13^C NMR (150 MHz, DMSO-*d*_6_) δ (ppm): 93.1; 122.5; 127.2; 130.7; 131.6; 134.0; 135.8; 140.9; 165.2; 169.8; 195.0. FT-IR (ν, cm^−1^): 3213 (NH); 3073, 3032 (CH_ar_); 1668 (C=O); 1197 (C=S). Anal. calc. for C_11_H_8_BrN_3_O_2_S_2_ (358.234) (%): C 36.88, H 2.25, N 11.73. Found: C 36.81, H 2.30, N 11.67.

4-Bromo-N’-[(4-oxo-2-thioxo-1,3-thiazolidin-5-ylidene)methyl]benzohydrazide (**27**)

Yield 49% (EtOH:water (4:3)); m.p. = 233–234 °C. ^1^H NMR (300 MHz, DMSO-*d*_6_) δ (ppm): 7.67 s (1H, CH=); 7.82 dd (4H, 4-Br-C_6_H_4_, *J* = 8.8, 13.0 Hz); 9.98 s, 11.09 s (2H, 2 × NH); 12.93 s (1H, NH rhodanine). ^13^C NMR (150 MHz, DMSO-*d*_6_) δ (ppm): 93.1; 127.0; 130.2; 130.9; 132.4; 140.9; 165.8; 169.8; 195.0. FT-IR (ν, cm^−1^): 3411, 3212 (NH); 3051 (CH_ar_); 1664 (C=O); 1213 (C=S). Anal. calc. for C_11_H_8_BrN_3_O_2_S_2_ (358.234) (%): C 36.88, H 2.25, N 11.73. Found: C 36.80, H 2.31, N 11.66.

2-Fluoro-N’-[(4-oxo-2-thioxo-1,3-thiazolidin-5-ylidene)methyl]benzohydrazide (**28**)

Yield 59% (MeOH); m.p. = 233–234 °C. ^1^H NMR (600 MHz, DMSO-*d*_6_) δ (ppm): 7.67 s (1H, CH=); 7.39–7.42 m, 7.65 s, 7.74 td (5H, 2-F-C_6_H_4_, and CH=, *J* = 1.6, 7.4 Hz); 10.04 s, 10.88 s (2H, 2 × NH); 12.97 s (1H, NH rhodanine). ^13^C NMR (150 MHz, DMSO-*d*_6_) δ (ppm): 93.1; 117.0 d (*J* = 21.4 Hz); 121.2; 125.3; 130.7; 134.4; 140.7; 160.8; 164.4; 169.9; 195.2. FT-IR (ν, cm^−1^): 3227 (NH); 3045 (CH_ar_); 1688, 1660 (C=O); 1232 (C=S). Anal. calc. for C_11_H_8_FN_3_O_2_S_2_ (297.329) (%): C 44.44, H 2.71, N 14.13. Found: C 44.32, H 2.76, N 14.03.

3-Fluoro-N’-[(4-oxo-2-thioxo-1,3-thiazolidin-5-ylidene)methyl]benzohydrazide (**29**)

Yield 63% (MeOH); m.p. = 225–226 °C. ^1^H NMR (600 MHz, DMSO-*d*_6_) δ (ppm): 7.67 s (1H, CH=); 7.52 t, 7.63–7.79 m (5H, 3-F-C_6_H_4_, and CH=, *J* = 7.6 Hz); 10.02 s, 11.14 s (2H, 2 × NH); 12.97 s (1H, NH rhodanine). ^13^C NMR (150 MHz, DMSO-*d*_6_) δ (ppm): 93.1; 114.9 d (*J* = 23.0 Hz); 120.0 d (*J* = 20.4 Hz); 124.3; 131.6 d (*J* = 7.0 Hz); 134.1; 140.9; 163.3; 165.3; 169.9; 195.0. FT-IR (ν, cm^−1^): 3217 (NH); 3063, 3044 (CH_ar_); 1668 (C=O); 1182 (C=S). Anal. calc. for C_11_H_8_FN_3_O_2_S_2_ (297.329) (%): C 44.44, H 2.71, N 14.13. Found: C 44.39, H 2.73, N 14.05.

4-Fluoro-N’-[(4-oxo-2-thioxo-1,3-thiazolidin-5-ylidene)methyl]benzohydrazide (**30**)

Yield 47% (AcOH); m.p. = 221–222 °C. ^1^H NMR (600 MHz, DMSO-*d*_6_) δ (ppm): 7.43 t (*J* = 8.6 Hz); 7.99 dd (*J* = 5.7, 8.1 Hz) (4H, 4-F-C_6_H_4_); 7.70 s (1H, CH=); 9.99 s, 11.05 s (2H, 2 × NH); 12.95 s (1H, NH rhodanine). ^13^C NMR (150 MHz, DMSO-*d*_6_) δ (ppm): 93.1; 116.4 d (*J* = 21.8 Hz); 128.3; 130.9 d (*J* = 9.0 Hz); 141.0; 164.2; 165.6; 169.8; 195.1. FT-IR (ν, cm^−1^): 3186 (NH); 3062, 3038 (CH_ar_); 1676 (C=O); 1200 (C=S). Anal. calc. for C_11_H_8_FN_3_O_2_S_2_ (297.329) (%): C 44.44, H 2.71, N 14.13. Found: C 44.34, H 2.77, N 14.08.

2-Nitro-N’-[(4-oxo-2-thioxo-1,3-thiazolidin-5-ylidene)methyl]benzohydrazide (**31**)

Yield 53% (AcOH); m.p. = 223–224 °C. ^1^H NMR (600 MHz, DMSO-*d*_6_) δ (ppm): 7.52 d (*J* = 5.9 Hz); 7.78–7.84 m, 7.97 s, 8.18 d (*J* = 8.2 Hz) (5H, 2-NO_2_-C_6_H_4_ and CH=); 10.28 s, 11.19 s (2H, 2 × NH); 13.02 s (1H, NH rhodanine). ^13^C NMR (150 MHz, DMSO-*d*_6_) δ (ppm): 93.2; 125.1; 129.4; 129.6; 129.8; 132.5; 134.7; 134.9; 147.3; 166.1; 169.8; 194.9. FT-IR (ν, cm^−1^): 3227 (NH); 3051 (CH_ar_); 1672 (C=O); 1197 (C=S). Anal. calc. for C_11_H_8_N_4_O_4_S_2_ (324.336) (%): C 40.74, H 2.49, N 17.27. Found: C 40.64, H 2.53, N 17.17.

3-Nitro-N’-[(4-oxo-2-thioxo-1,3-thiazolidin-5-ylidene)methyl]benzohydrazide (**32**)

Yield 60% (AcOH); m.p. = 233–234 °C. ^1^H NMR (600 MHz, DMSO-*d*_6_) δ (ppm): 7.71 s, 7.91 t, 8.35 d, 8.50 d, 8.73 s (5H, 3-NO_2_-C_6_H_4_ and CH=, *J* = 7.8 Hz); 10.07 s, 11.42 s (2H, 2 × NH); 12.99 s (1H, NH rhodanine). ^13^C NMR (150 MHz, DMSO-*d*_6_) δ (ppm): 93.2; 122.8; 127.6; 127.9; 131.0; 131.3; 134.4; 148.4; 163.6; 169.6; 194.9. FT-IR (ν, cm^−1^): 3240 (NH); 3084, 3068 (CH_ar_); 1695, 1668 (C=O); 1201 (C=S). Anal. calc. for C_11_H_8_N_4_O_4_S_2_ (324.336) (%): C 40.74, H 2.49, N 17.27. Found: C 40.61, H 2.54, N 17.15.

4-Nitro-N’-[(4-oxo-2-thioxo-1,3-thiazolidin-5-ylidene)methyl]benzohydrazide (**33**)

Yield 48% (AcOH); m.p. = 229–230 °C. ^1^H NMR (600 MHz, DMSO-*d*_6_) δ (ppm): 7.70 s (1H, CH=); 8.15 d, 8.43 d (4H, 4-NO_2_-C_6_H_4_, *J* = 8.6 Hz); 10.06 s, 11.38 s (2H, 2 × NH); 12.99 s (1H, NH rhodanine). ^13^C NMR (150 MHz, DMSO-*d*_6_) δ (ppm): 93.3; 124.5; 129.7; 137.3; 140.8; 150.3; 165.1; 169.8; 195.0. FT-IR (ν, cm^−1^): 3280 (NH); 3066 (CH_ar_); 1682 (C=O); 1207 (C=S). Anal. calc. for C_11_H_8_N_4_O_4_S_2_ (324.336) (%): C 40.74, H 2.49, N 17.27. Found: C 40.68, H 2.55, N 17.21.

2-Chloro-N’-[(4-oxo-3-phenyl-2-thioxo-1,3-thiazolidin-5-ylidene)methyl]benzohydrazide (**34**)

Yield 57% (AcOH); m.p. = 208–210 °C. ^1^H NMR (600 MHz, DMSO-*d*_6_) δ (ppm): 7.29 d, 7.48 t, 7.53 t (5H, C_6_H_5_, *J* = 7.6 Hz); 7.59–7.67 m (4H, 2-Cl-C_6_H_4_); 7.83 s (1H, CH=); 10.33 s, 11.15 s (2H, 2 × NH). ^13^C NMR (150 MHz, DMSO-*d*_6_) δ (ppm): 90.5; 127.9; 129.3; 129.4; 129.6; 130.4; 130.8; 131.2; 132.7; 133.6; 136.5; 141.7; 166.6; 167.7; 193.3. FT-IR (ν, cm^−1^): 3315 (NH); 3055 (CH_ar_); 1702, 1678 (C=O); 1235 (C=S). Anal. calc. for C_17_H_12_ClN_3_O_2_S_2_ (389.879) (%): C 52.37, H 3.10, N 10.78. Found: C 52.26, H 3.16, N 10.69.

3-Chloro-N’-[(4-oxo-3-phenyl-2-thioxo-1,3-thiazolidin-5-ylidene)methyl]benzohydrazide (**35**)

Yield 50% (MeOH); m.p. = 197–199 °C. ^1^H NMR (600 MHz, DMSO-*d*_6_) δ (ppm): 7.27 d, 7.47 t, 7.52 t (5H, C_6_H_5_, *J* = 7.6 Hz); 7.66 t, 7.77 d, 7.91–7.94 m, 7.98 s (5H, 3-Cl-C_6_H_4_ and CH=, *J* = 7.3 Hz); 10.22 s, 11.29 s (2H, 2 × NH). ^13^C NMR (150 MHz, DMSO-*d*_6_) δ (ppm): 90.6; 126.9; 127.9; 129.3; 129.4; 129.6; 131.5; 133.0; 133.7; 134.1; 136.5; 142.1; 165.4; 167.6; 193.3. FT-IR (ν, cm^−1^): 3234 (NH); 3048 (CH_ar_); 1698, 1680 (C=O); 1190 (C=S). Anal. calc. for C_17_H_12_ClN_3_O_2_S_2_ (389.879) (%): C 52.37, H 3.10, N 10.78. Found: C 52.24, H 3.15, N 10.73.

4-Chloro-N’-[(4-oxo-3-phenyl-2-thioxo-1,3-thiazolidin-5-ylidene)methyl]benzohydrazide (**36**)

Yield 47% (MeOH:water (2:1)); m.p. = 202–204 °C. ^1^H NMR (600 MHz, DMSO-*d*_6_) δ (ppm): 7.27 d, 7.46 t, 7.52 t (5H, C_6_H_5_, *J* = 7.6 Hz); 7.71 d, 7.98 d (4H, 4-Cl-C_6_H_4_, *J* = 7.9 Hz); 7.91 s (1H, CH=); 10.20 s, 11.25 s (2H, 2 × NH). ^13^C NMR (150 MHz, DMSO-*d*_6_) δ (ppm): 90.5; 129.3; 129.4; 129.5; 129.6; 130.1; 130.5; 136.5; 138.1; 142.2; 165.8; 167.6; 193.3. FT-IR (ν, cm^−1^): 3277 (NH); 3059 (CH_ar_); 1697, 1682 (C=O); 1184 (C=S). Anal. calc. for C_17_H_12_ClN_3_O_2_S_2_ (389.879) (%): C 52.37, H 3.10, N 10.78. Found: C 52.31, H 3.14, N 10.76.

2,5-Dichloro-N’-[(4-oxo-3-phenyl-2-thioxo-1,3-thiazolidin-5-ylidene)methyl]benzohydrazide (**37**)

Yield 72% (MeOH); m.p. = 204–206 °C. ^1^H NMR (600 MHz, DMSO-*d*_6_) δ (ppm):7.30 d, 7.48 t, 7.53 t (5H, C_6_H_5_, *J* = 7.5 Hz); 7.69s, 7.86 bs (4H, 2,5-diCl-C_6_H_3_ and CH=); 10.26 s, 11.25 s (2H, 2 × NH). ^13^C NMR (150 MHz, DMSO-*d*_6_) δ (ppm): 90.7; 122.1; 124.7; 129.3; 129.4; 129.6; 130.1; 130.3; 132.3; 132.6; 136.5; 141.5; 165.1; 167.6; 193.2. FT-IR (ν, cm^−1^): 3316 (NH); 3055 (CH_ar_); 1682 (C=O); 1233 (C=S). Anal. calc. for C_17_H_11_Cl_2_N_3_O_2_S_2_ (424.324) (%): C 48.12, H 2.61, N 9.90. Found: C 48.01, H 2.65, N 9.78.

2-Bromo-N’-[(4-oxo-3-phenyl-2-thioxo-1,3-thiazolidin-5-ylidene)methyl]benzohydrazide (**38**)

Yield 54% (MeOH); m.p. = 132–134 °C. ^1^H NMR (600 MHz, DMSO-*d*_6_) δ (ppm): 7.29 d, 7.46–7.54 m, 7.64 bs, 7.78–7.81 m (10H, C_6_H_5_, 2-Br-C_6_H_4_ and CH=, *J* = 7.5 Hz); 10.37 s, 11.15 s (2H, 2 × NH). ^13^C NMR (150 MHz, DMSO-*d*_6_) δ (ppm): 90.5; 128.4; 129.3; 129.4; 129.6; 132.8; 133.4; 134.0; 136.5; 141.9; 144.5; 146.2; 165.6; 167.3; 193.7. FT-IR (ν, cm^−1^): 3204 (NH); 3055 (CH_ar_); 1678 (C=O); 1224 (C=S). Anal. calc. for C_17_H_12_BrN_3_O_2_S_2_ (434.330) (%): C 47.01, H 2.78, N 9.67. Found: C 46.92, H 2.83, N 9.61.

3-Bromo-N’-[(4-oxo-3-phenyl-2-thioxo-1,3-thiazolidin-5-ylidene)methyl]benzohydrazide (**39**)

Yield 56% (MeOH); m.p. = 192–194 °C. ^1^H NMR (600 MHz, DMSO-*d*_6_) δ (ppm): 7.27 d, 7.46 t, 7.52 t (5H, C_6_H_5_, *J* = 7.6 Hz); 7.59 t, 7.89–7.97 m, 8.12 s (5H, 3-Br-C_6_H_4_ and CH=, *J* = 5.8 Hz); 10.22 s, 11.29 s (2H, 2 × NH). ^13^C NMR (150 MHz, DMSO-*d*_6_) δ (ppm): 90.6; 127.3; 129.2; 129.5; 129.7; 130.8; 131.7; 136.4; 163.5; 167.6; 193.2. FT-IR (ν, cm^−1^): 3235 (NH); 3049 (CH_ar_); 1698, 1678 (C=O); 1227 (C=S). Anal. calc. for C_17_H_12_BrN_3_O_2_S_2_ (434.330) (%): C 47.01, H 2.78, N 9.67. Found: C 46.89, H 2.84, N 9.57.

4-Bromo-N’-[(4-oxo-3-phenyl-2-thioxo-1,3-thiazolidin-5-ylidene)methyl]benzohydrazide (**40**)

Yield 49% (MeOH); m.p. = 212–214 °C. ^1^H NMR (600 MHz, DMSO-*d*_6_) δ (ppm): 7.26 d, 7.46 t, 7.52 t (5H, C_6_H_5_, *J* = 7.6 Hz); 7.84–7.90 m (5H, 4-Br-C_6_H_4_ and CH=); 10.20 s, 11.25 s (2H, 2 × NH). ^13^C NMR (150 MHz, DMSO-*d*_6_) δ (ppm): 90.5; 129.3; 129.4; 129.6; 130.2; 130.9; 132.5; 136.2; 136.5; 142.2; 165.9; 167.6; 193.3. FT-IR (ν, cm^−1^): 3288 (NH); 3054 (CH_ar_); 1702, 1678 (C=O); 1234 (C=S). Anal. calc. for C_17_H_12_BrN_3_O_2_S_2_ (434.330) (%): C 47.01, H 2.78, N 9.67. Found: C 46.95, H 2.81, N 9.60.

2-Fluoro-N’-[(4-oxo-3-phenyl-2-thioxo-1,3-thiazolidin-5-ylidene)methyl]benzohydrazide (**41**)

Yield 49% (MeOH:water (2:1)); m.p. = 208–209 °C. ^1^H NMR (300 MHz, DMSO-*d*_6_) δ (ppm): 7.27 d, 7.39–7.54 m, 7.64–7.84 m (10H, C_6_H_5_, 2-F-C_6_H_4_ and CH=, *J* = 7.4 Hz); 10.24 s, 11.01 s (2H, 2 × NH). ^13^C NMR (150 MHz, DMSO-*d*_6_) δ (ppm): 90.5; 117.3; 118.2; 124.1; 125.4; 129.3; 129.4; 129.6; 130.3; 130.7; 134.6; 136.5; 141.9; 164.5; 167.7; 193.5. FT-IR (ν, cm^−1^): 3267 (NH); 3046 (CH_ar_); 1698, 1675 (C=O); 1229 (C=S). Anal. calc. for C_17_H_12_FN_3_O_2_S_2_ (373.424) (%): C 54.68, H 3.24, N 11.25. Found: C 54.56, H 3.28, N 11.17.

3-Fluoro-N’-[(4-oxo-3-phenyl-2-thioxo-1,3-thiazolidin-5-ylidene)methyl]benzohydrazide (**42**)

Yield 47% (MeOH:water (2:1)); m.p. = 191–194 °C. ^1^H NMR (300 MHz, DMSO-*d*_6_) δ (ppm): 7.27 d, 7.47 t, 7.52 t (5H, C_6_H_5_, *J* = 7.6 Hz); 7.54–7.57 m, 7.68–7.75 m, 7.83 d (4H, 3-F-C_6_H_4_, *J* = 7.0 Hz); 7.92 s (1H, CH=); 10.22 s, 11.27 s (2H, 2 × NH). ^13^C NMR (150 MHz, DMSO-*d*_6_) δ (ppm): 90.6; 114.9 d (*J* = 23.6 Hz); 120.1; 124.4; 129.3; 129.4; 129.6; 130.3; 131.7; 134.0; 136.5; 142.2; 163.3; 167.6; 193.3. FT-IR (ν, cm^−1^): 3235 (NH); 3048 (CH_ar_); 1683 (C=O); 1230 (C=S). Anal. calc. for C_17_H_12_FN_3_O_2_S_2_ (373.424) (%): C 54.68, H 3.24, N 11.25. Found: C 54.55, H 3.23, N 11.14.

4-Fluoro-N’-[(4-oxo-3-phenyl-2-thioxo-1,3-thiazolidin-5-ylidene)methyl]benzohydrazide (**43**)

Yield 79% (MeOH:water (2:1)); m.p. = 200–202 °C. ^1^H NMR (600 MHz, DMSO-*d*_6_) δ (ppm): 7.27 d, 7.45–7.53 m, 7.91 s, 8.04 s (10H, C_6_H_5_, 4-F-C_6_H_4_ and CH=, *J* = 7.6 Hz); 10.19 s, 11.19 s (2H, 2 × NH). ^13^C NMR (150 MHz, DMSO-*d*_6_) δ (ppm): 90.5; 116.5 d (*J* = 22.4 Hz); 128.3; 129.3; 129.4; 129.6; 130.3; 131.0 d (*J* = 9.2 Hz); 136.5; 142.3; 165.8; 167.6; 193.3. FT-IR (ν, cm^−1^): 3223 (NH); 3014 (CH_ar_); 1673 (C=O); 1231 (C=S). Anal. calc. for C_17_H_12_FN_3_O_2_S_2_ (373.424) (%): C 54.68, H 3.24, N 11.25. Found: C 54.61, H 3.29, N 11.19.

2-Nitro-N’-[(4-oxo-3-phenyl-2-thioxo-1,3-thiazolidin-5-ylidene)methyl]benzohydrazide (**44**)

Yield 57% (MeOH); m.p. = 138–140 °C. ^1^H NMR (600 MHz, DMSO-*d*_6_) δ (ppm): 7.29 d, 7.48 t, 7.53 t (5H, C_6_H_5_, *J* = 7.6 Hz); 7.78 d (*J* = 4.2 Hz), 7.84 bs, 8.01 bs, 8.21 d (*J* = 7.7 Hz) (5H, 2-NO_2_-C_6_H_4_, and CH=); 10.51 s, 11.32 s (2H, 2×NH). ^13^C NMR (150 MHz, DMSO-*d*_6_) δ (ppm): 90.6; 125.1; 129.3; 129.5; 129.6; 132.6; 134.8; 136.5; 143.4; 147.4; 151.2; 154.3; 164.9; 167.5; 193.2. FT-IR (ν, cm^−1^): 3168 (NH); 3040 (CH_ar_); 1679 (C=O); 1232 (C=S). Anal. calc. for C_17_H_12_N_4_O_4_S_2_ (400.432) (%): C 50.99, H 3.02, N 13.99. Found: C 50.91, H 3.07, N 13.91.

3-Nitro-N’-[(4-oxo-3-phenyl-2-thioxo-1,3-thiazolidin-5-ylidene)methyl]benzohydrazide (**45**)

Yield 68% (BuOH); m.p. = 132–134 °C. ^1^H NMR (600 MHz, DMSO-*d*_6_) δ (ppm): 7.27 d, 7.47 t, 7.52 t (5H, C_6_H_5_, *J* = 7.5 Hz); 7.93 s (1H, CH=); 8.40 d (*J* = 5.9 Hz), 8.52 d (*J* = 7.3 Hz), 8.78 s (4H, 3-NO_2_-C_6_H_4_); 10.28 s, 11.56 s (2H, 2 × NH). ^13^C NMR (150 MHz, DMSO-*d*_6_) δ (ppm): 90.7; 117.7; 122.8; 127.7; 129.2; 129.5; 129.7; 131.3; 134.4; 136.4; 142.0; 148.5; 163.6; 167.7; 193.2. FT-IR (ν, cm^−1^): 3170 (NH); 2960 (CH_ar_); 1677 (C=O); 1229 (C=S). Anal. calc. for C_17_H_12_N_4_O_4_S_2_ (400.432) (%): C 50.99, H 3.02, N 13.99. Found: C 50.90, H 3.06, N 13.89.

4-Nitro-N’-[(4-oxo-3-phenyl-2-thioxo-1,3-thiazolidin-5-ylidene)methyl]benzohydrazide (**46**)

Yield 55% (MeOH); m.p. = 199–201 °C. ^1^H NMR (600 MHz, DMSO-*d*_6_) δ (ppm): 7.27 d, 7.47 t, 7.52 t (5H, C_6_H_5_, *J* = 7.4 Hz); 7.92 s (1H, CH=); 8.20 d, 8.46 d (4H, 4-NO_2_-C_6_H_4_, *J* = 7.7 Hz); 10.27 s, 11.52 s (2H, 2 × NH). ^13^C NMR (150 MHz, DMSO-*d*_6_) δ (ppm): 90.6; 124.6; 129.3; 129.4; 129.6; 129.7; 136.5; 137.3; 141.9; 150.3; 165.2; 167.6; 193.2. FT-IR (ν, cm^−1^): 3168 (NH); 3012 (CH_ar_); 1664 (C=O); 1232 (C=S). Anal. calc. for C_17_H_12_N_4_O_4_S_2_ (400.432) (%): C 50.99, H 3.02, N 13.99. Found: C 50.89, H 3.03, N 13.94.

N’-[(4-Oxo-2-thioxo-1,3-thiazolidin-5-ylidene)methyl]pyridine-2-carbohydrazide (**47**)

Yield 47% (AcOH); m.p. = 239–240 °C. ^1^H NMR (600 MHz, DMSO-*d*_6_) δ (ppm): 7.66 s (1H, CH=); 7.71 t, 8.05–8.09 m, 8.75 s (4H, 2-C_5_H_4_N, *J* = 5.3 Hz); 10.01 s, 11.34 s (2H, 2 × NH); 12.89 s (1H, NH rhodanine). ^13^C NMR (150 MHz, DMSO-*d*_6_) δ (ppm): 93.0; 123.3; 128.0; 138.5; 141.1; 148.9; 149.5; 164.3; 169.9; 195.2. FT-IR (ν, cm^−1^): 3376 (NH); 3083, 3043 (CH_ar_); 1649 (C=O); 1177 (C=S). Anal. calc. for C_10_H_8_N_4_O_2_S_2_ (280.326) (%): C 42.85, H 2.88, N 19.99. Found: C 42.74, H 2.94, N 19.82.

N’-[(4-Oxo-2-thioxo-1,3-thiazolidin-5-ylidene)methyl]pyridine-3-carbohydrazide (**48**)

Yield 48% (AcOH); m.p. = 176–178 °C. ^1^H NMR (600 MHz, DMSO-*d*_6_) δ (ppm): 7.67 s (1H, CH=); 7.63 dd (*J* = 5.0, 7.8 Hz), 8.25 d (*J* = 7.8 Hz), 8.82 dd (*J* = 1.5, 5.0 Hz), 9.06 d (*J* = 1.5 Hz) (4H, 3-C_5_H_4_N); 10.02 s, 11.22 s (2H, 2 × NH); 12.95 s (1H, NH rhodanine). ^13^C NMR (150 MHz, DMSO-*d*_6_) δ (ppm): 93.2; 124.4; 127.7; 136.0; 140.7; 149.0; 153.5; 165.2; 169.8; 195.0. FT-IR (ν, cm^−1^): 3172 (NH); 3072, 3043 (CH_ar_); 1641 (C=O); 1183 (C=S). Anal. calc. for C_10_H_8_N_4_O_2_S_2_ (280.326) (%): C 42.85, H 2.88, N 19.99. Found: C 42.76, H 2.92, N 19.89.

N’-[(4-Oxo-2-thioxo-1,3-thiazolidin-5-ylidene)methyl]pyridine-4-carbohydrazide (**49**)

Yield 48% (AcOH); m.p. = 191–192 °C. ^1^H NMR (600 MHz, DMSO-*d*_6_) δ (ppm): 7.68 s (1H, CH=); 7.82 d, 8.84 d (4H, 4-C_5_H_4_N, *J* = 3.7 Hz); 10.05 s, 11.34 s (2H, 2 × NH); 12.98 s (1H, NH rhodanine). ^13^C NMR (150 MHz, DMSO-*d*_6_) δ (ppm): 93.3; 121.9; 139.1; 140.6; 151.0; 165.0; 169.8; 194.9. FT-IR (ν, cm^−1^): 3100 (NH); 3091 (CH_ar_); 1687, 1656 (C=O); 1178 (C=S). Anal. calc. for C_10_H_8_N_4_O_2_S_2_ (280.326) (%): C 42.85, H 2.88, N 19.99. Found: C 42.79, H 2.94, N 19.91.

N’-[(4-Oxo-3-phenyl-2-thioxo-1,3-thiazolidin-5-ylidene)methyl]pyridine-2-carbohydrazide (**50**)

Yield 51% (MeOH:water (1:1)); m.p. = 129–132 °C. ^1^H NMR (300 MHz, DMSO-*d*_6_) δ (ppm): 7.24 d, 7.45–7.53 m (5H, C_6_H_5_, *J* = 7.1 Hz); 7.71–7.75 m, 8.06–8.11 m, 8.79 d (4H, 2-C_5_H_4_N, *J* = 3.6 Hz); 7.87 s (1H, CH=); 10.25 s, 11.51 s (2H, 2 × NH). ^13^C NMR (75 MHz, DMSO-*d*_6_) δ (ppm): 90.4; 123.4; 128.1; 129.2; 129.4; 129.6; 136.5; 138.6; 142.4; 148.9; 149.6; 164.4; 167.6; 193.5. FT-IR (ν, cm^−1^): 3335, 3196 (NH); 3092, 3055 (CH_ar_); 1651 (C=O); 1221 (C=S). Anal. calc. for C_16_H_12_N_4_O_2_S_2_ (356.422) (%): C 53.92, H 3.39, N 15.72. Found: C 53.83, H 3.46, N 15.63.

N’-[(4-Oxo-3-phenyl-2-thioxo-1,3-thiazolidin-5-ylidene)methyl]pyridine-3-carbohydrazide (**51**)

Yield 58% (MeOH:water (1:1)); m.p. = 154–156 °C. ^1^H NMR (300 MHz, DMSO-*d*_6_) δ (ppm): 7.26 d, 7.45–7.53 m (5H, C_6_H_5_, *J* = 6.9 Hz); 7.63–7.67 m, 8.29–8.31 m, 8.84 d, 9.10 s (4H, 3-C_5_H_4_N, *J* = 4.5 Hz); 7.89 s (1H, CH=); 10.24 s, 11.38 s (2H, 2 × NH). ^13^C NMR (150 MHz, DMSO-*d*_6_) δ (ppm): 90.6; 124.2; 124.5; 129.3; 129.4; 129.6; 132.5; 136.0; 136.5; 149.1; 153.1; 165.0; 167.6; 193.2. FT-IR (ν, cm^−1^): 3157 (NH); 3068 (CH_ar_); 1679 (C=O); 1231 (C=S). Anal. calc. for C_16_H_12_N_4_O_2_S_2_ (356.422) (%): C 53.92, H 3.39, N 15.72. Found: C 53.84, H 3.42, N 15.66.

N’-[(4-Oxo-3-phenyl-2-thioxo-1,3-thiazolidin-5-ylidene)methyl]pyridine-4-carbohydrazide (**52**)

Yield 54% (MeOH:water (1:1)); m.p. = 194–196 °C. ^1^H NMR (300 MHz, DMSO-*d*_6_) δ (ppm): 7.26 d, 7.43–7.53 m (5H, C_6_H_5_, *J* = 7.7 Hz); 7.88 s, 8.86 d (4H, 4-C_5_H_4_N and CH=, *J* = 9.7 Hz); 10.30 s, 11.52 s (2H, 2 × NH). ^13^C NMR (75 MHz, DMSO-*d*_6_) δ (ppm): 90.6; 122.0; 129.3; 129.4; 129.6; 136.5; 139.4; 141.7; 151.0; 165.2; 167.5; 193.2. FT-IR (ν, cm^−1^): 3421 (NH); 3080 (CH_ar_); 1655 (C=O); 1234 (C=S). Anal. calc. for C_16_H_12_N_4_O_2_S_2_ (356.422) (%): C 53.92, H 3.39, N 15.72. Found: C 53.83, H 3.40, N 15.67.

### 4.3. ADME Properties

The physicochemical characteristics and ADME-related parameters of compounds **21**–**52** were predicted using the online SwissADME platform developed by the Swiss Institute of Bioinformatics (https://www.swissadme.ch/index.php; accessed on 28 June 2026) [32]. The obtained computational data were used to evaluate the drug-likeness properties and pharmacokinetic profiles of the investigated molecules.

### 4.4. Antimicrobial Activity

The antimicrobial activity of the obtained compounds was assessed using the broth microdilution method. For strain cultivation, Mueller–Hinton (MH) broth and MH supplemented with 2% glucose were applied for bacterial and fungal culture, respectively. Minimum inhibitory concentrations (MICs) were established using a set of reference strains from the American Type Culture Collection (ATCC): Gram-positive strains (*Staphylococcus aureus* ATCC 25923, *Staphylococcus aureus* ATCC BAA1707, *Staphylococcus epidermidis* ATCC 12228, *Micrococcus luteus* ATCC 10240, *Bacillus cereus* ATCC 10876, *Enterococcus faecalis* ATCC 29212), Gram-negative strains (*Salmonella typhimurium* ATCC 14028, *Escherichia coli* ATCC 25922, *Proteus mirabilis* ATCC 12453, *Klebsiella pneumoniae* ATCC 13883, *Pseudomonas aeruginosa* ATCC 9027) and yeast strains (*Candida glabrata* ATCC 66032, *Candida albicans* ATCC 102231, *Candida parapsilosis* ATCC 22019). Vancomycin, Ciprofloxacin, and Nystatin served as the standard antibacterial and antifungal drugs.

Stock solutions of the tested compounds were prepared in dimethyl sulfoxide (DMSO) and subsequently subjected to twofold serial dilution in sterile 96-well polystyrene microplates (Nunc, Roskilde, Denmark). This procedure resulted in final concentrations ranging from 1000 to 7.8 mg/L in the test medium. Appropriate DMSO control (at a final concentration of 1%), a strain growth control (inoculum without the tested compounds), and a negative control (the tested compounds ranged from 1000 to 7.8 µg/mL without inoculum) were included on each microplate. Bacteria or yeast suspensions were obtained from 24-h cultures and adjusted in sterile saline to match 0.5 McFarland standard, followed by inoculation into wells to achieve a final density of 5 × 10^5^ CFU/mL for bacteria and 5 × 10^4^ CFU/mL for fungi. Each microplate included positive controls (inoculated medium without compounds) as well as negative controls (medium containing compounds without inoculum).

After incubation at 35 °C for 24 h, bacterial or fungal growth was evaluated by measuring optical density at 600 nm using a BioTEK ELx808 spectrophotometer (Bio-Tek Instruments, Winooski, VT, USA) and was estimated based on difference as much as OD 0.050 between inoculated and without inoculum wells with the same compound concentration. The MIC value was defined as the lowest concentration of the tested compound required to inhibit visible growth. To determine the minimum bactericidal concentration (MBC) and minimum fungicidal concentration (MFC), 5 μL samples from each well, including controls, were plated onto agar media and incubated again at 35 °C for 24 h. The MBC and MFC corresponded to the lowest concentration at which no colony growth was observed. All tests were performed in triplicate, with the highest recorded value reported.

### 4.5. Antimycobacterial Activity

The antimycobacterial properties of the synthesized compounds were evaluated at the Department of Microbiology of the National Tuberculosis and Lung Diseases Research Institute in Warsaw, Poland. In vitro activity was assessed against the reference strain *Mycobacterium tuberculosis* H_37_Rv (ATCC 25618) as well as three clinical isolates obtained from patients with tuberculosis. One isolate (strain 36/26) exhibited resistance to both isoniazid (INH) and rifampicin (RIF), and one (3715) exhibited resistance for INH, whereas the third isolate (strain 192) was susceptible to all routinely administered first-line antitubercular agents.

Clinical strains were recovered from patients undergoing diagnostic evaluation for tuberculosis and were identified and characterized using standard microbiological procedures, including drug susceptibility testing. The isolates selected for this study originated from the mycobacterial strain collection maintained by the National Reference Laboratory of Mycobacteria. Selection criteria were based on their drug-resistance profiles, specifically including one fully susceptible strain and one multidrug-resistant strain. Ethical approval for the collection and experimental use of *M. tuberculosis* isolates was granted by the Bioethics Committee (approval no. KB-55/2014).

The synthesized compounds were tested using the broth microdilution technique in accordance with the Clinical and Laboratory Standards Institute (CLSI) guideline M24, 3rd edition [33]. Assays were carried out in 96-well microtiter plates containing Middlebrook 7H9 broth (Becton Dickinson, Franklin Lakes, NJ, USA) supplemented with Tween 80 (Sigma-Aldrich, St. Louis, MO, USA) and OADC enrichment (oleic acid–albumin–dextrose–catalase). The procedure followed a previously published protocol [34]. Bacterial inocula were prepared from fresh Löwenstein–Jensen cultures, adjusted to a turbidity equivalent to a 0.5 McFarland standard, and subsequently diluted 1:100. Stock solutions of the tested compounds were prepared in dimethyl sulfoxide (DMSO) and serially diluted twofold to obtain final concentrations ranging from 512 to 0.0625 µg/mL. Each well was inoculated with 100 μL of the bacterial suspension. Appropriate growth and sterility controls were included in all experiments. Microplates were incubated at 37 °C for 21 days. Subsequently, Alamar Blue reagent was added to each well, and incubation was continued for an additional 24 h. The minimum inhibitory concentration (MIC) was defined as the lowest concentration of a compound preventing bacterial growth, as indicated by the absence of a color change [35,36]. Isoniazid and rifampicin served as reference antimycobacterial agents.

## 5. Conclusions

A series of novel rhodanine-hydrazide hybrids was successfully synthesized and structurally characterized using spectroscopic methods. Biological evaluation revealed that the investigated compounds exhibited selective antimicrobial activity, with a clear preference for Gram-positive bacteria over Gram-negative strains. Several derivatives, particularly those containing a 3-phenylrhodanine scaffold combined with halogen (**36**, **40**) or nitro substituents (**46**), demonstrated the highest antibacterial potency, with MIC values as low as 31.3 µg/mL against selected Gram-positive microorganisms.

The tested compounds also displayed moderate antifungal activity, especially against *C. parapsilosis*, indicating that the rhodanine-hydrazide framework may serve as a useful platform for the development of new antifungal agents. Furthermore, pyridyl-containing derivatives (**47**, **49**, and **52**) showed measurable activity against both drug-susceptible and drug-resistant *M. tuberculosis* strains, highlighting their potential as promising lead structures for future antitubercular drug discovery.

In silico ADME analysis indicated generally favorable predicted drug-likeness profiles, with most compounds satisfying Lipinski’s rule of five and exhibiting predicted gastrointestinal absorption. Structure–activity relationship studies demonstrated that biological activity was strongly influenced by both the substitution pattern within the hydrazide fragment and the presence of a phenyl group at position 3 of the rhodanine ring.

Overall, the present findings identify rhodanine-hydrazide hybrids exhibiting preliminary antimicrobial activity and provide valuable insights for the rational design of new antibacterial, antifungal, and antimycobacterial agents. Further studies focused on mechanism-of-action investigations, cytotoxicity assessment, and structural optimization are warranted to improve potency and pharmacological properties.

## Figures and Tables

**Table 1:** Key predicted ADME and drug-likeness parameters of the synthesized rhodanine-hydrazide hybrids (**21**–**52**), obtained using the SwissADME web server.

Parameter	Range/Observation
molecular weight	280–434 g/mol
LogP	−0.65 to 3.07
Lipinski violations	none
GI absorption	high for most compounds
BBB permeation	none predicted
P-gp substrate	none predicted
water solubility	soluble to moderately soluble

**Table 2:** Most active rhodanine-hydrazide hybrids against Gram-positive bacteria (MIC, µg/mL).

Compound	*S. epidermidis* ATCC 12228	*M. luteus* ATCC 10240	*E. faecalis* ATCC 29212
**35**	125	62.5	62.5
**36**	125	31.3	62.5
**39**	125	62.5	62.5
**40**	62.5	31.3	31.3
**43**	250	62.5	125
**46**	31.3	62.5	62.5
**50**	62.5	31.3	125

**Table 3:** Most active rhodanine-hydrazide hybrids against *Candida* spp. (MIC, µg/mL).

Compound	*C. glabrata* ATCC 66032	*C. albicans* ATCC 102231	*C. parapsilosis* ATCC 22019
**34**	125	125	15.6
**37**	250	125	31.3
**38**	250	125	31.3
**43**	250	250	31.3
**44**	250	62.5	15.6
**45**	1000	500	31.3
**50**	250	250	31.3
**52**	250	125	31.3

**Table 4:** Antimycobacterial activity of compounds (**47**–**52**) against drug-susceptible and drug-resistant *Mycobacterium tuberculosis* strains (MIC, μg/mL).

Compound	Drug-Susceptible Strains		Drug-Resistant Strains	
	H_37_Rv	192	36/26 (MDR)	3715 (INH-R)
**47**	64	64	256	128
**48**	256	256	256	256
**49**	128	128	128	128
**50**	128	128	256	256
**51**	512	512	512	512
**52**	128	128	128	128
INH	0.0625	0.0625	32	0.5
RIF	0.125	0.125	512	0.125

Abbreviations: H37Rv, reference drug-susceptible *Mycobacterium tuberculosis* strain; 192, drug-susceptible clinical isolate; MDR, multidrug-resistant; INH-R, isoniazid-resistant; INH, isoniazid; RIF, rifampicin.

## Data Availability

The data are contained within the article and Supplementary Materials.

## Supplementary Materials

The following supporting information can be downloaded at: https://www.mdpi.com/article/10.3390/antibiotics15090853/s1, Figures S1–S32. ^1^H NMR spectra of compounds **21**–**52**, Figures S33–S64. ^13^C NMR spectra of compounds **21**–**52**, Figures S65–S96. FT-IR spectra of compounds **21**–**52**, Figure S97. Bioavailability radar for 3-unsubstituted rhodanine–hydrazide derivatives (**21**–**33** and **47**–**49**), Figure S98. Bioavailability radar for 3-substituted rhodanine–hydrazide hybrids (**34**–**46** and **50**–**52**), Table S1. Physicochemical properties of compounds **21**–**52** obtained using the SwissADME server, Table S2. MIC and MBC for rhodanine-hydrazide hybrids (**21**–**52**) against Gram-positive bacteria, Table S3. MIC and MBC or MFC for rhodanine-hydrazide hybrids (**21**–**52**) against Gram-negative bacteria and yeasts strains.
