# Supplementary material for: Design, Synthesis and Biological Evaluation of Rhodanine-Based Hydrazide Derivatives as Antibacterial, Antifungal, and Antitubercular Agents
Source: Antibiotics (Basel). 2026 Sep 1;15(9):853. doi: 10.3390/antibiotics15090853 (PMC13603270; doi:10.3390/antibiotics15090853)
Supplement: Supplementary file 1 [file antibiotics-15-00853-s001.zip › antibiotics-4515559-supplementary.pdf]

Supplementary materials for

Design, synthesis and biological evaluation of rhodanine-based hydrazide derivatives as antibacterial, antifungal, and antitubercular agents

Agata Paneth [1], Izabela Korona-Głowniak [2], Agnieszka Głogowska [3], Jacek Szczepański [1], Dominik Włodarczyk [1], Katarzyna Suśniak [2], Ewa Augustynowicz-Kopeć [3] and Nazar Trotsko [1,*]

[1] Department of Organic Chemistry, Medical University of Lublin, Chodzki 4A, 20-093 Lublin, Poland; agata.paneth@umlub.edu.pl (A.P.); jaacek.szczepanski.93@gmail.com; (J.S.); 54246@umlub.edu.pl (D.W.); nazar.trotsko@umlub.edu.pl (N.T.).

[2] Department of Pharmaceutical Microbiology, Medical University in Lublin, Chodzki 1, 20-093 Lublin, Poland; izabela.korona-glowniak@umlub.edu.pl (I.K.-G.); katarzyna.susniak@umlub.edu.pl (K.S.).

[3] Department of Microbiology, National Tuberculosis and Lung Diseases Research Institute, Plocka 26, 01-138 Warsaw, Poland; a.glogowska@igichp.edu.pl (A.G.); e.kopec@igichp.edu.pl (E.A.-K.)

* Correspondence: nazar.trotsko@umlub.edu.pl.

Table of contents:

1. ^{1}H NMR spectra of compounds **21–52**……………………………………………………..…2
2. ^{13}C NMR spectra of compounds **21–52**…………………………………………………...……18
3. FT-IR spectra of compounds **21–52**……………………………………...……………..……...….34
4. Bioavailability radar for 3-unsubstituted rhodanine–hydrazide derivatives (**21–33** and **47–49**)……………………………………………………………………………………………….50
5. Bioavailability radar for 3-substituted rhodanine–hydrazide hybrids (**34–46** and **50–52**)....…..51
6. Physicochemical properties of compounds **21**–**52** obtained using the SwissADME server………………..52
7. MIC and MBC for rhodanine-hydrazide hybrids (**21**–**52**) against Gram-positive bacteria………………...54
8. MIC and MBC or MFC for rhodanine-hydrazide hybrids (**21**–**52**) against Gram-negative bacteria and yeasts strains……………………………………………………………………………………………..55

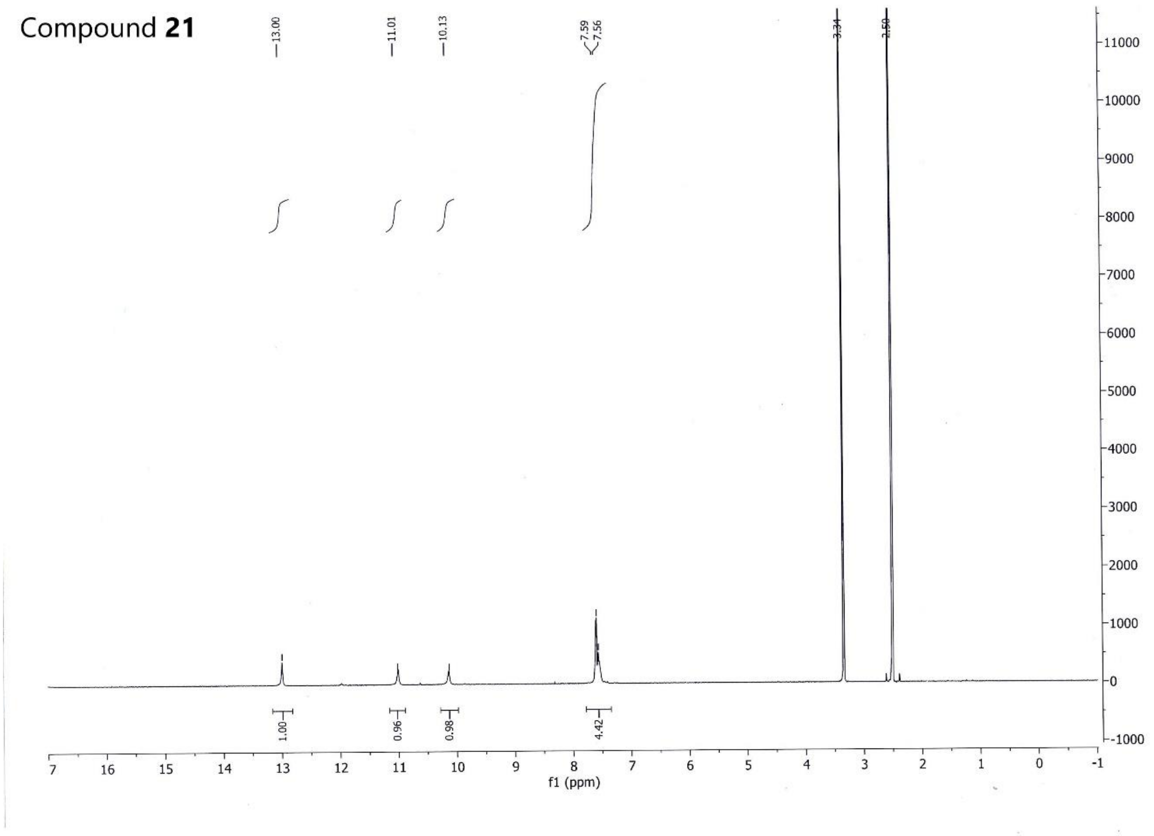

Figure S1. ^{1}H NMR spectrum of compound **21**

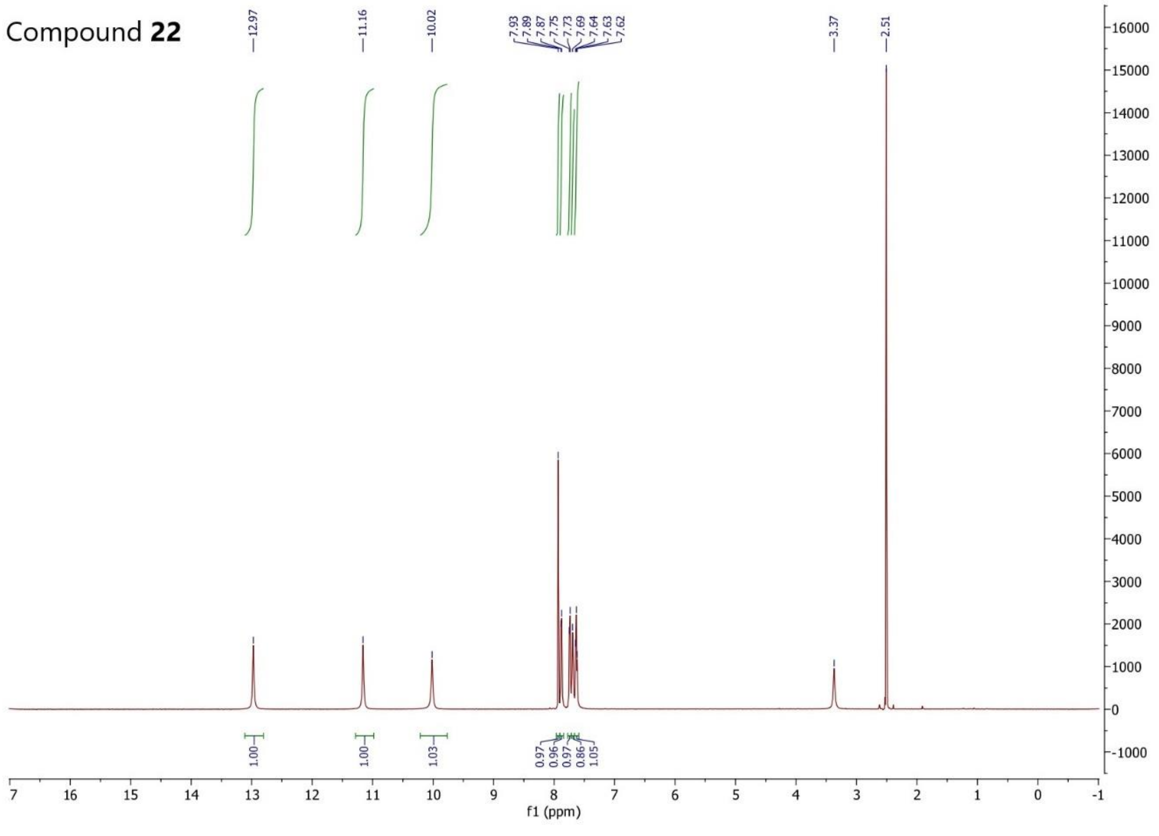

Figure S2. ^{1}H NMR spectrum of compound **22**

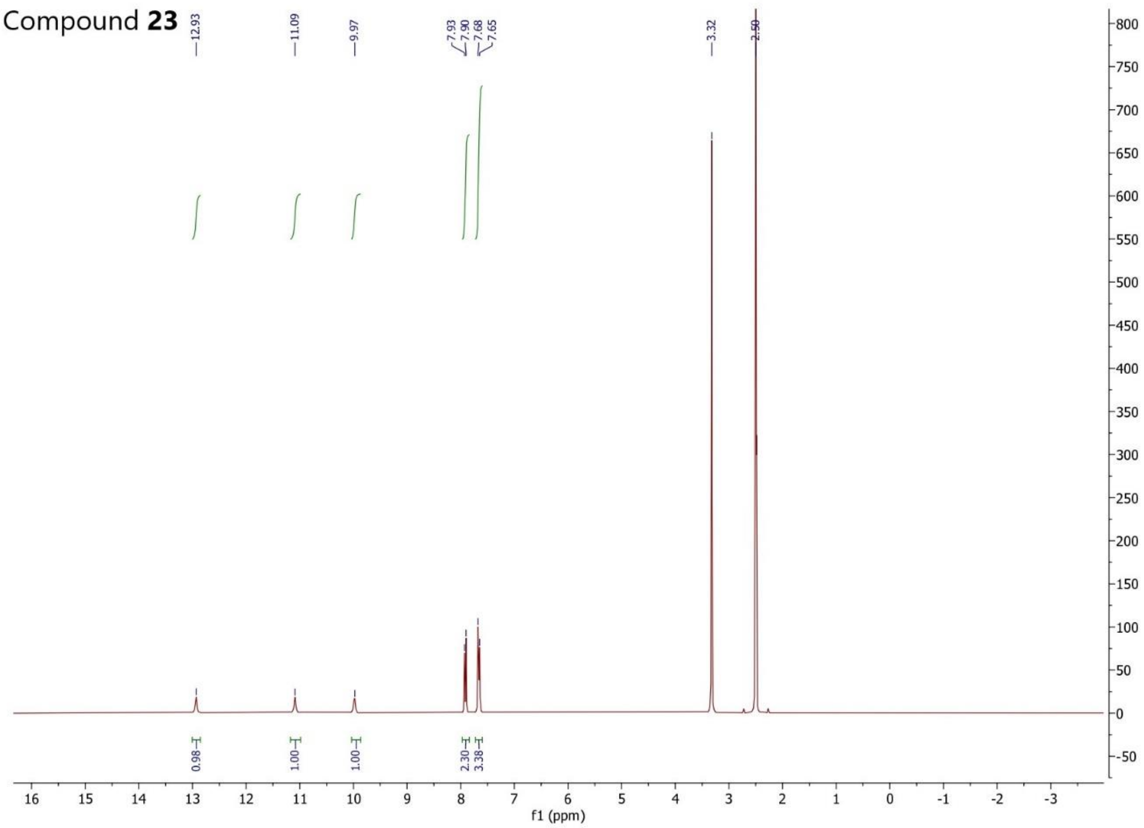

Figure S3. ^{1}H NMR spectrum of compound **23**

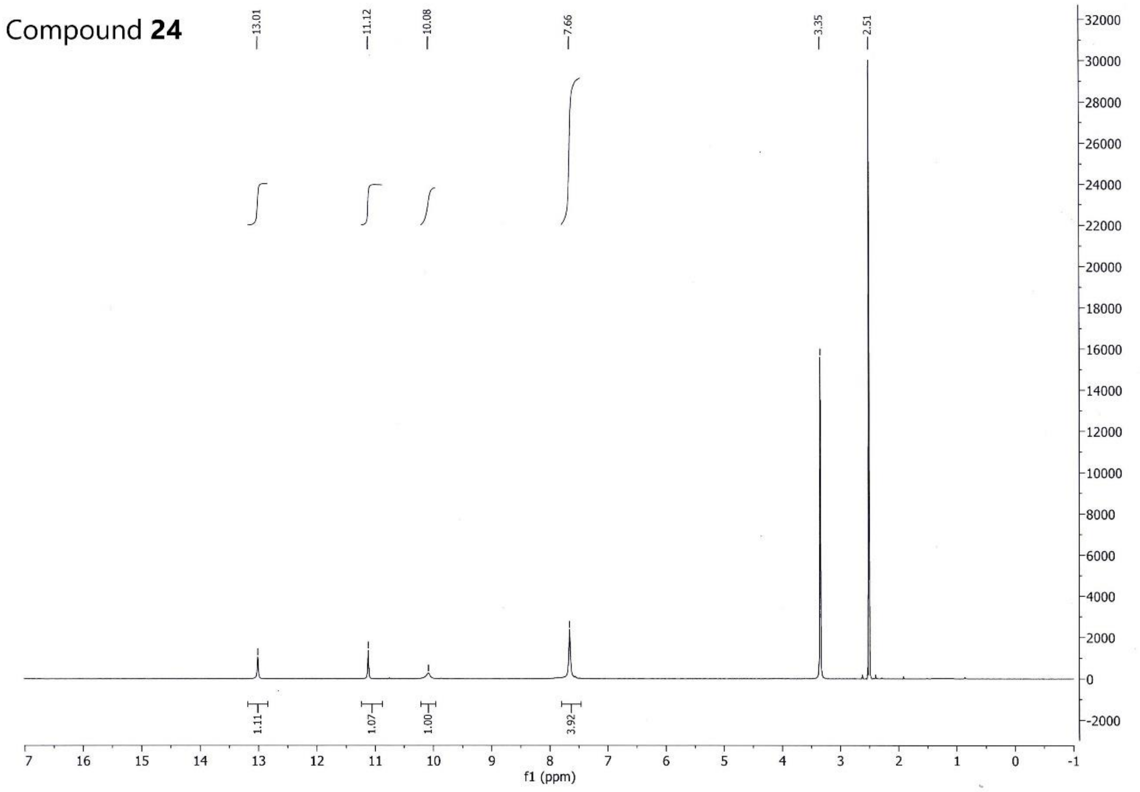

Figure S4. ^{1}H NMR spectrum of compound **24**

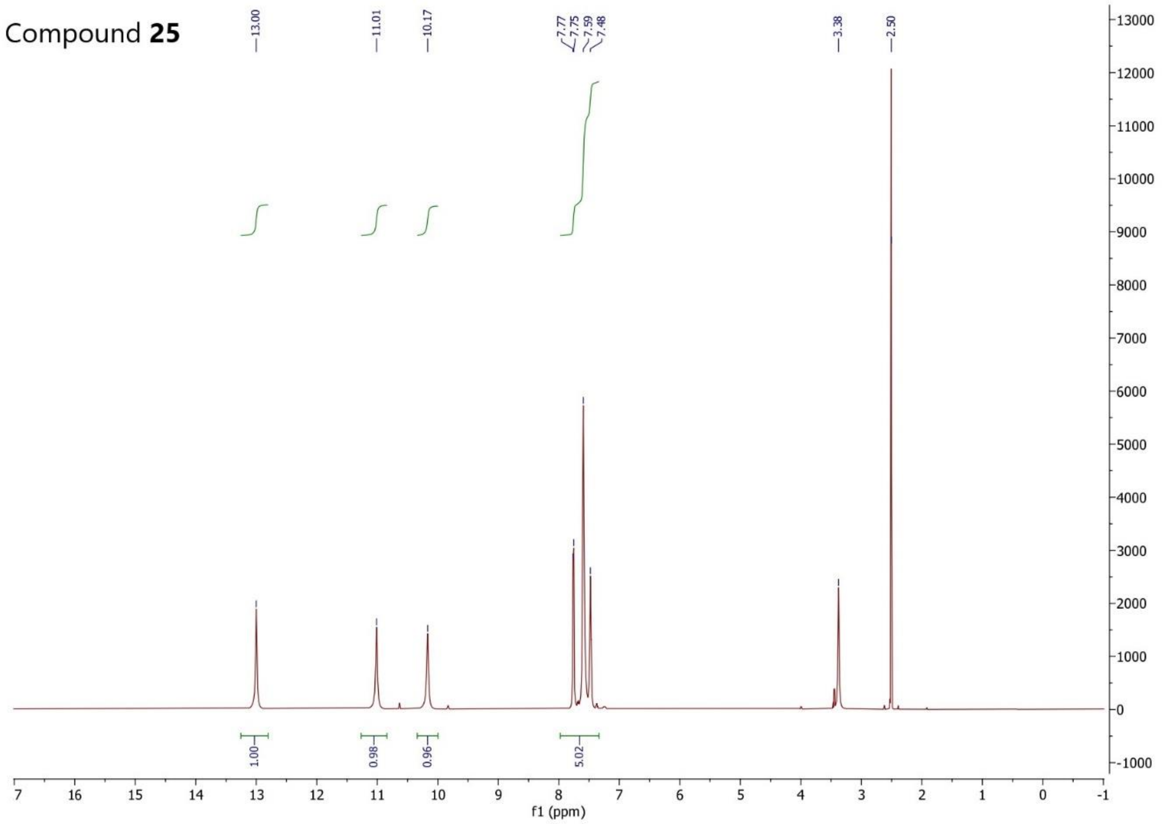

Figure S5. ^{1}H NMR spectrum of compound **25**

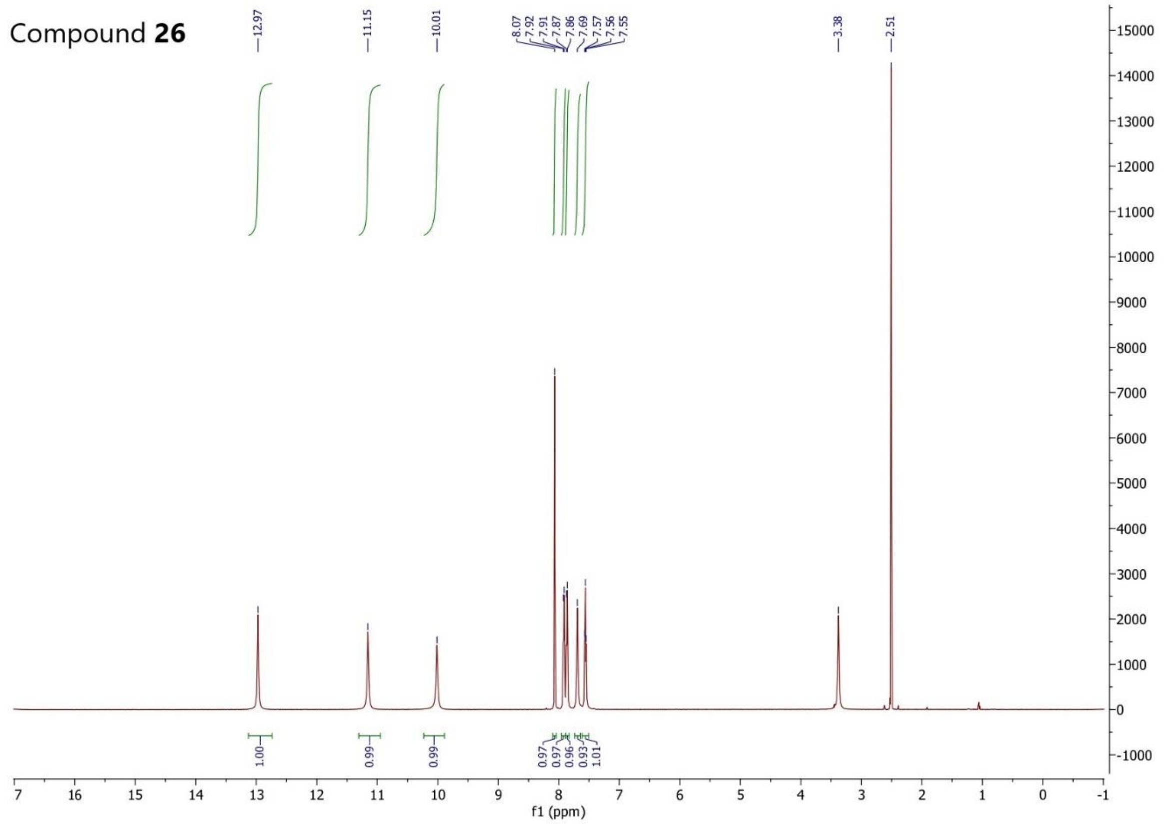

Figure S6. ^{1}H NMR spectrum of compound **26**

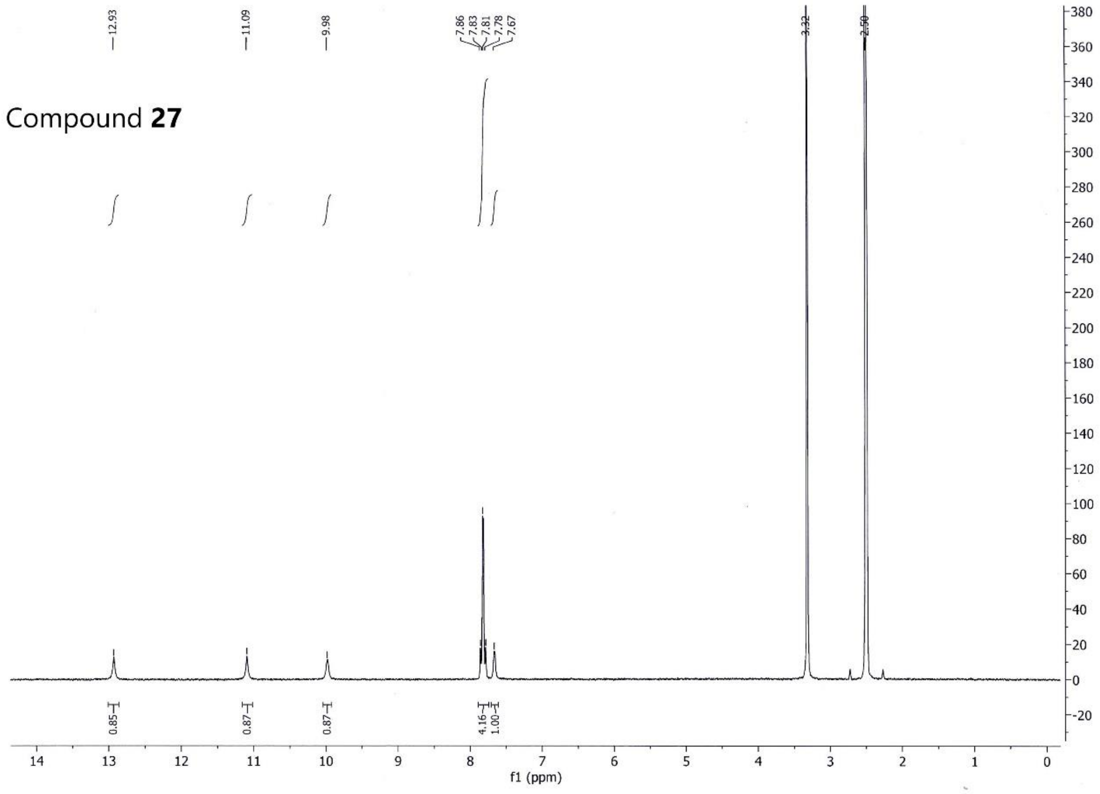

Figure S7. ^{1}H NMR spectrum of compound **27**

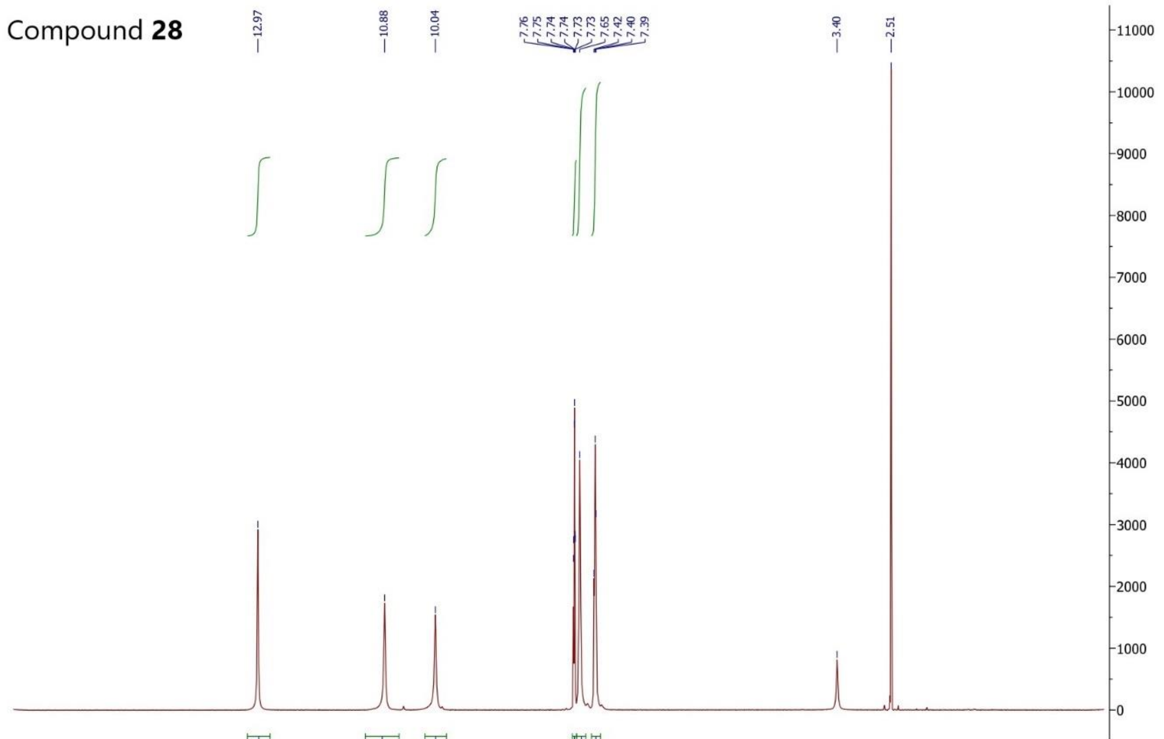

Figure S8. ^{1}H NMR spectrum of compound **28**

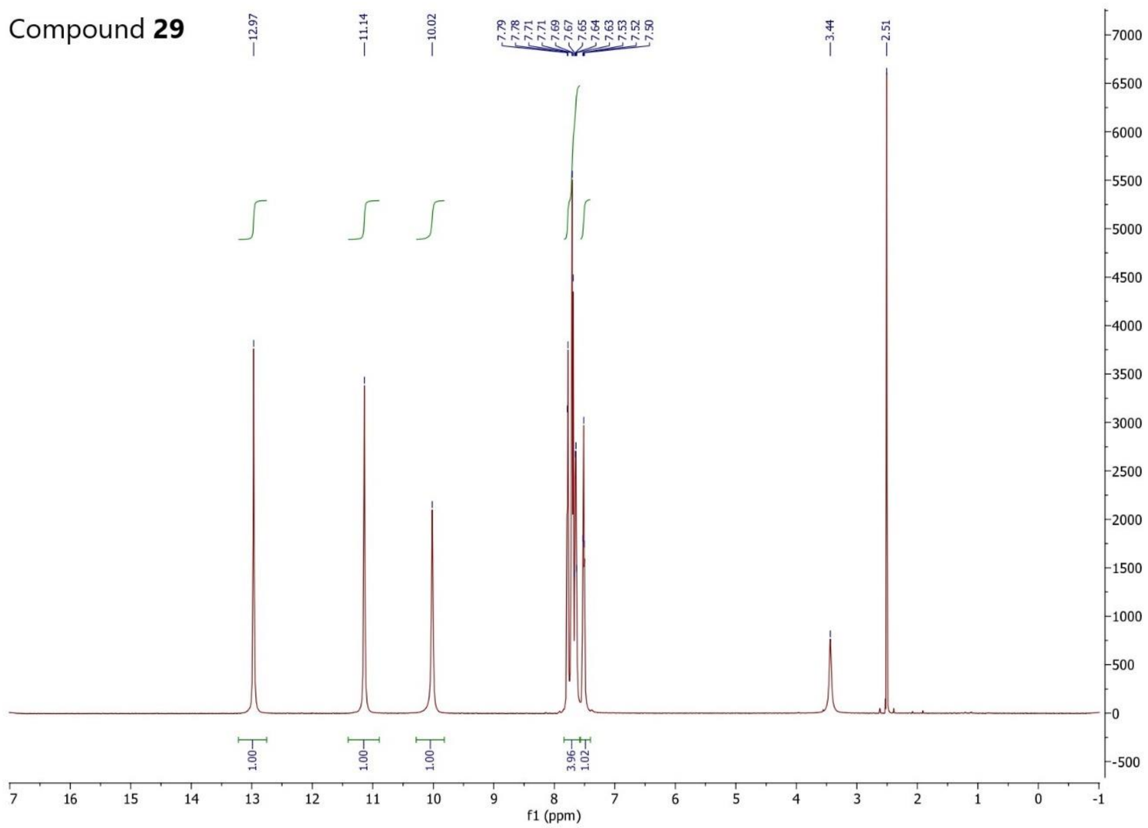

Figure S9. ^{1}H NMR spectrum of compound **29**

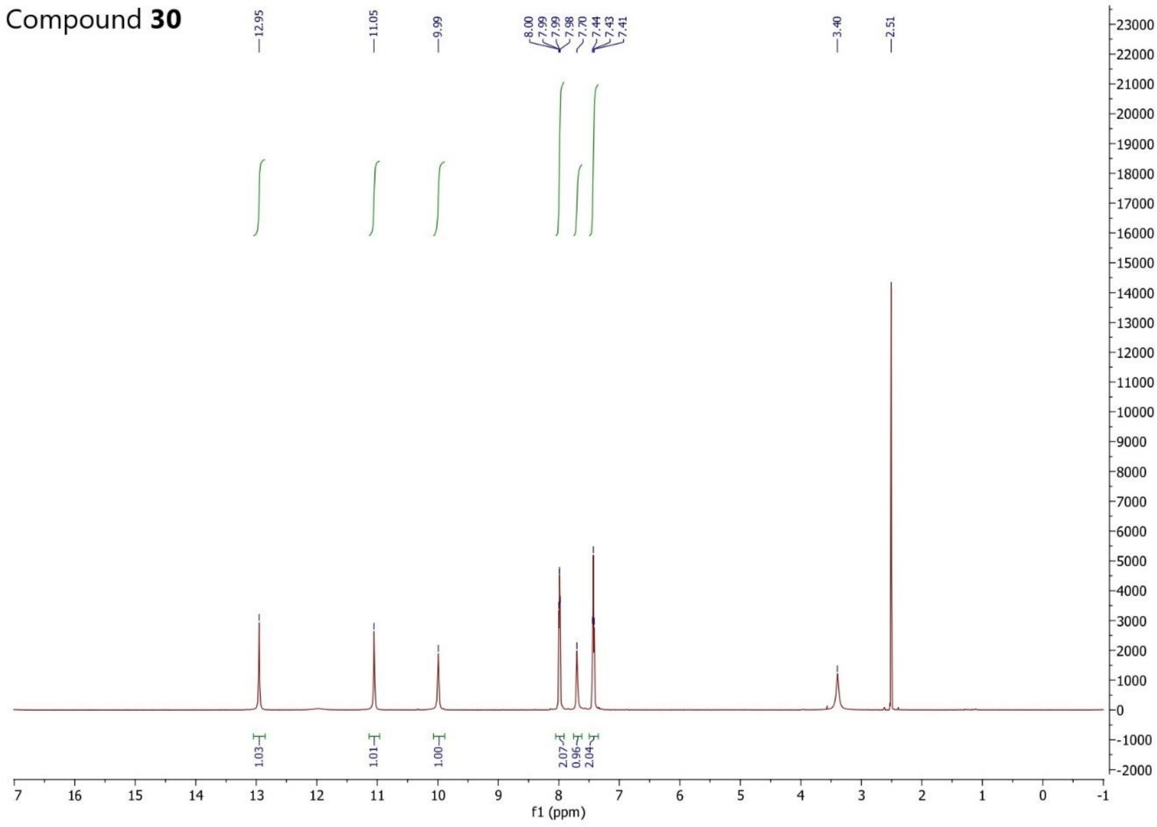

Figure S10. ^{1}H NMR spectrum of compound **30**

Compound **31**

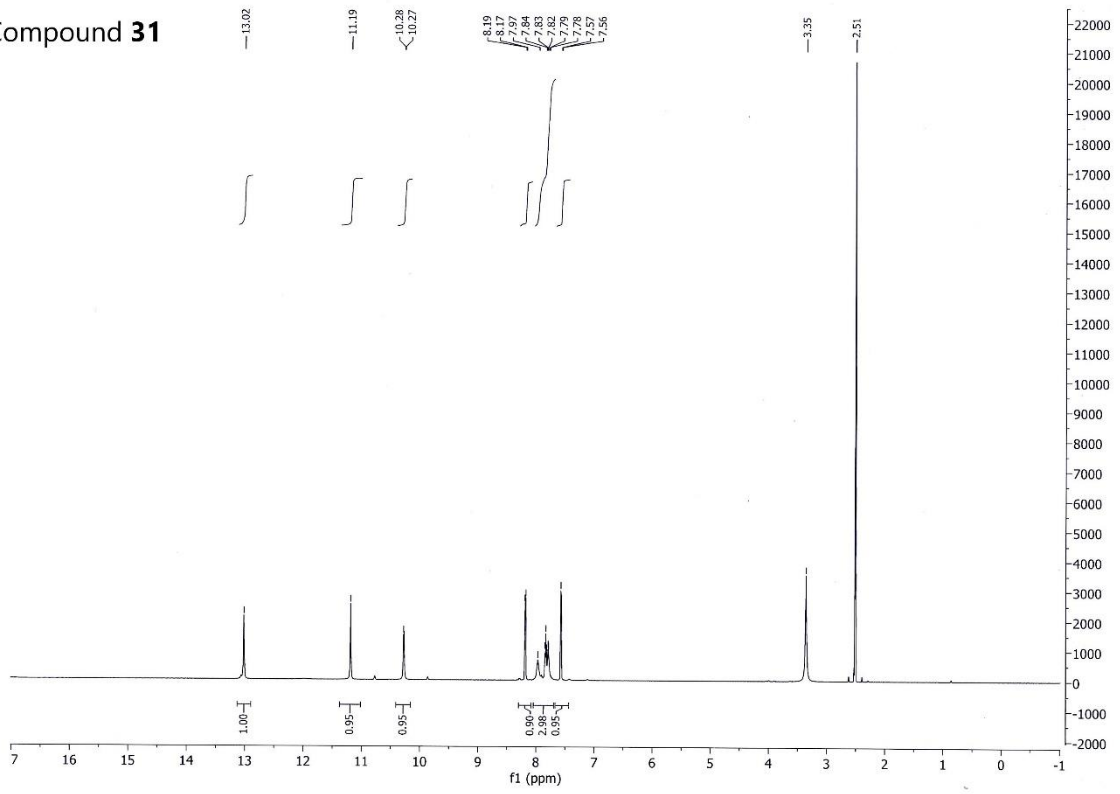

Figure S11. ^{1}H NMR spectrum of compound **31**

Compound **32**

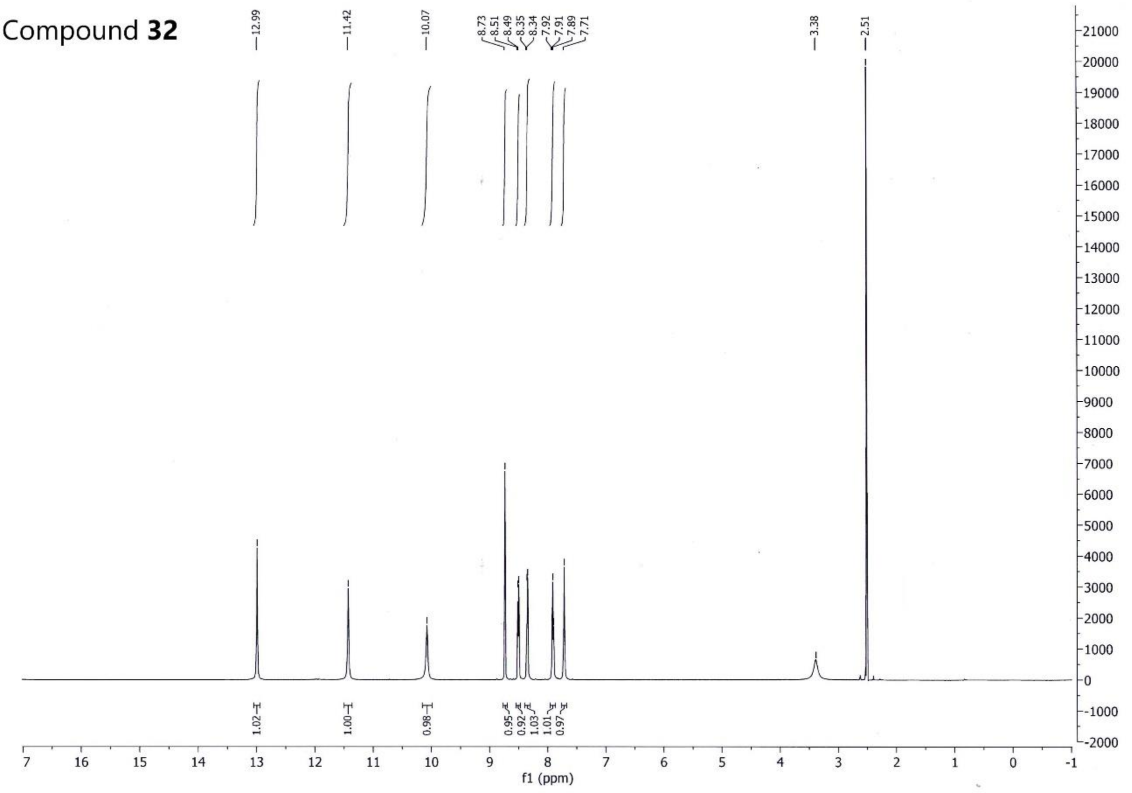

Figure S12. ^{1}H NMR spectrum of compound **32**

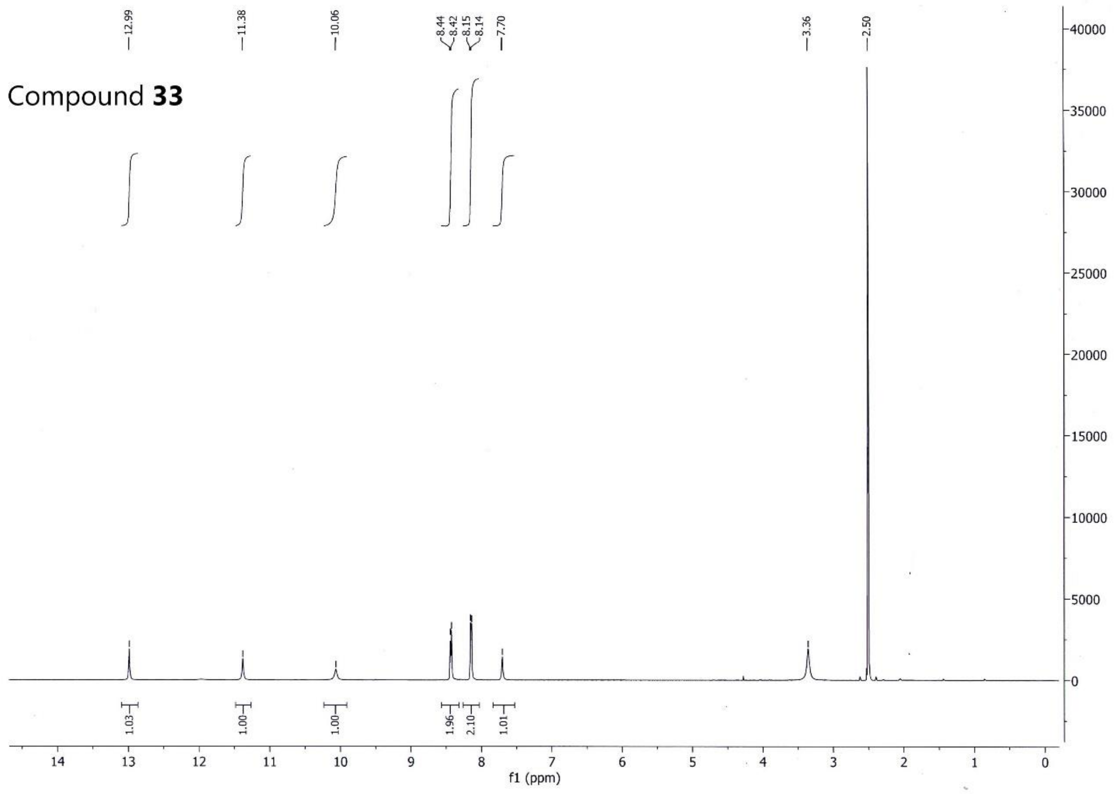

Figure S13. ^{1}H NMR spectrum of compound **33**

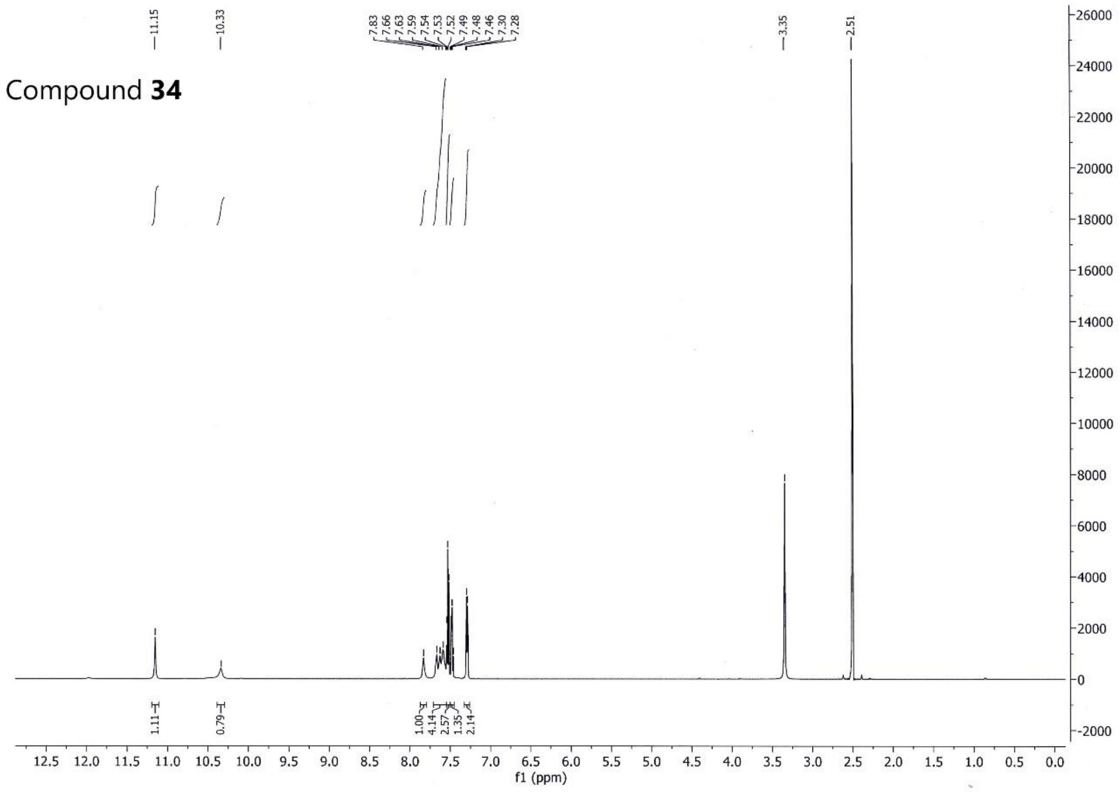

Figure S14. ^{1}H NMR spectrum of compound **34**

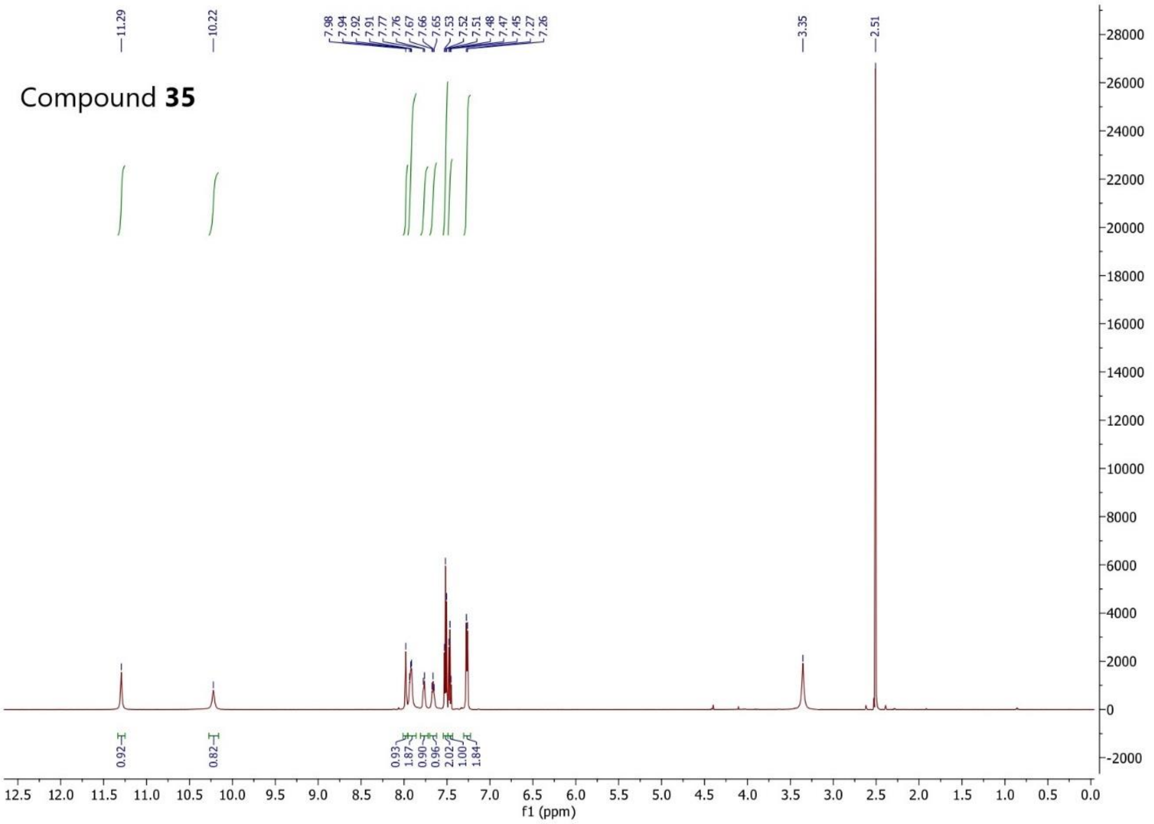

Figure S15. ^{1}H NMR spectrum of compound **35**

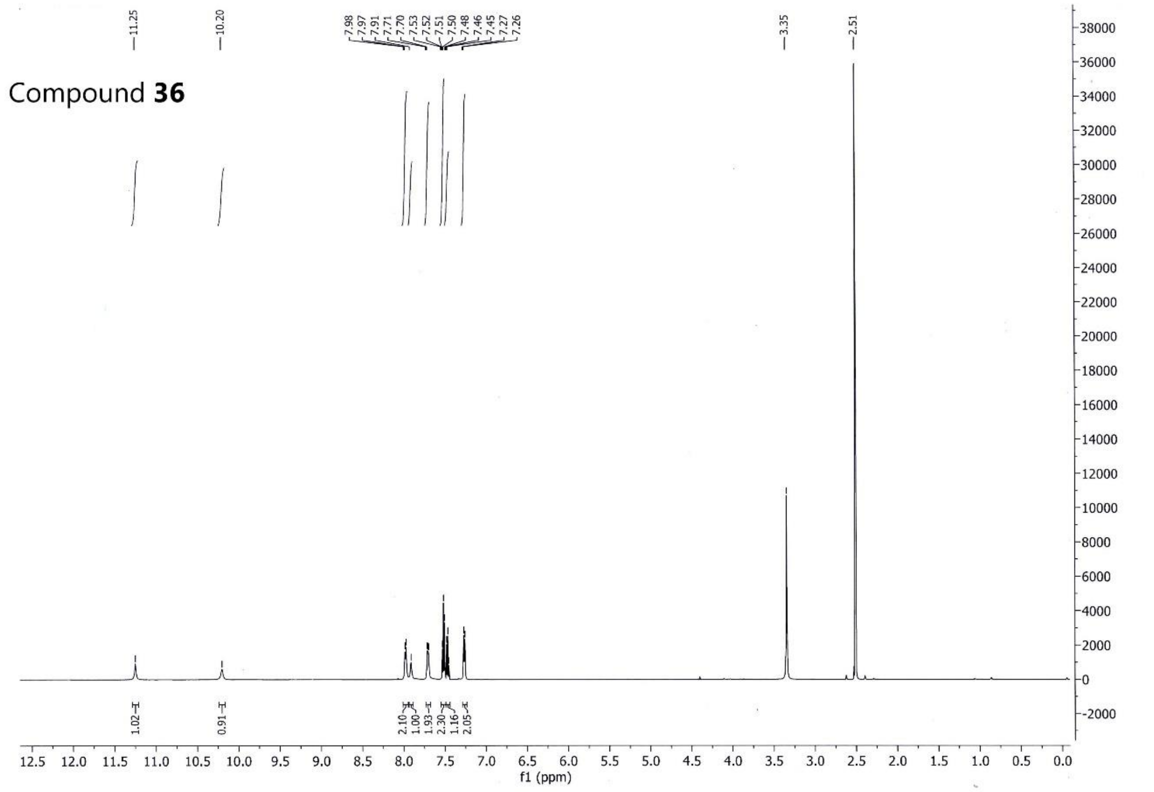

Figure S16. ^{1}H NMR spectrum of compound **36**

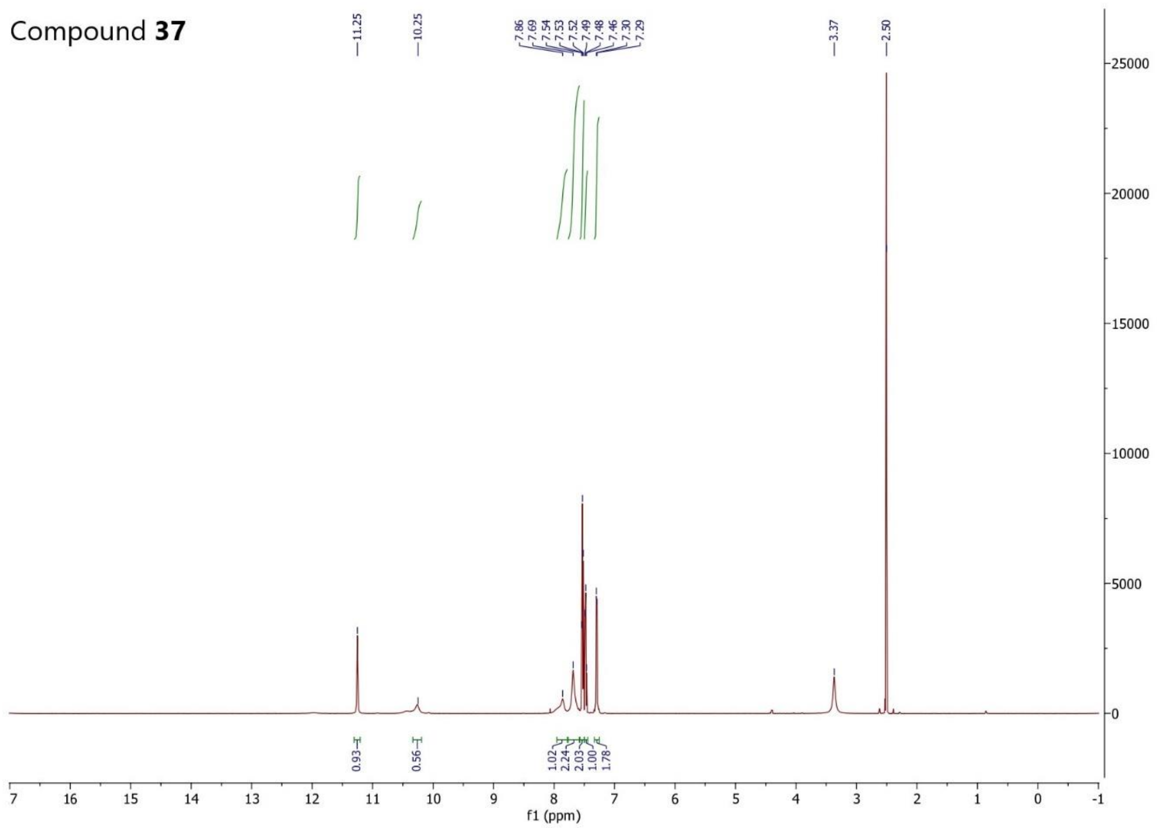

Figure S17. ^{1}H NMR spectrum of compound **37**

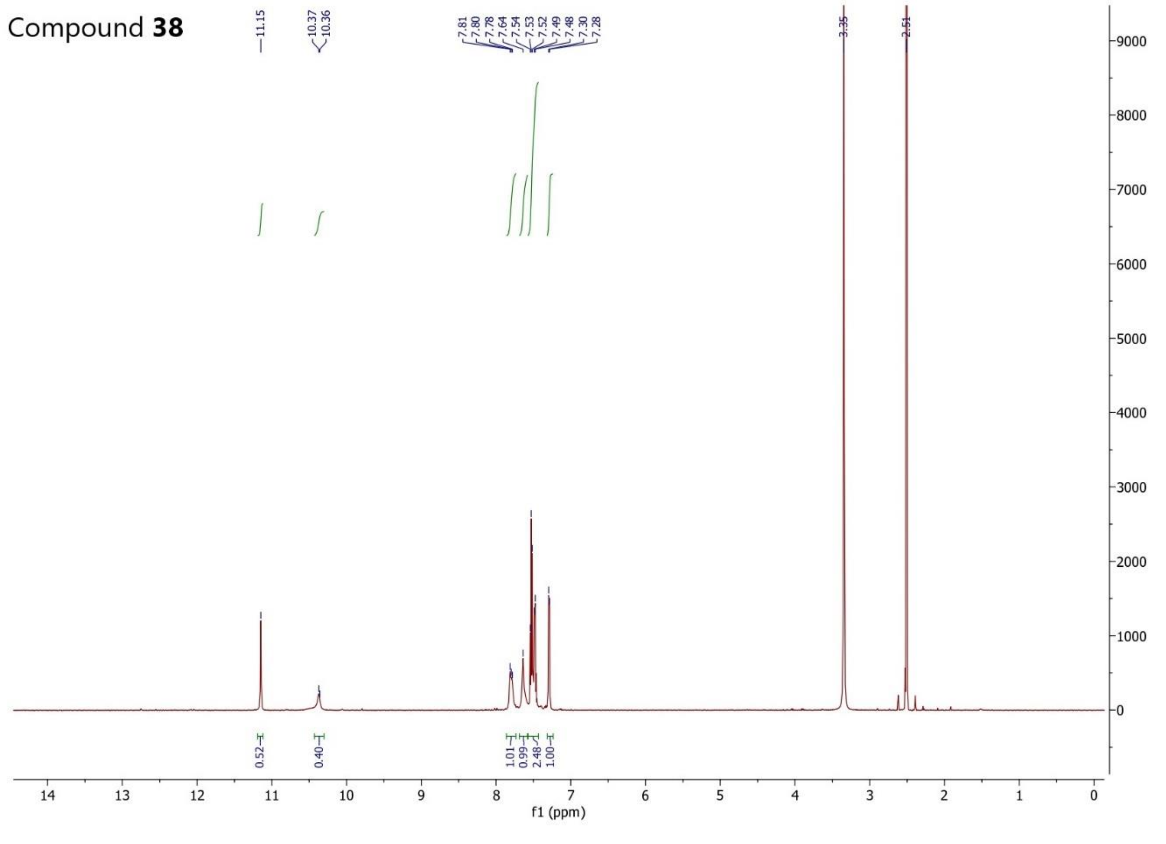

Figure S18. ^{1}H NMR spectrum of compound **38**

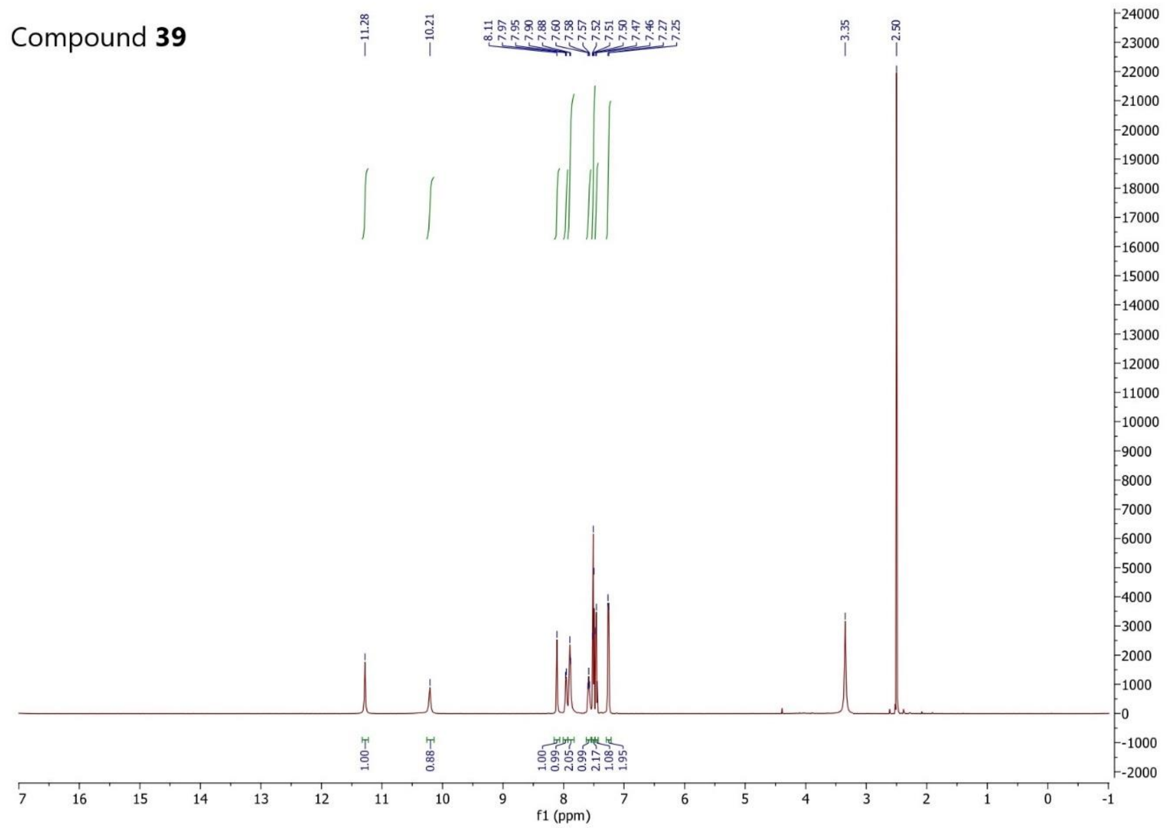

Figure S19. ^{1}H NMR spectrum of compound **39**

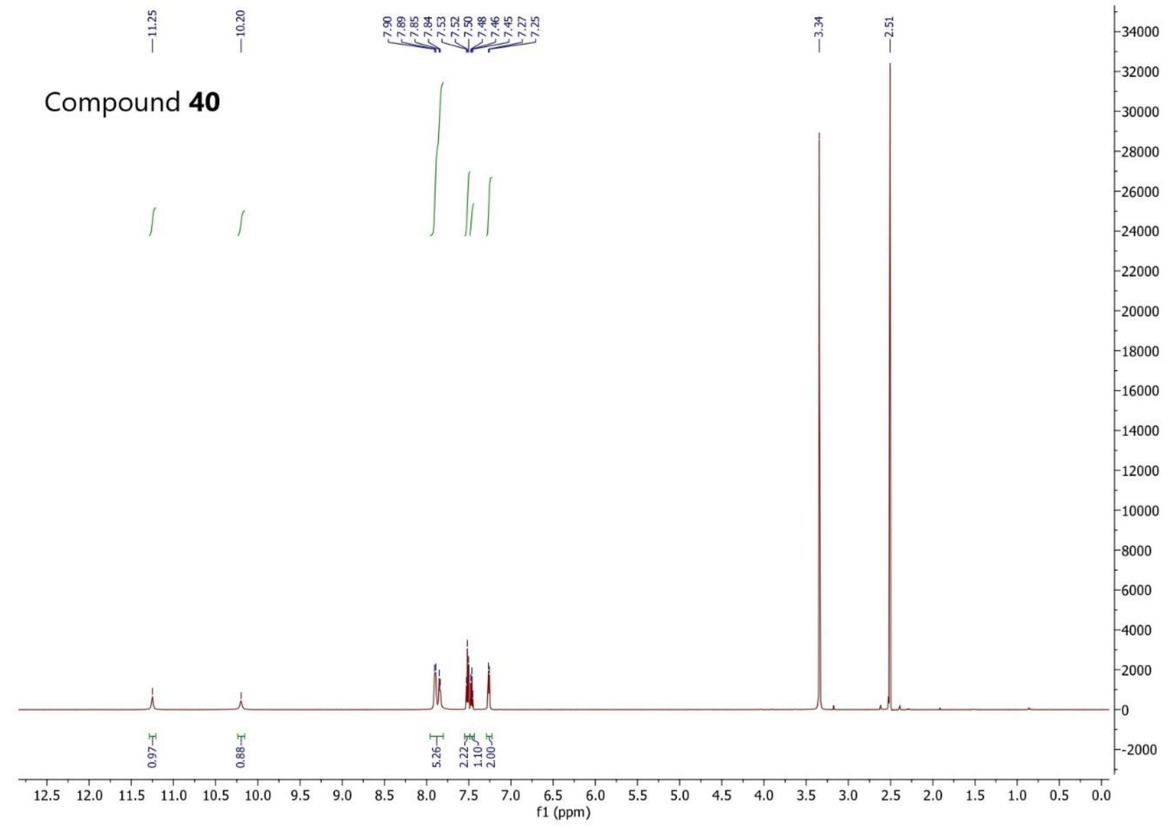

Figure S20. ^{1}H NMR spectrum of compound **40**

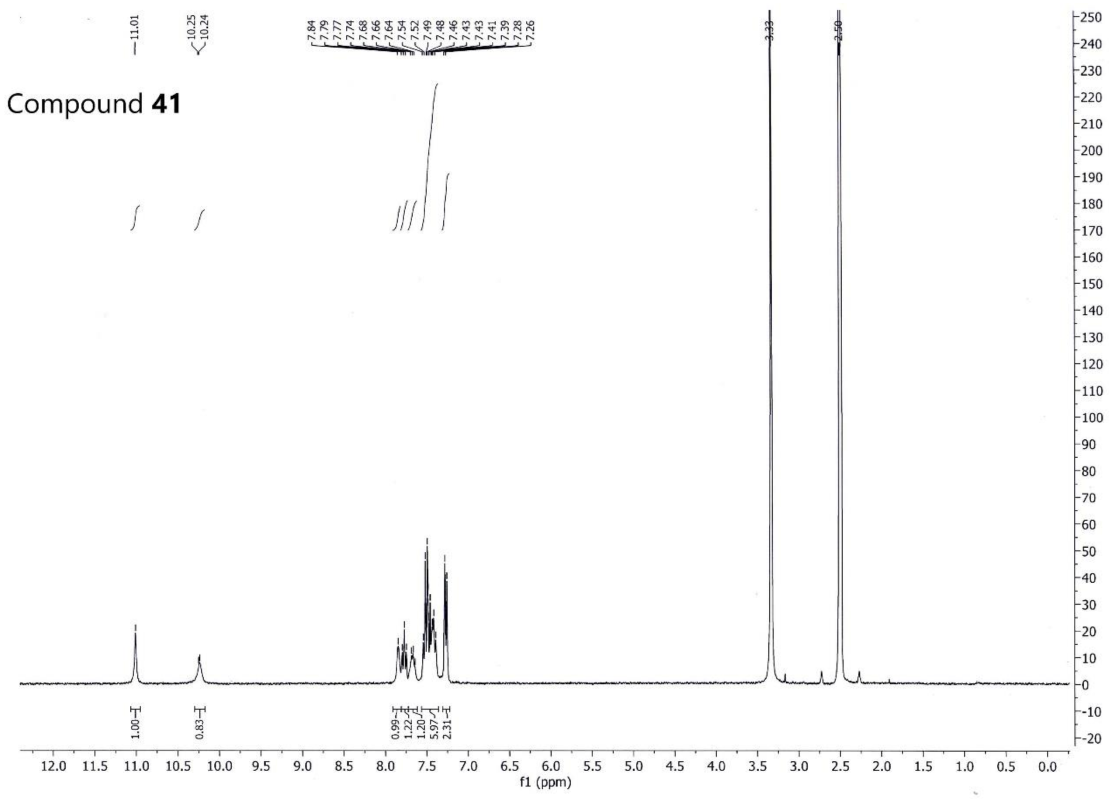

Figure S21. ^{1}H NMR spectrum of compound **41**

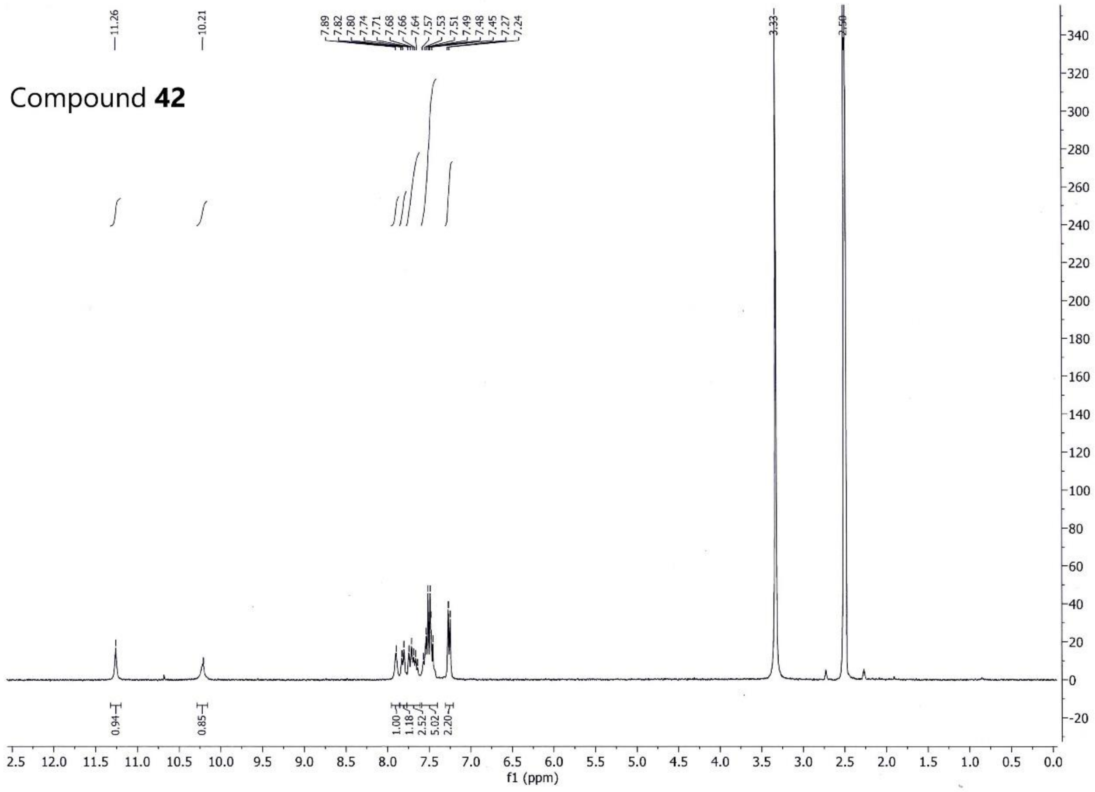

Figure S22. ^{1}H NMR spectrum of compound **42**

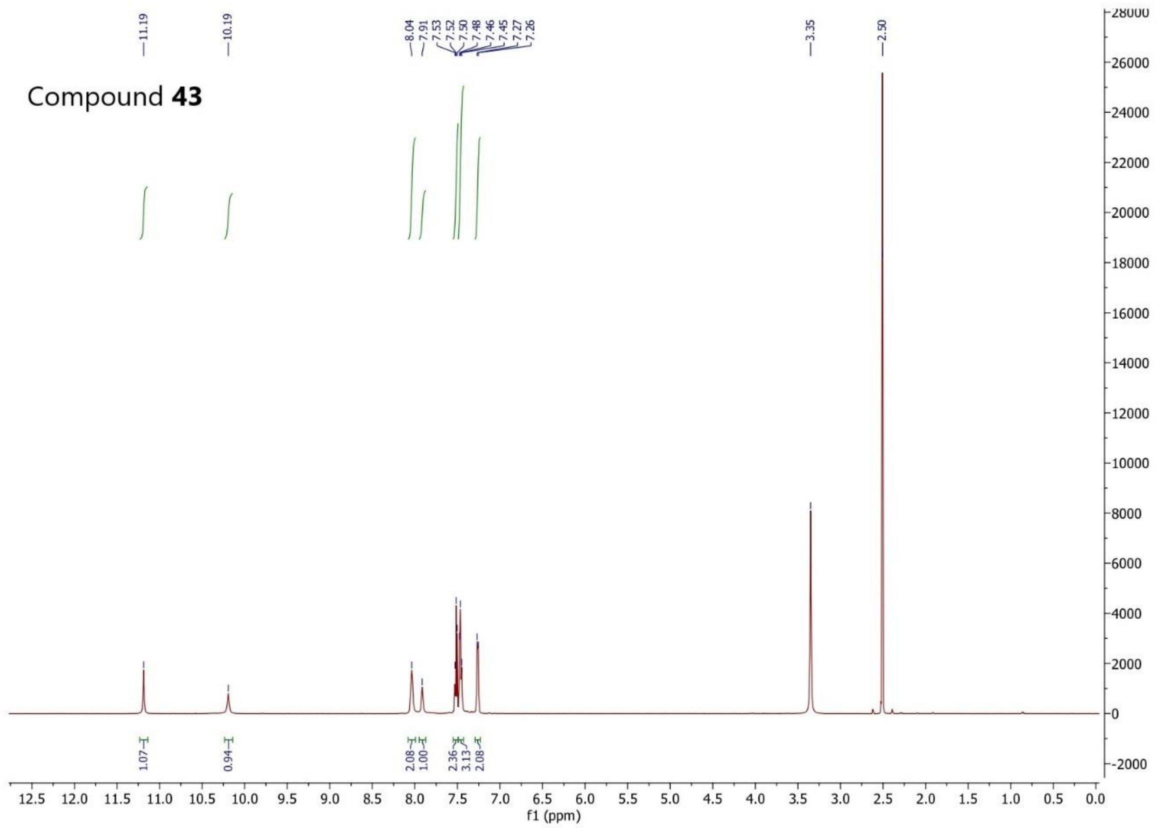

Figure S23. ^{1}H NMR spectrum of compound **43**

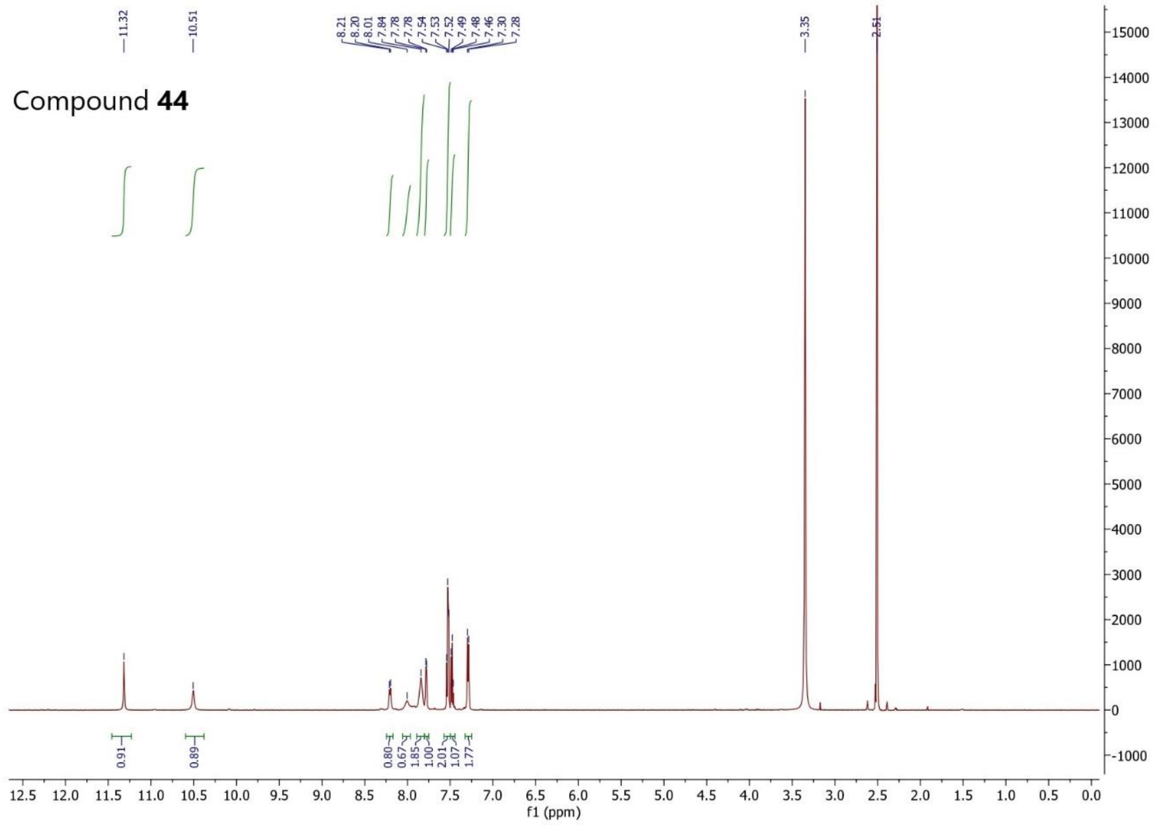

Figure S24. ^{1}H NMR spectrum of compound **44**

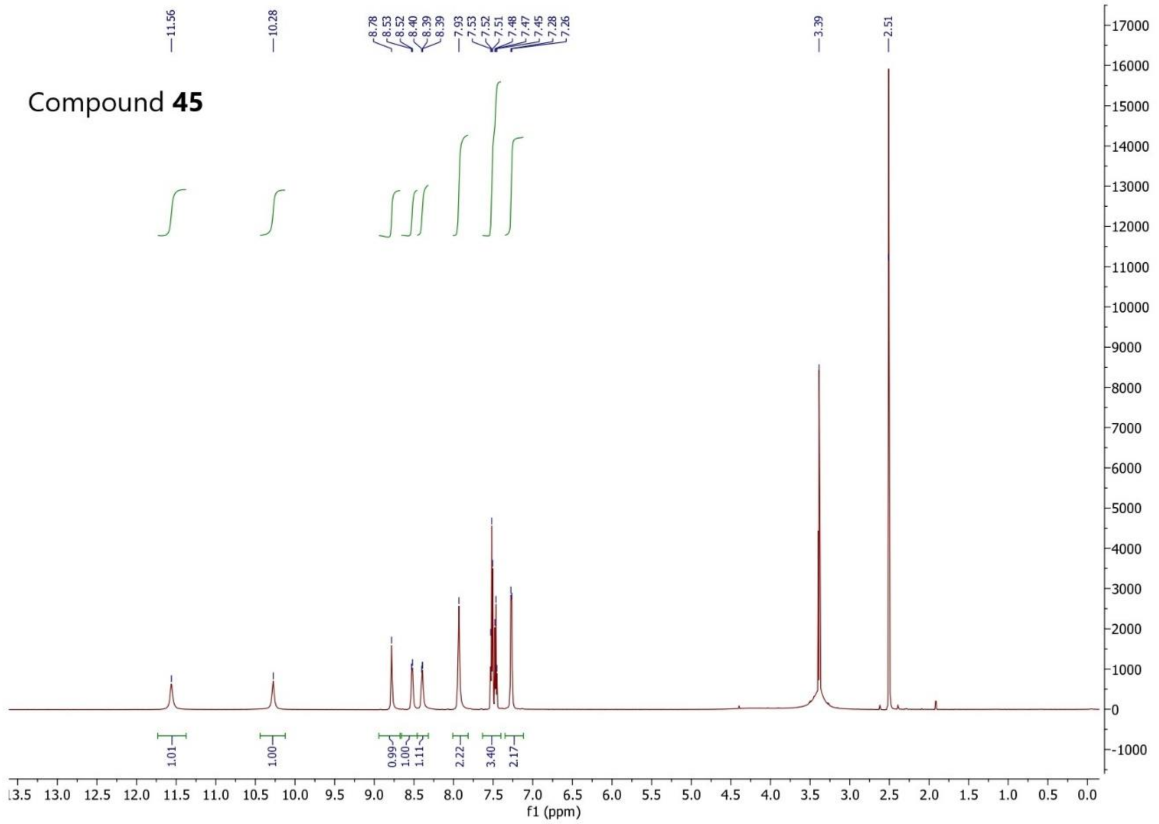

Figure S25. ^{1}H NMR spectrum of compound **45**

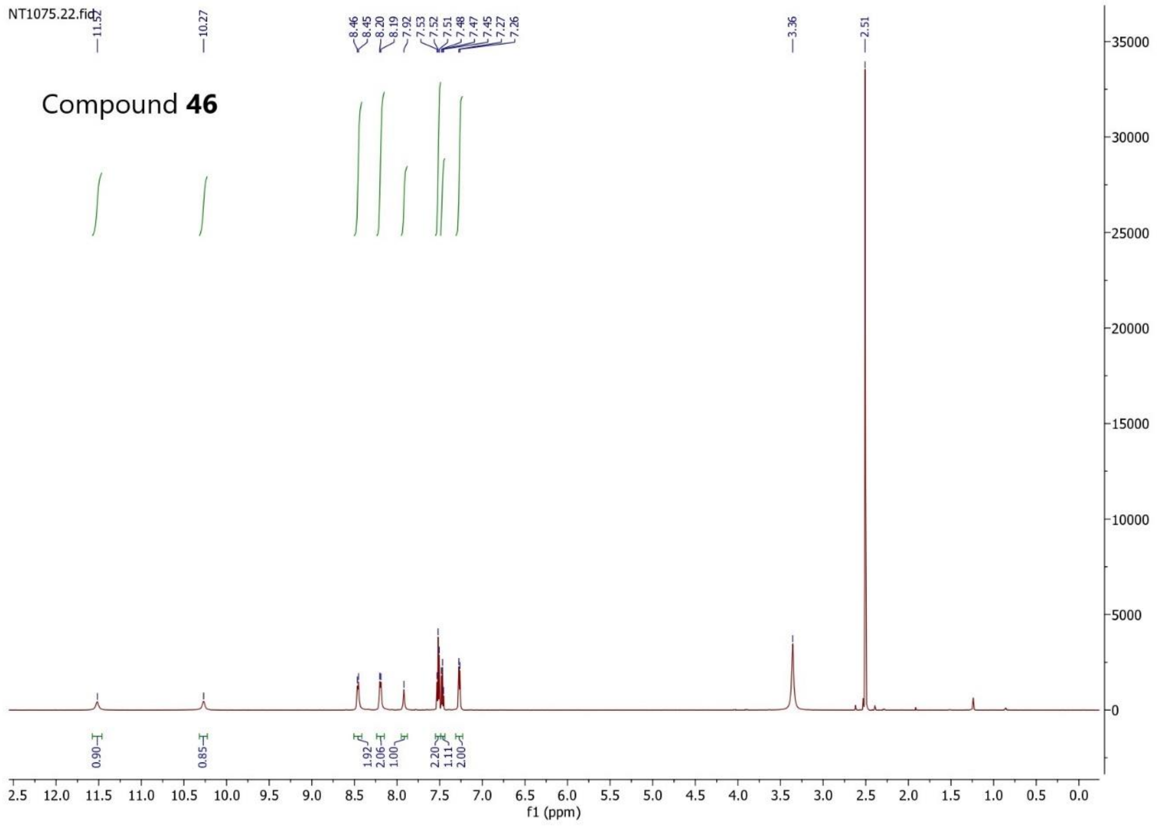

Figure S26. ^{1}H NMR spectrum of compound **46**

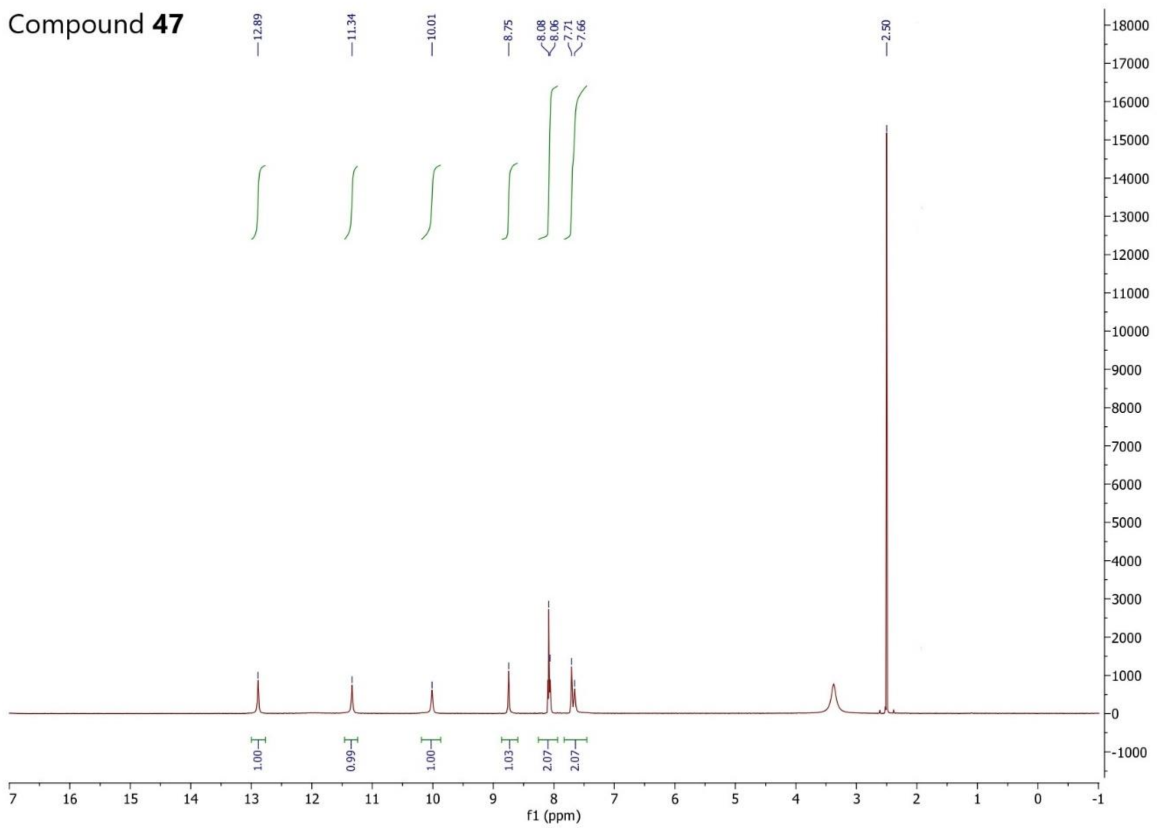

Figure S27. ^{1}H NMR spectrum of compound **47**

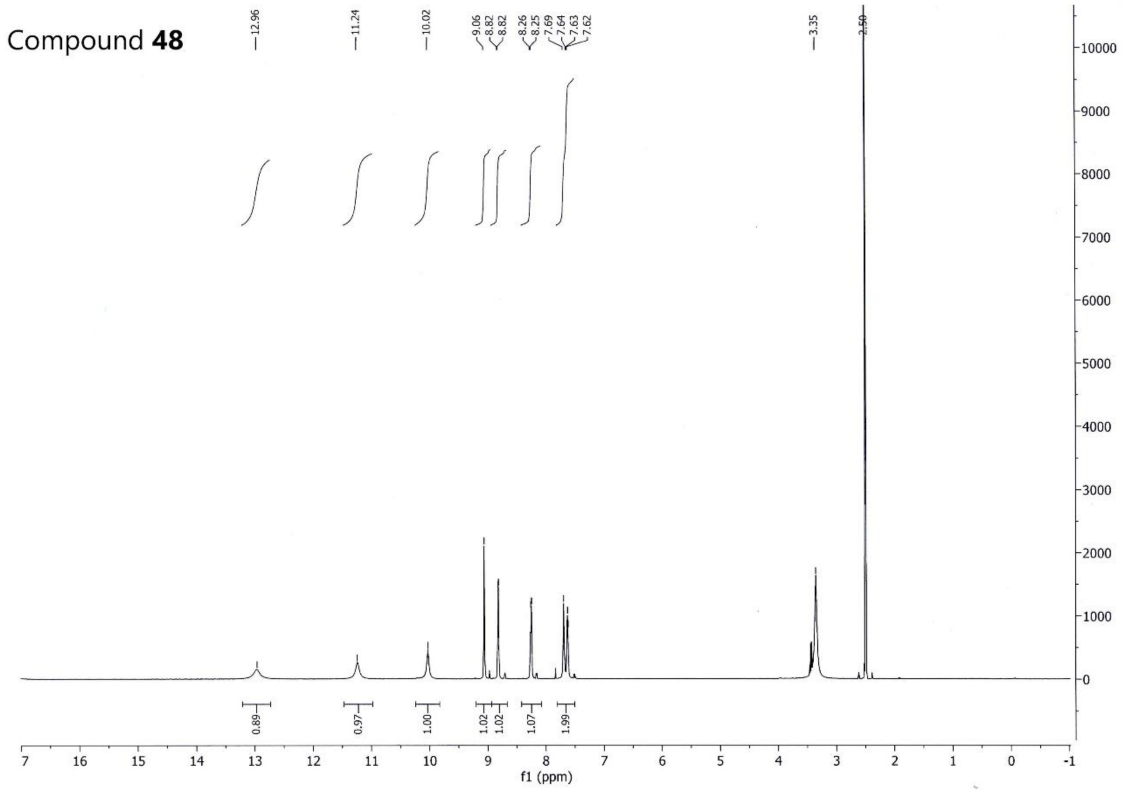

Figure S28. ^{1}H NMR spectrum of compound **48**

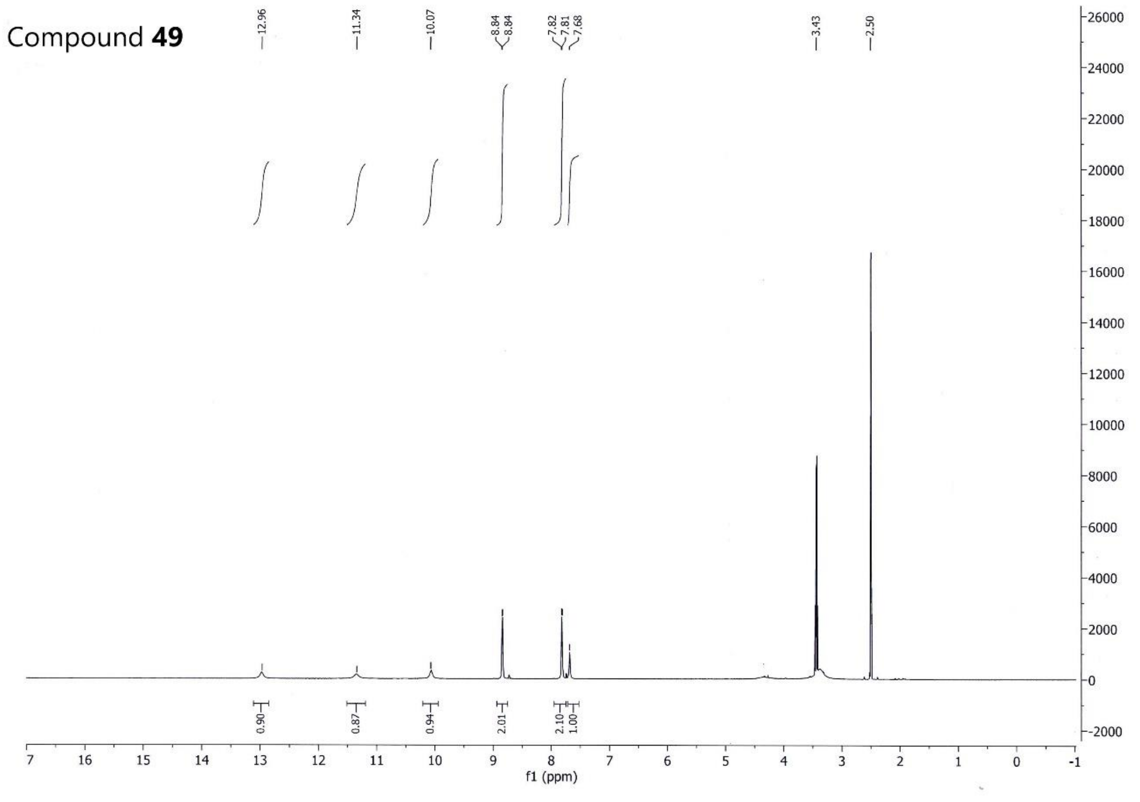

Figure S29. ^{1}H NMR spectrum of compound **49**

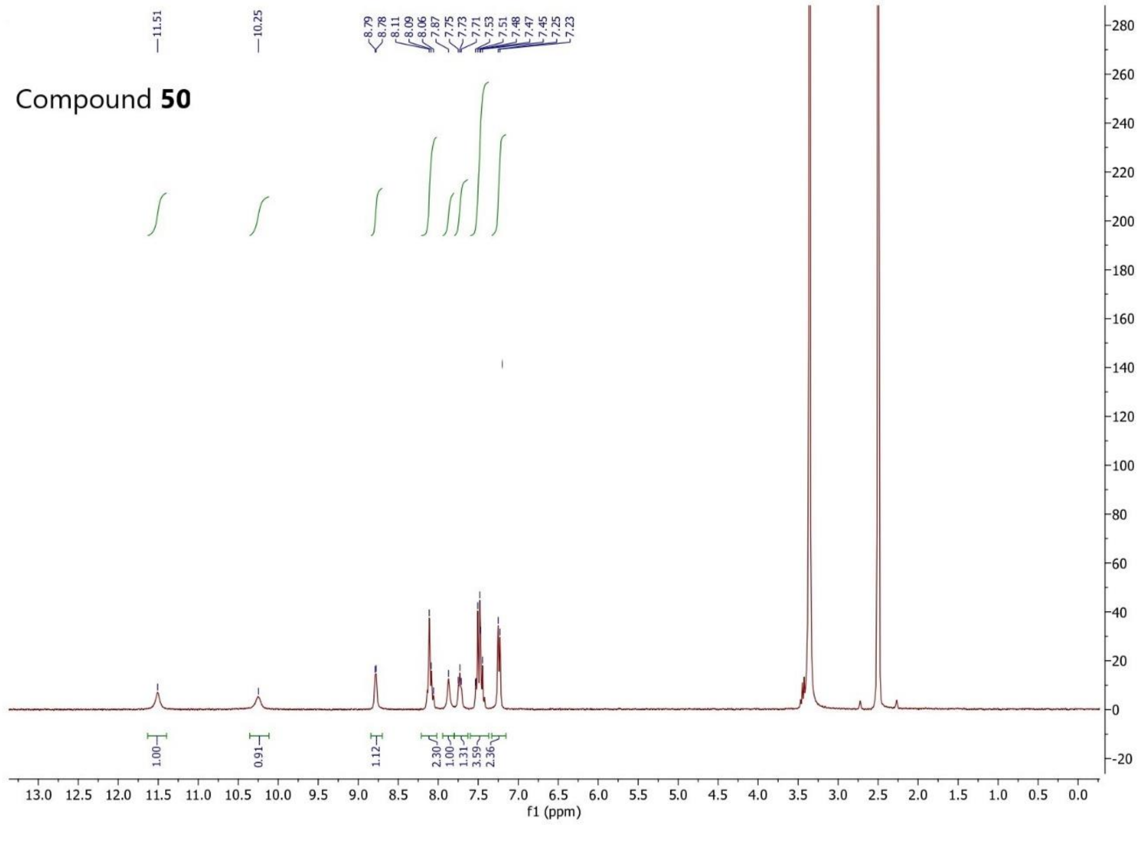

Figure S30. ^{1}H NMR spectrum of compound **50**

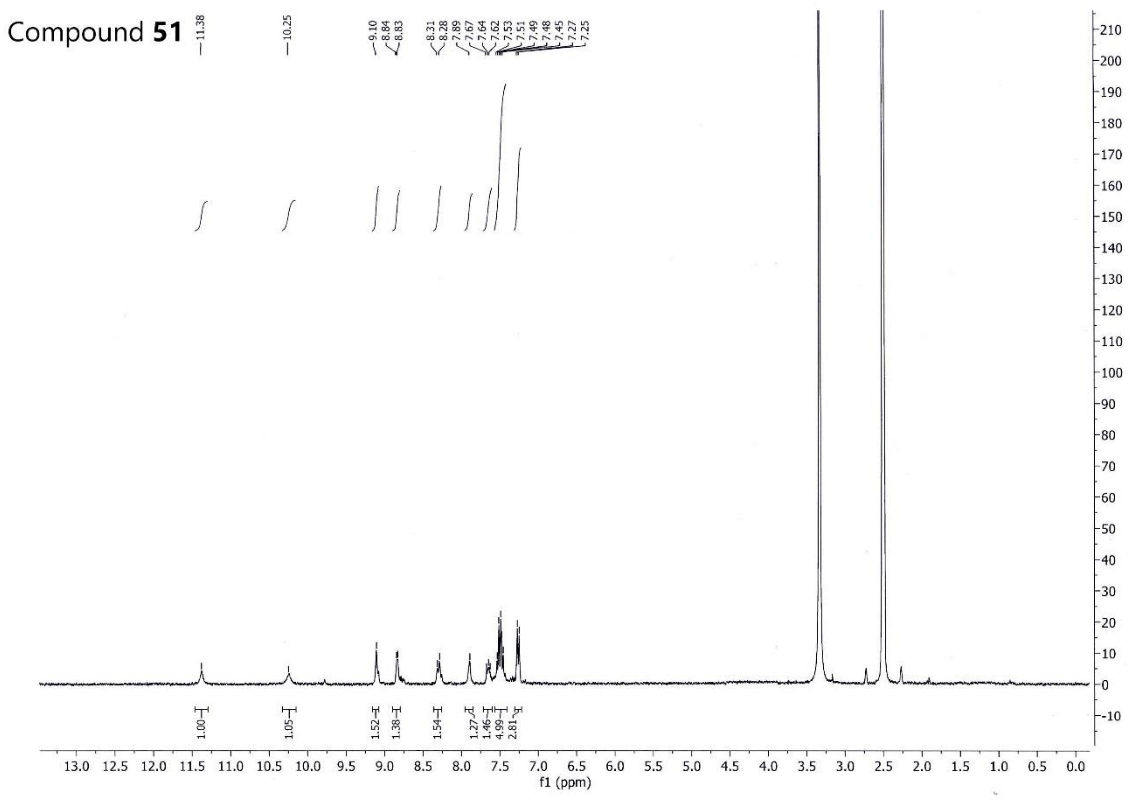

Figure S31. ^{1}H NMR spectrum of compound **51**

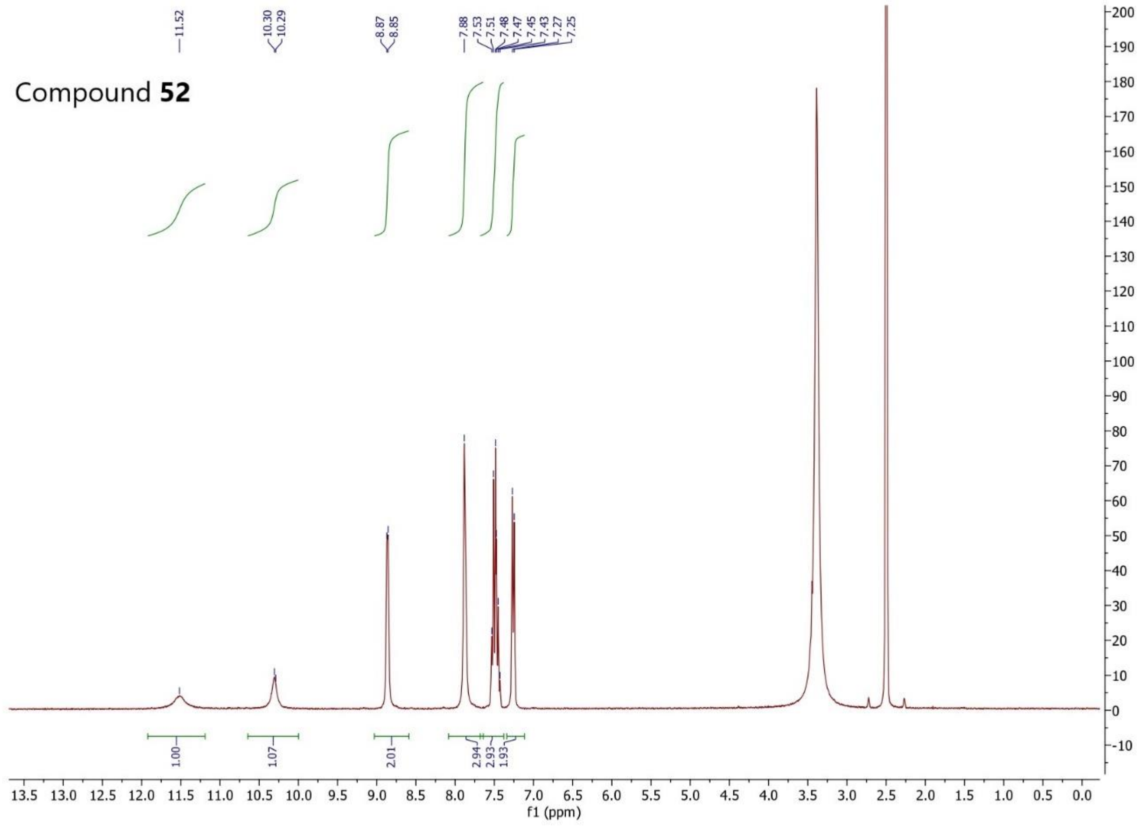

Figure S32. ^{1}H NMR spectrum of compound **52**

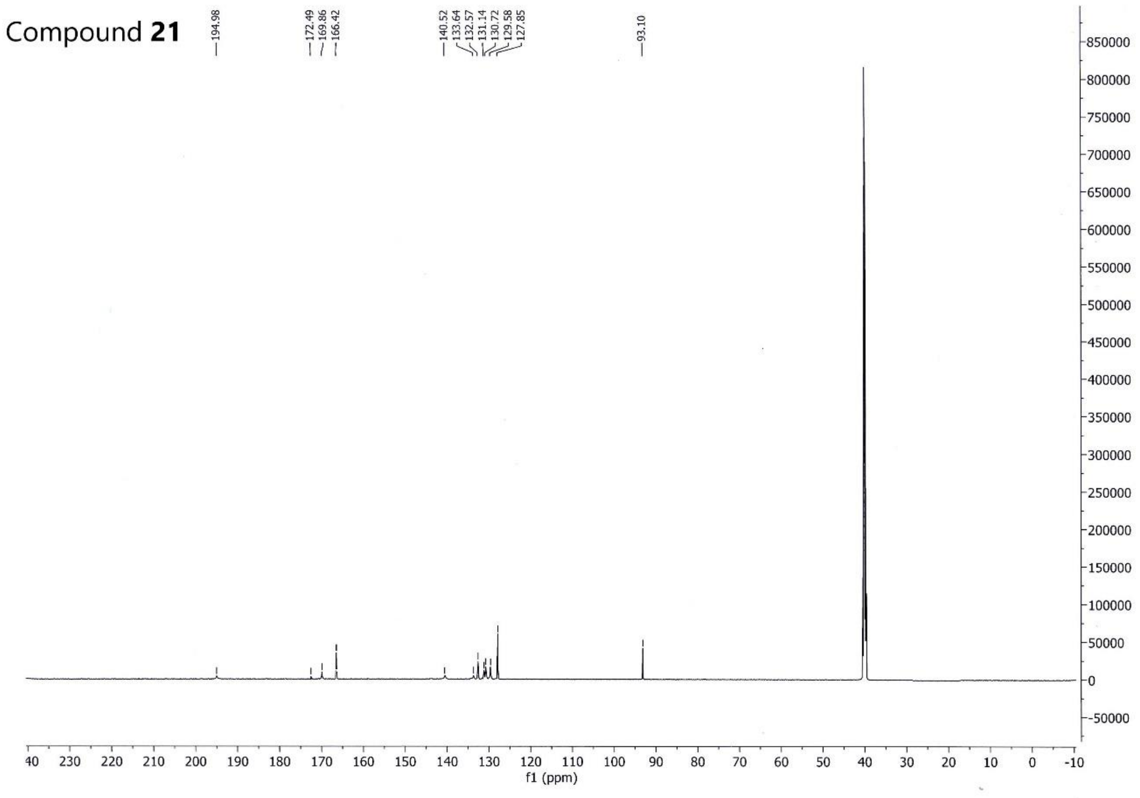

Figure S33. ^{13}C NMR spectrum of compound **21**

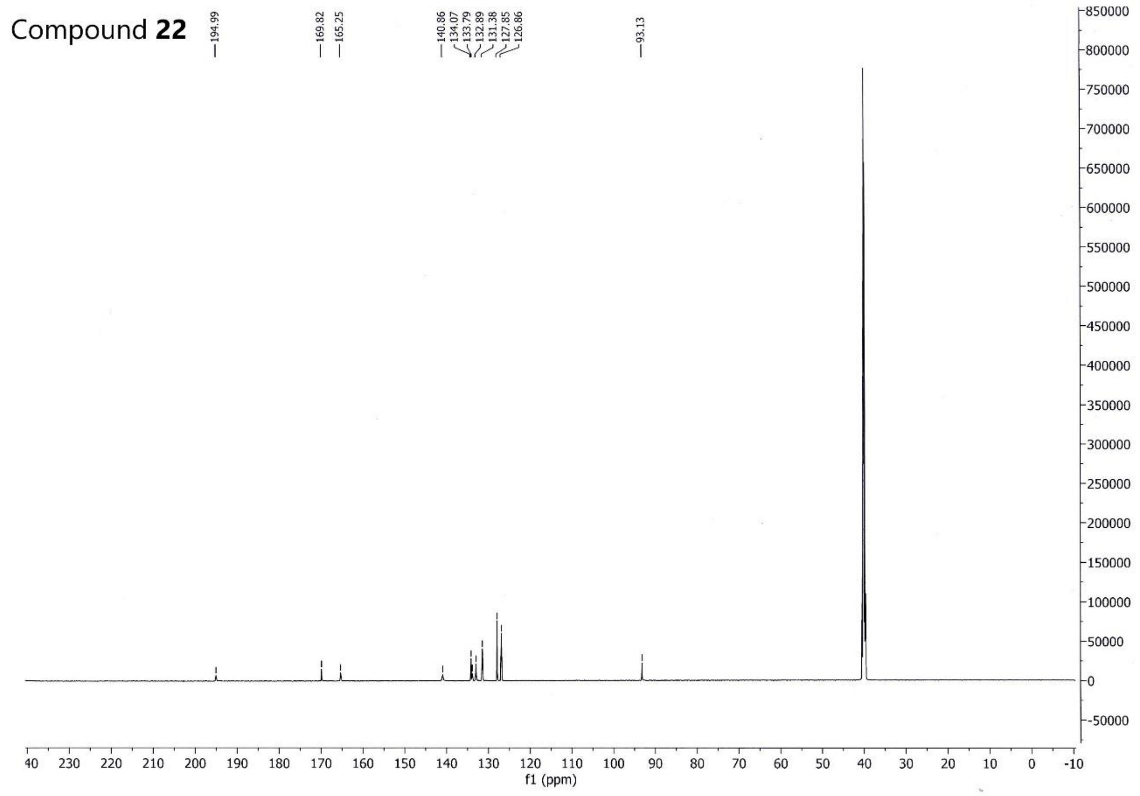

Figure S34. ^{13}C NMR spectrum of compound **22**

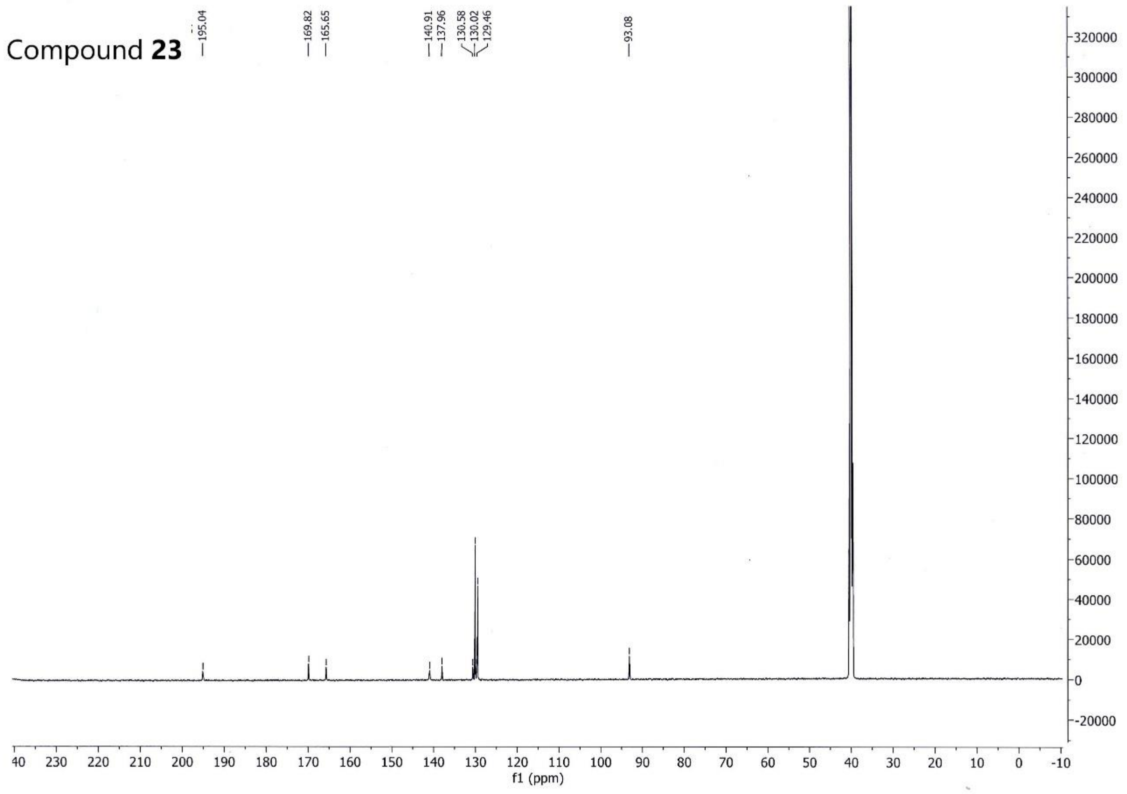

Figure S35. ^{13}C NMR spectrum of compound **23**

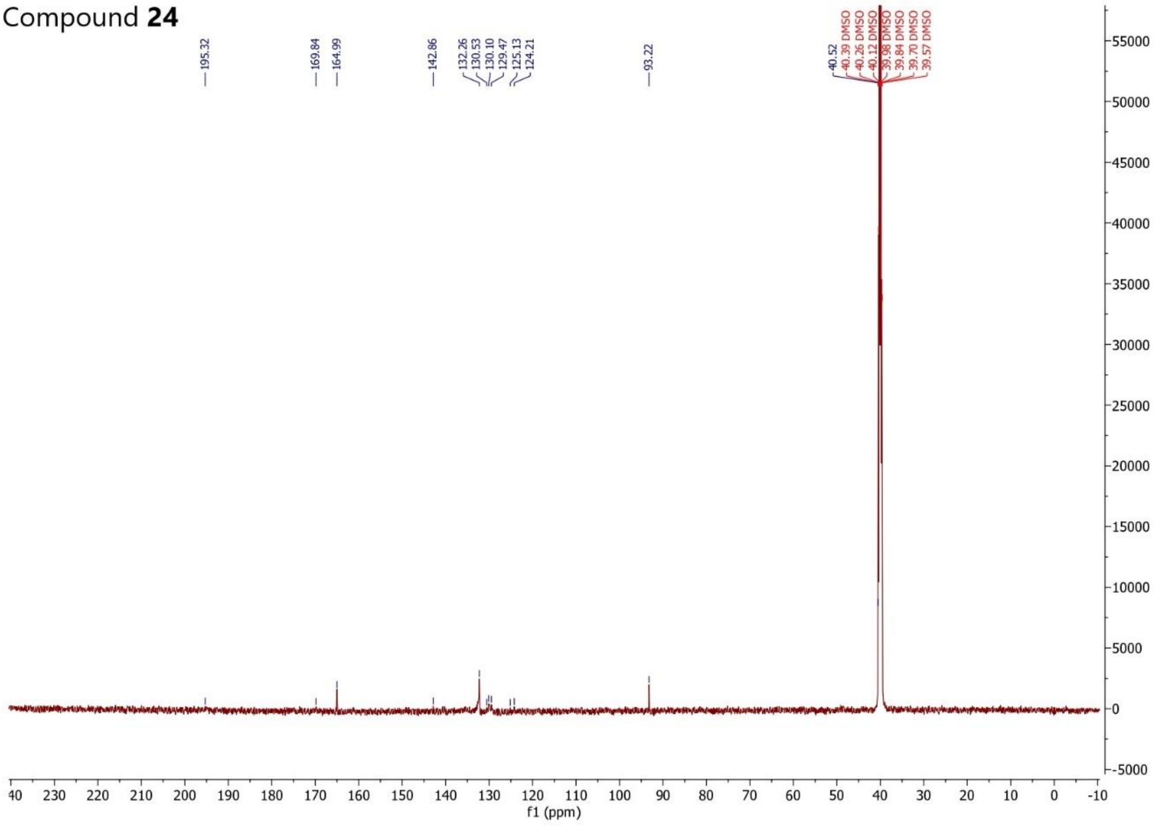

Figure S36. ^{13}C NMR spectrum of compound **24**

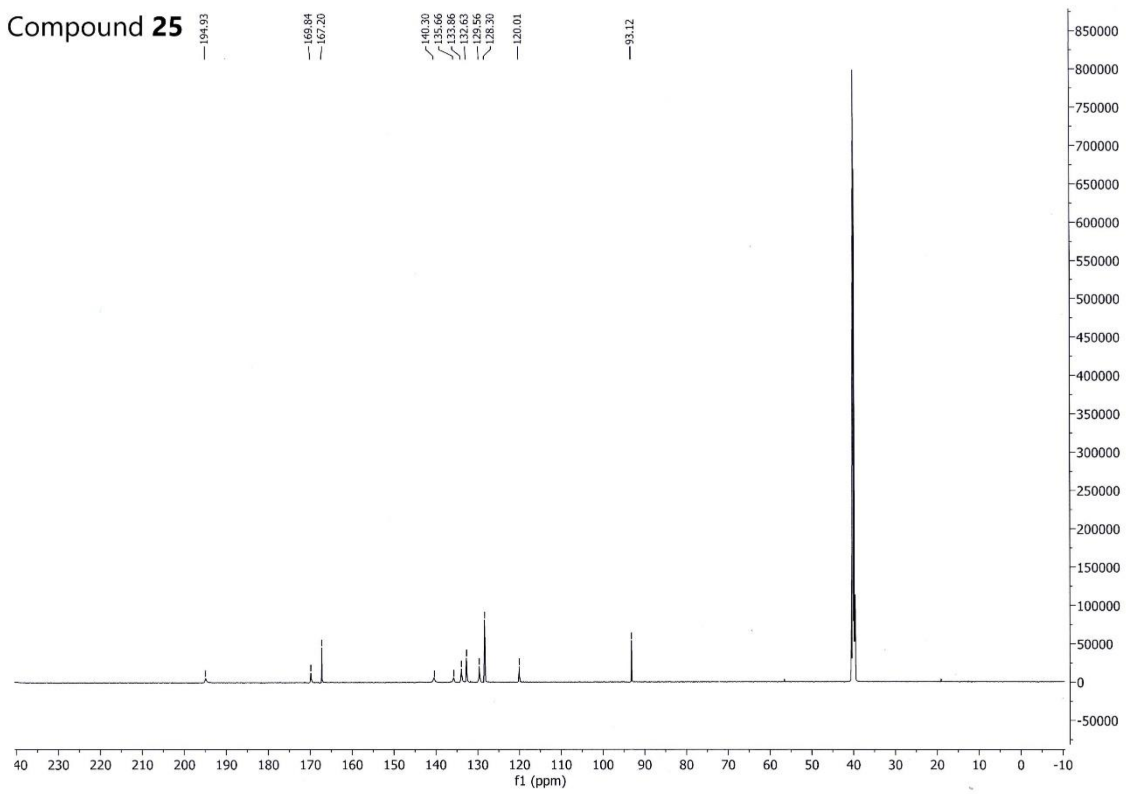

Figure S37. ^{13}C NMR spectrum of compound **25**

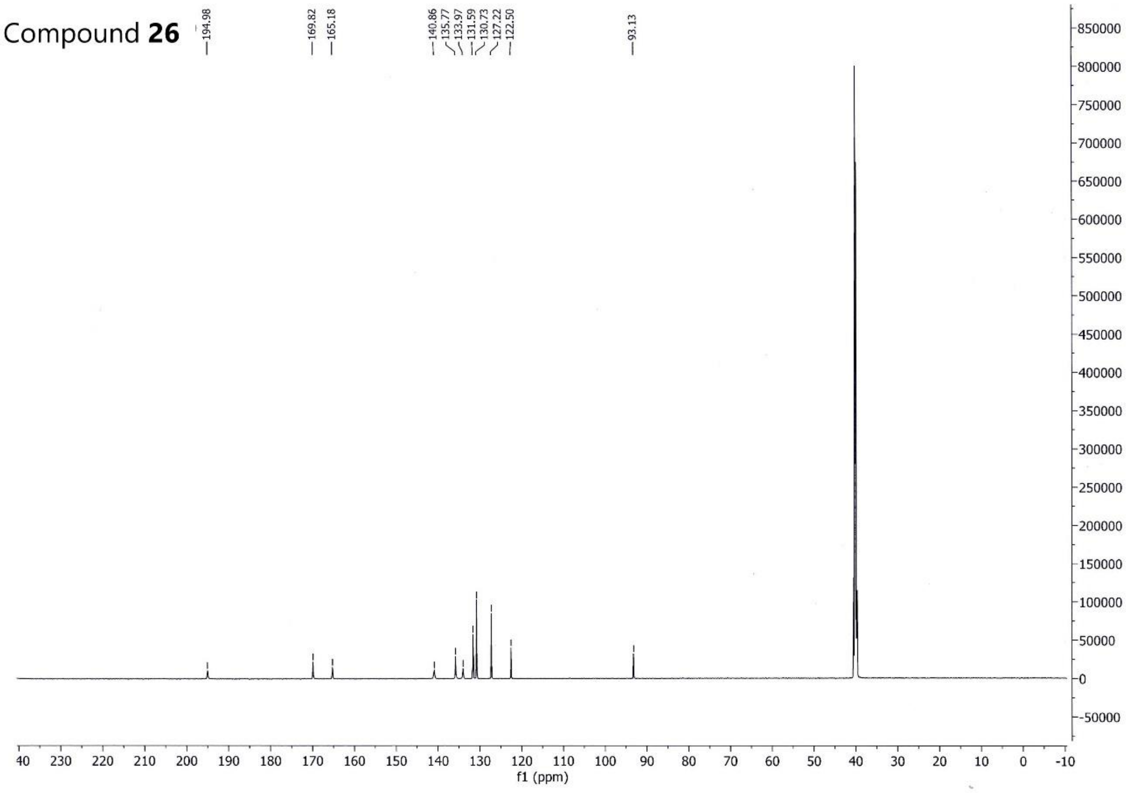

Figure S38. ^{13}C NMR spectrum of compound **26**

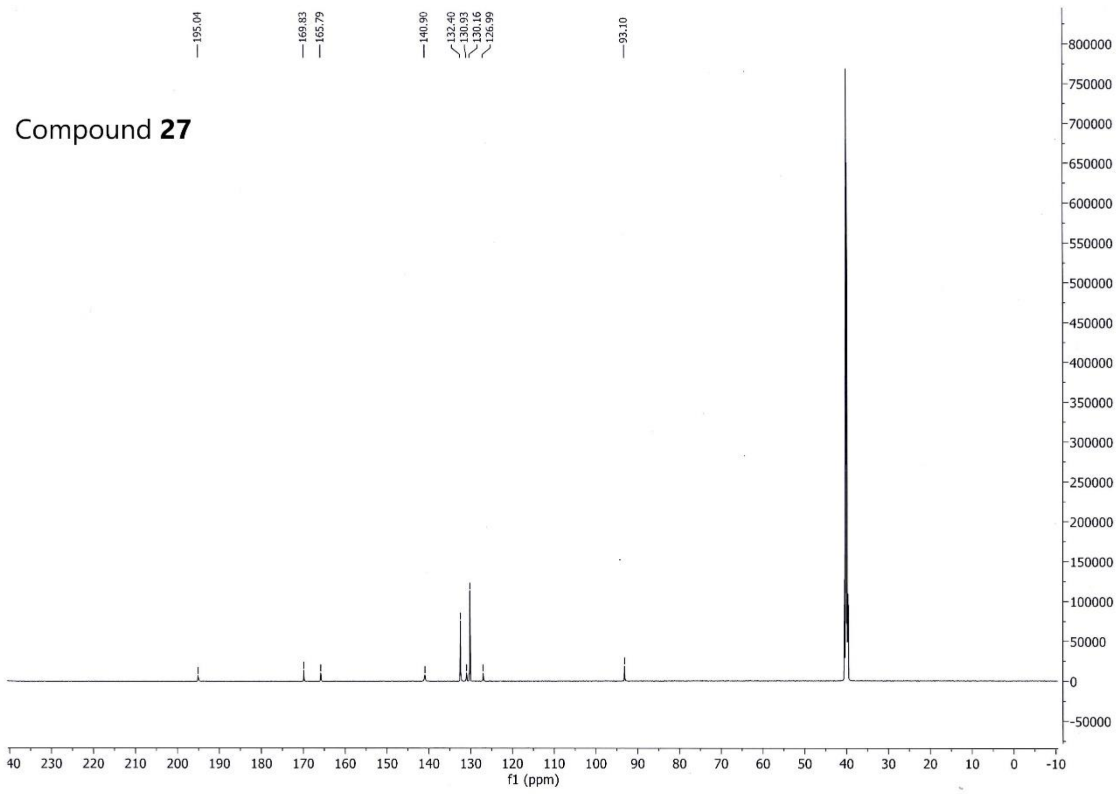

Figure S39. ^{13}C NMR spectrum of compound **27**

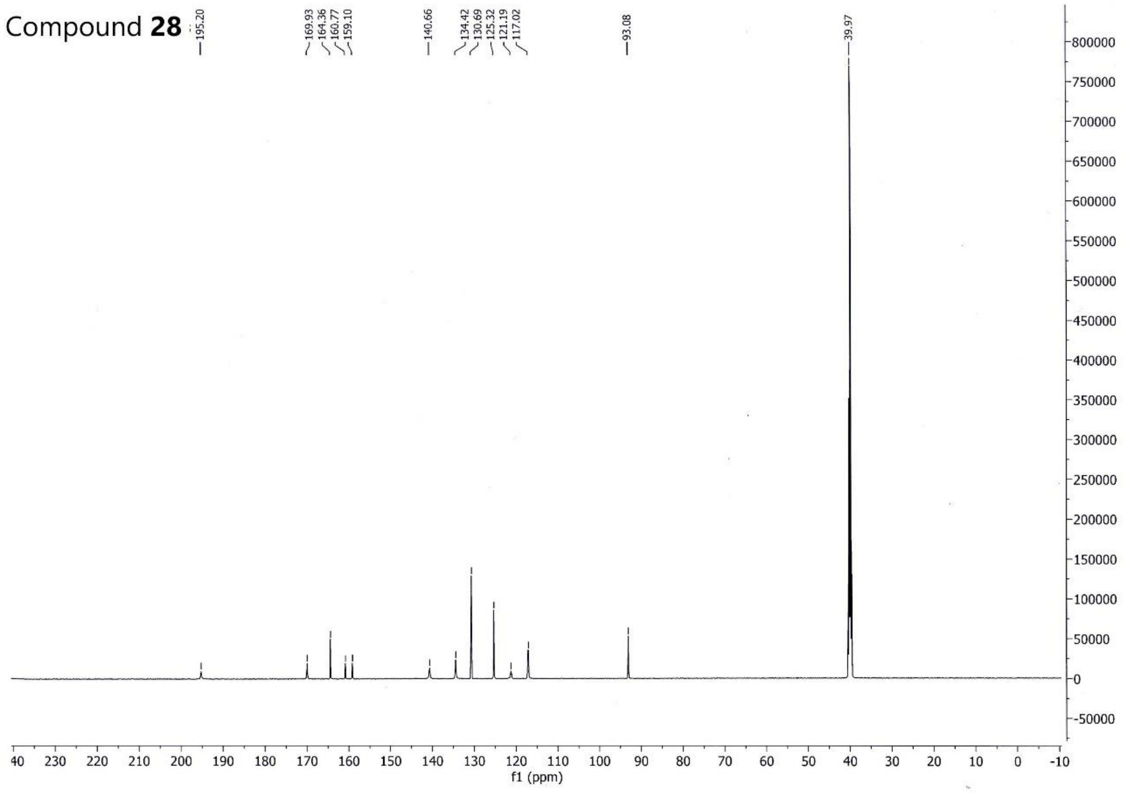

Figure S40. ^{13}C NMR spectrum of compound **28**

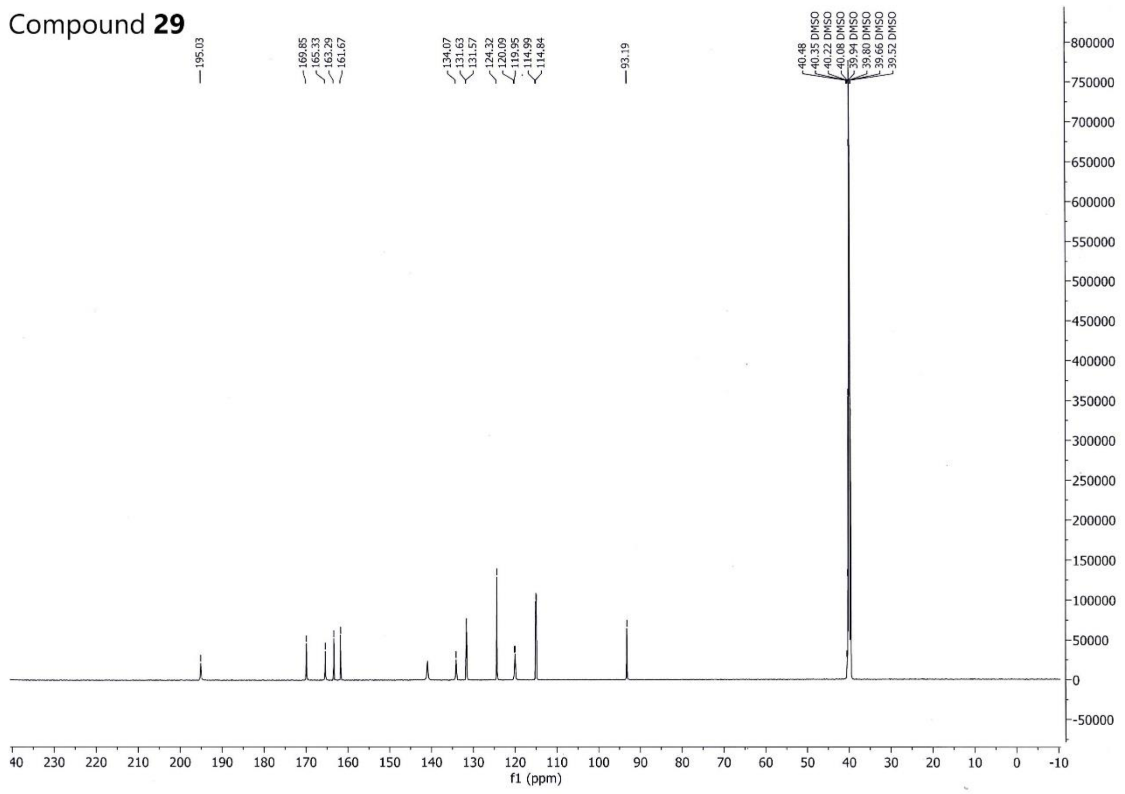

Figure S41. ^{13}C NMR spectrum of compound **29**

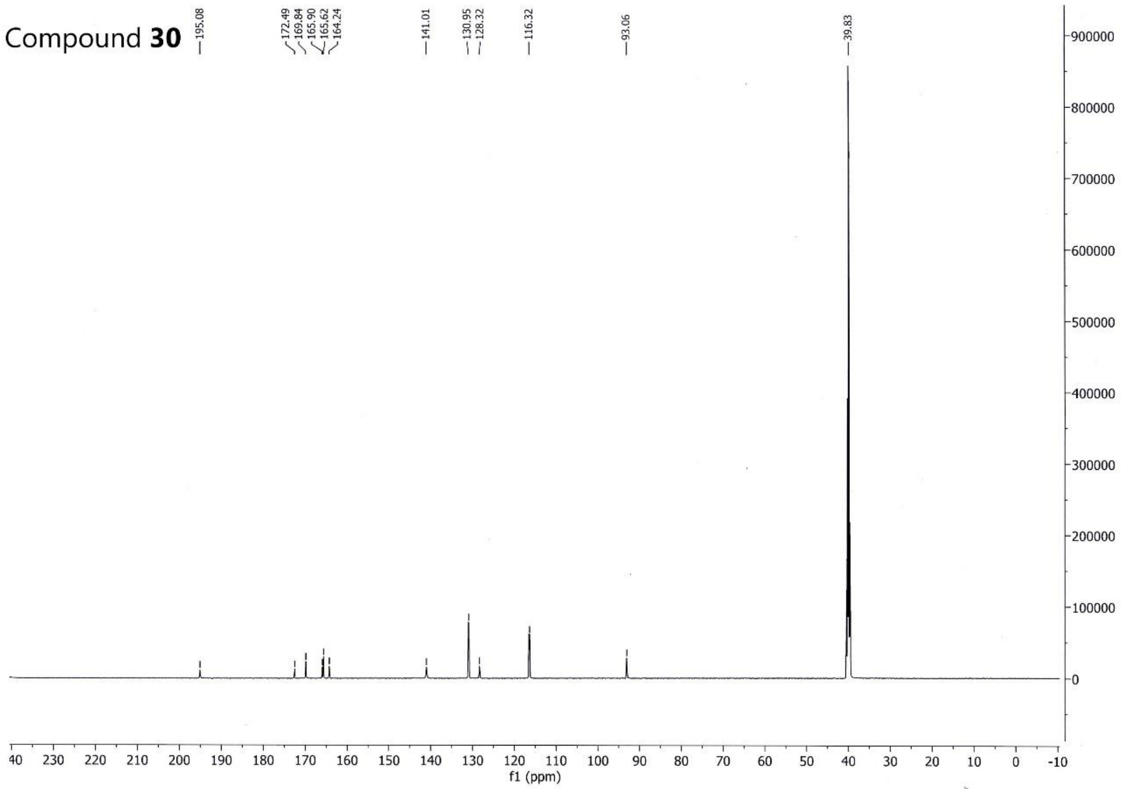

Figure S42. ^{13}C NMR spectrum of compound **30**

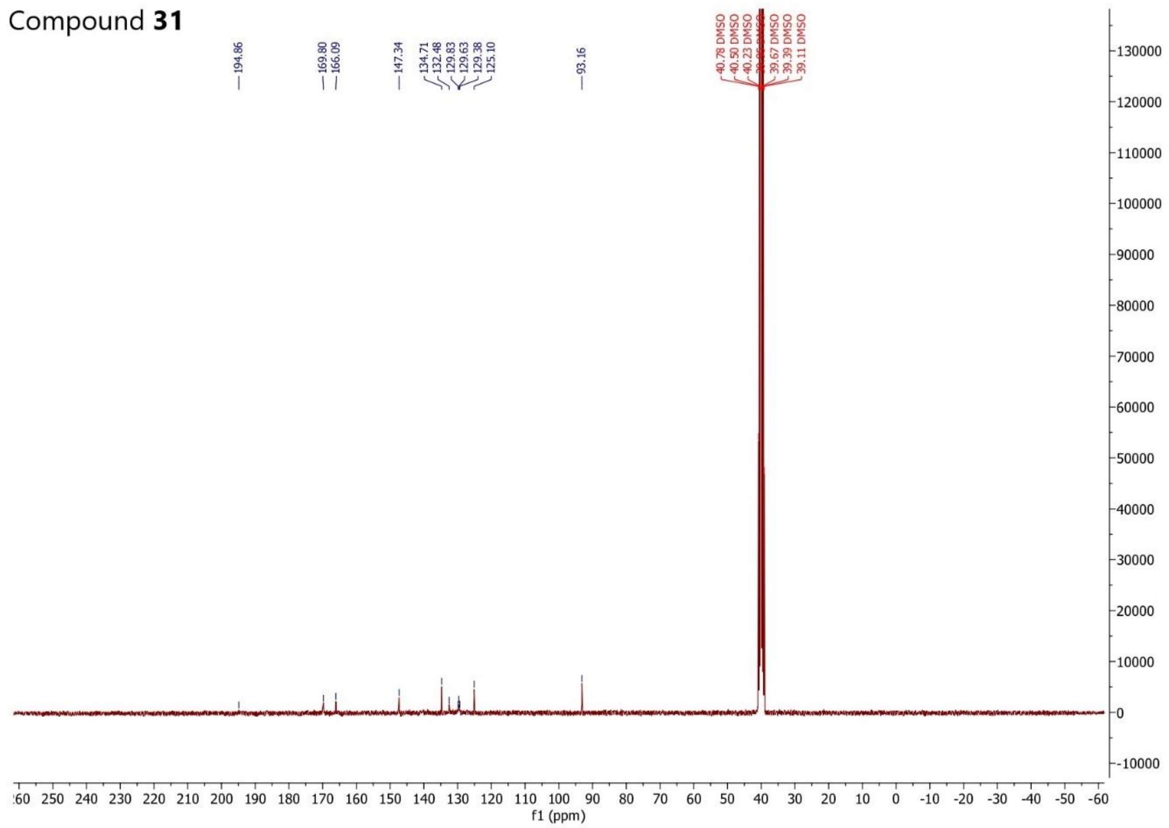

Figure S43. ^{13}C NMR spectrum of compound **31**

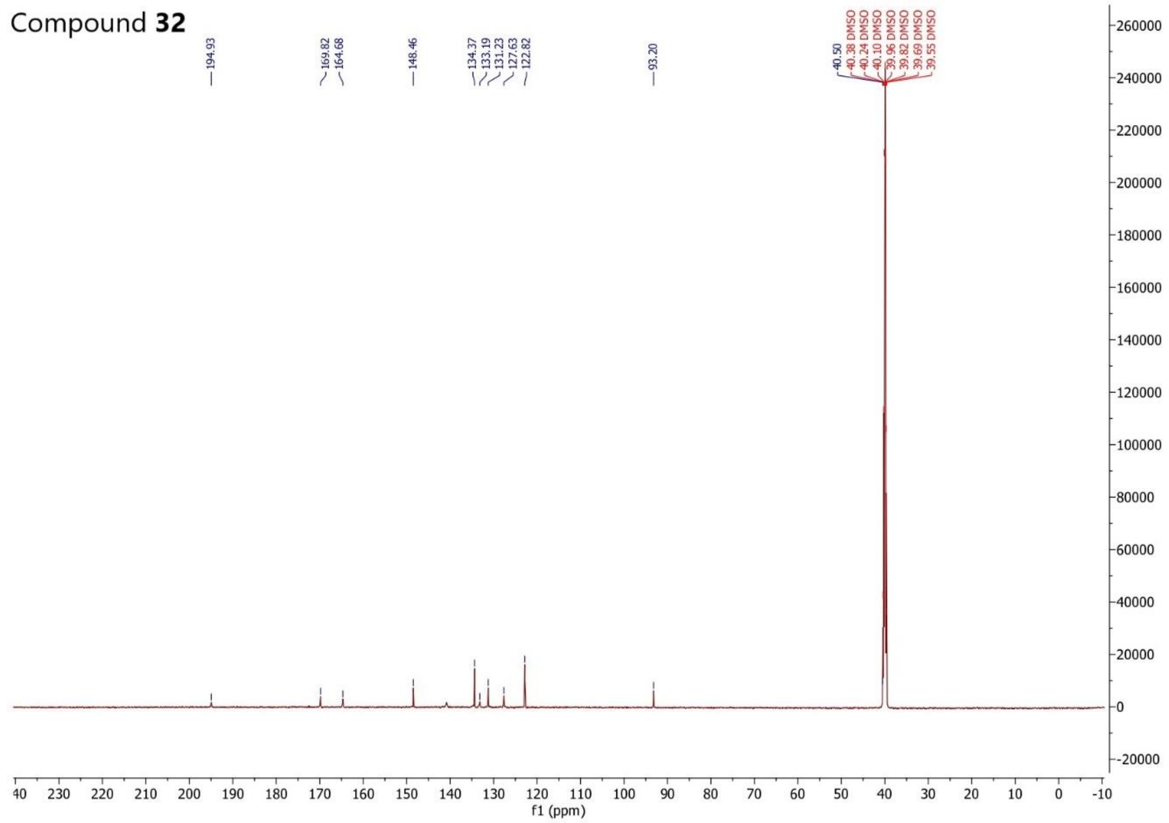

Figure S44. ^{13}C NMR spectrum of compound **32**

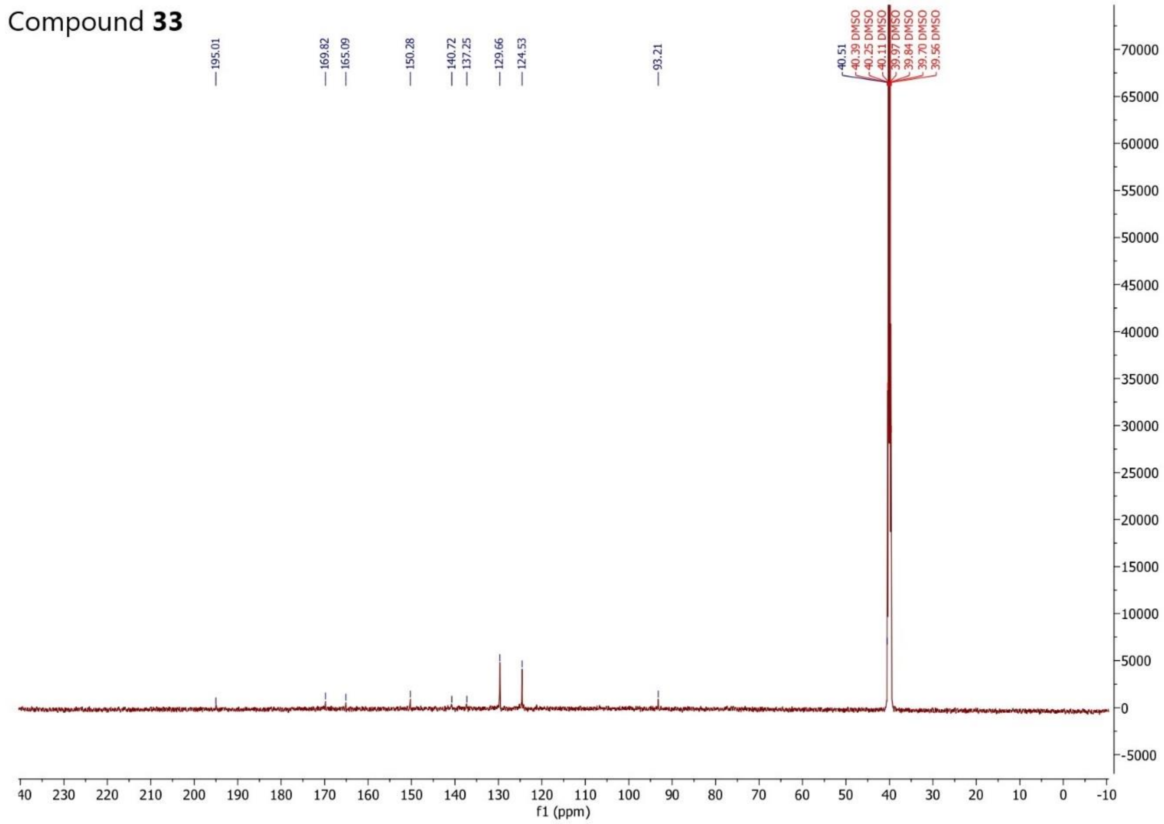

Figure S45. ^{13}C NMR spectrum of compound **33**

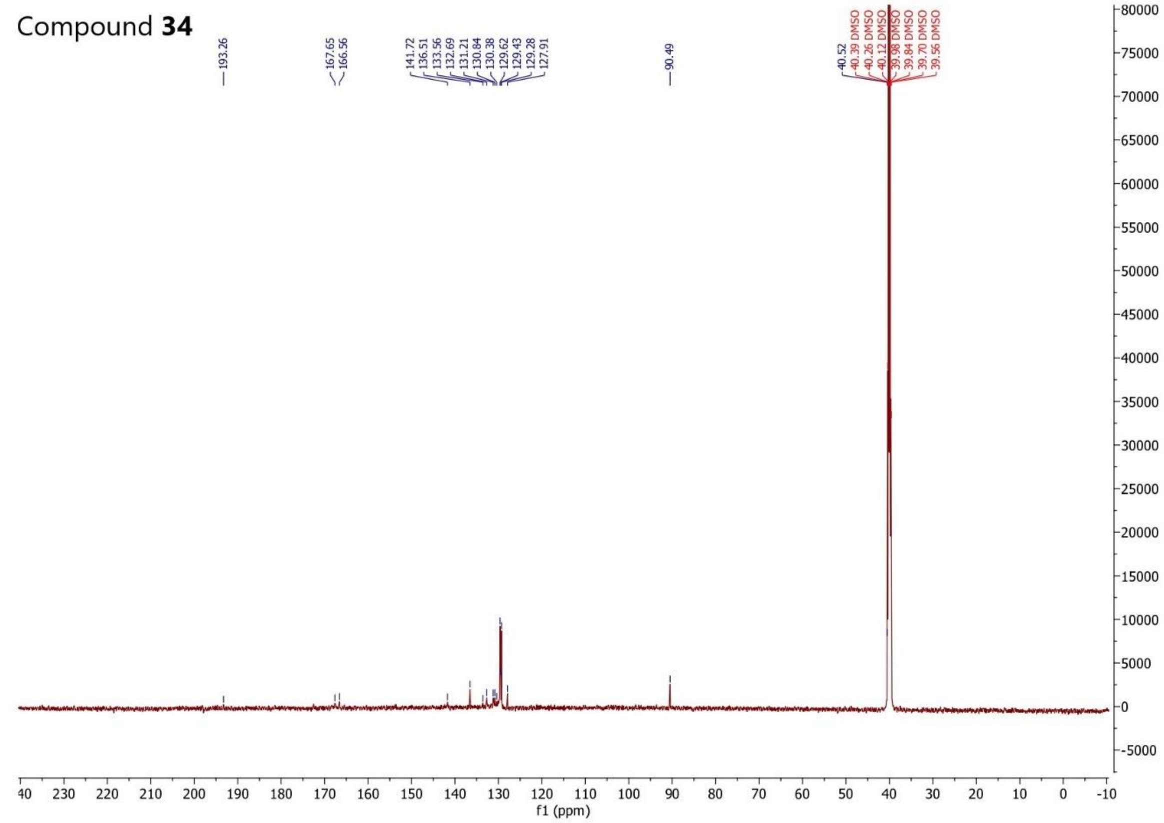

Figure S46. ^{13}C NMR spectrum of compound **34**

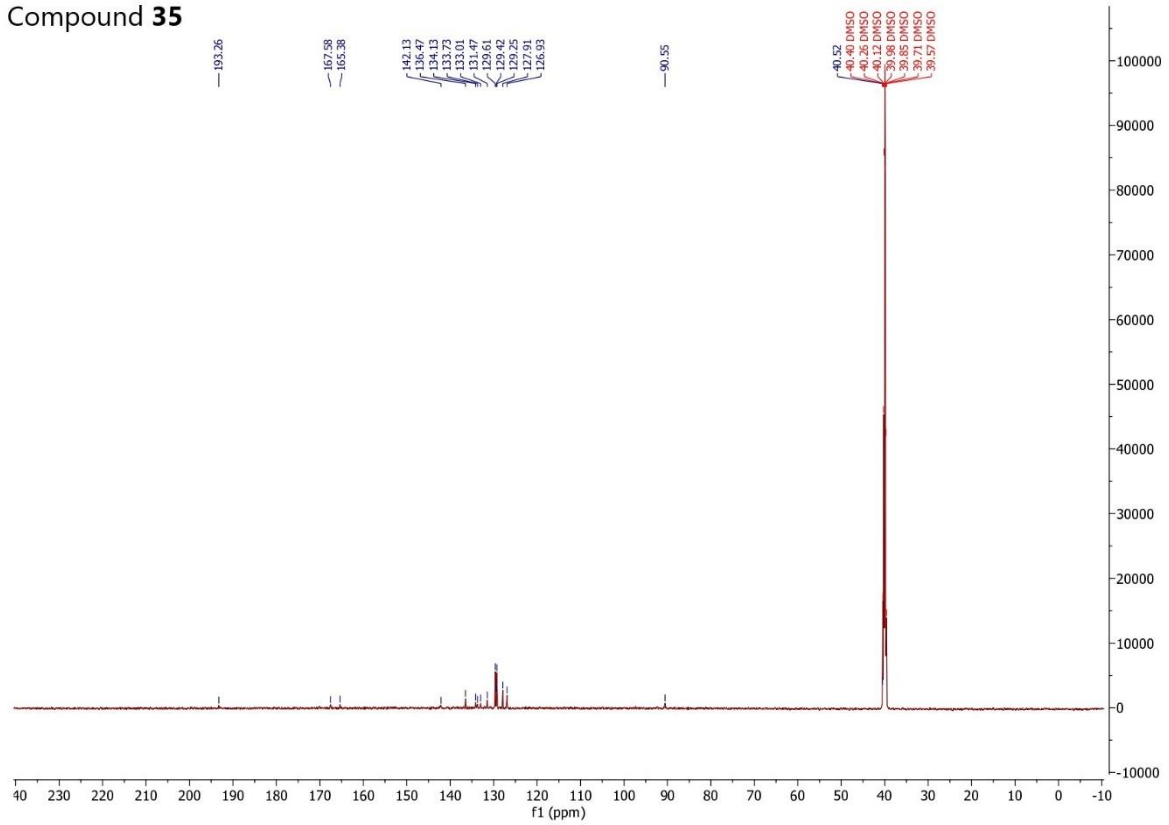

Figure S47. ^{13}C NMR spectrum of compound **35**

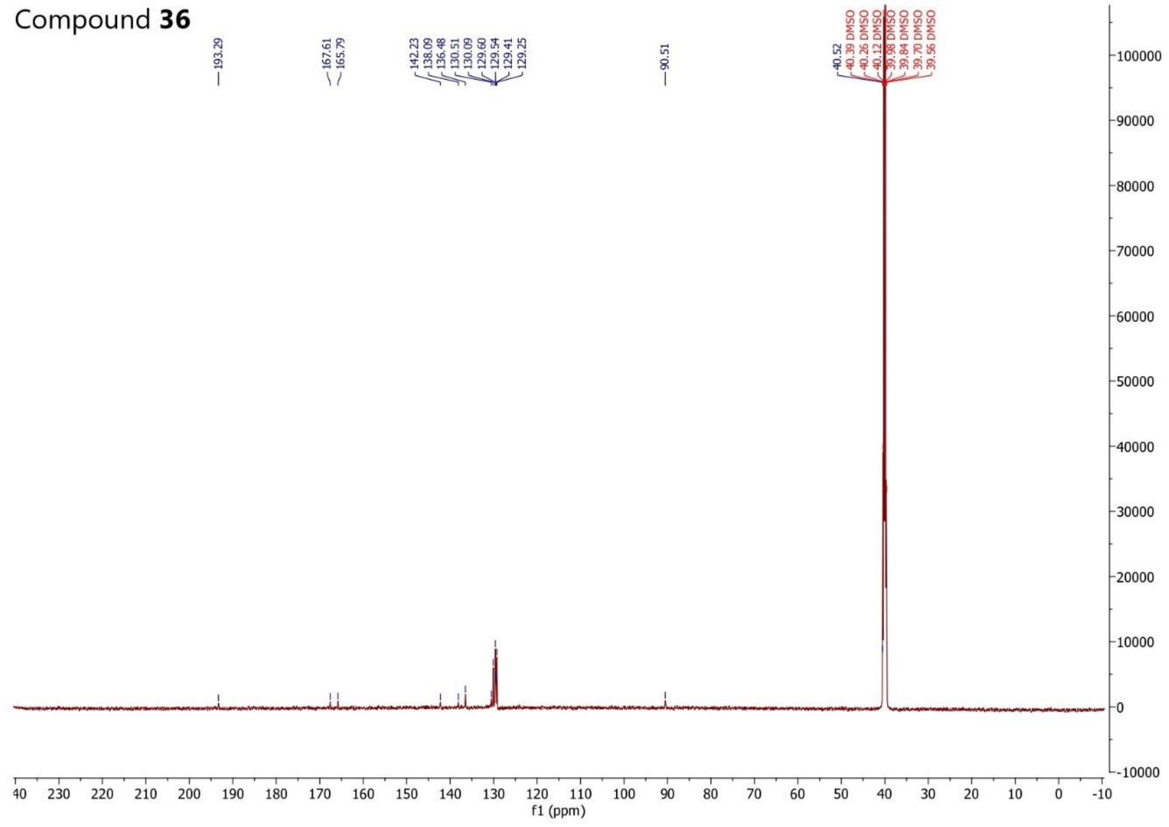

Figure S48. ^{13}C NMR spectrum of compound **36**

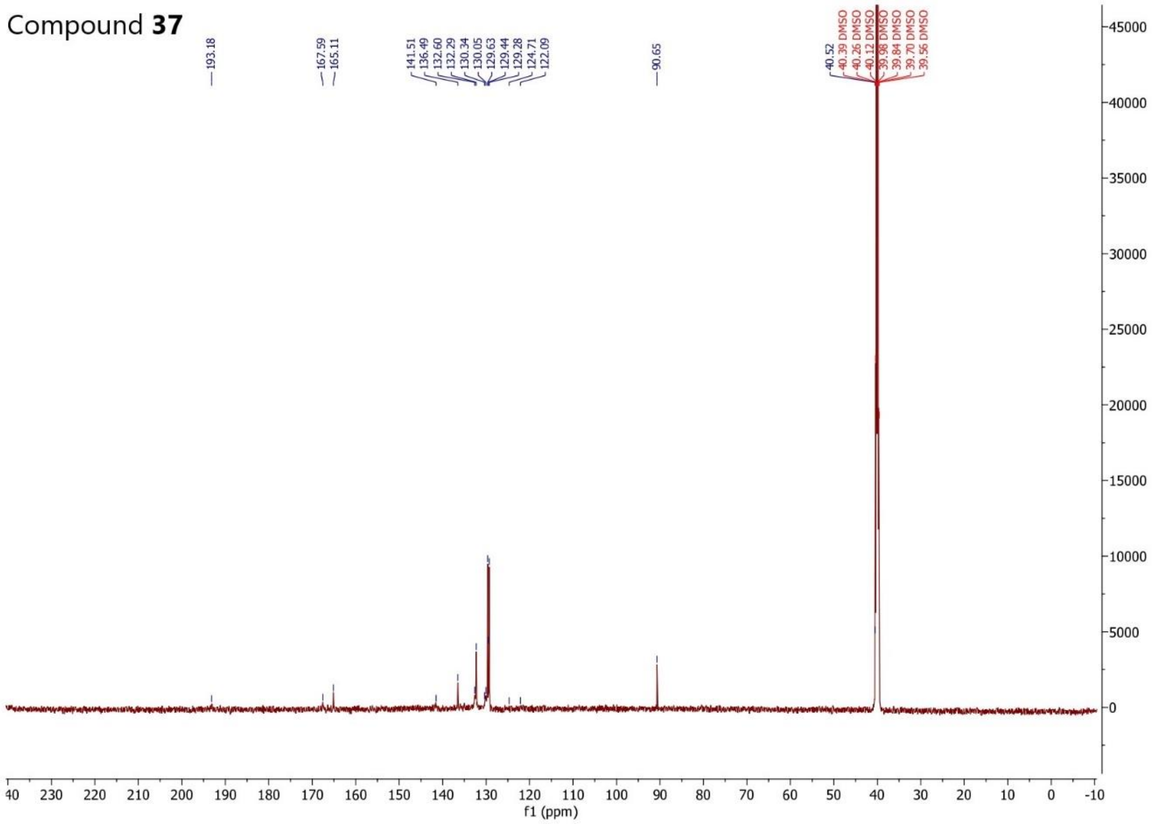

Figure S49. ^{13}C NMR spectrum of compound **37**

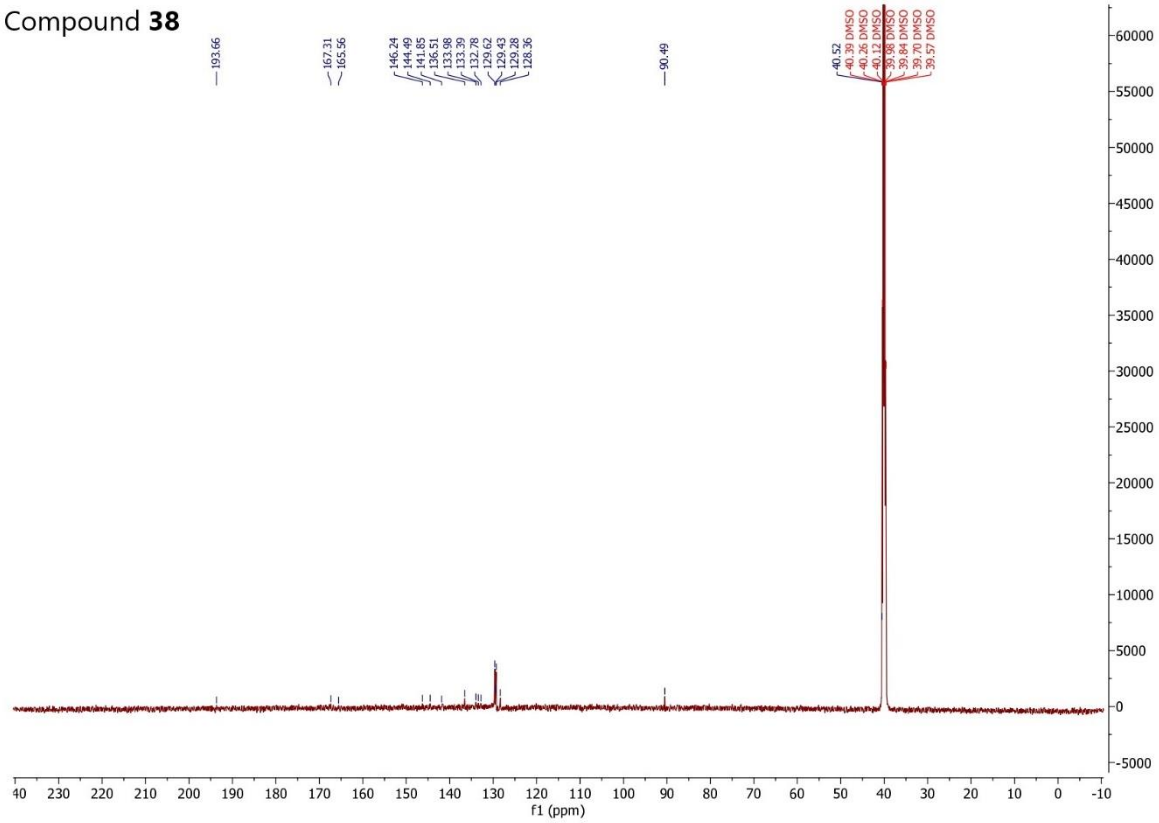

Figure S50. ^{13}C NMR spectrum of compound **38**

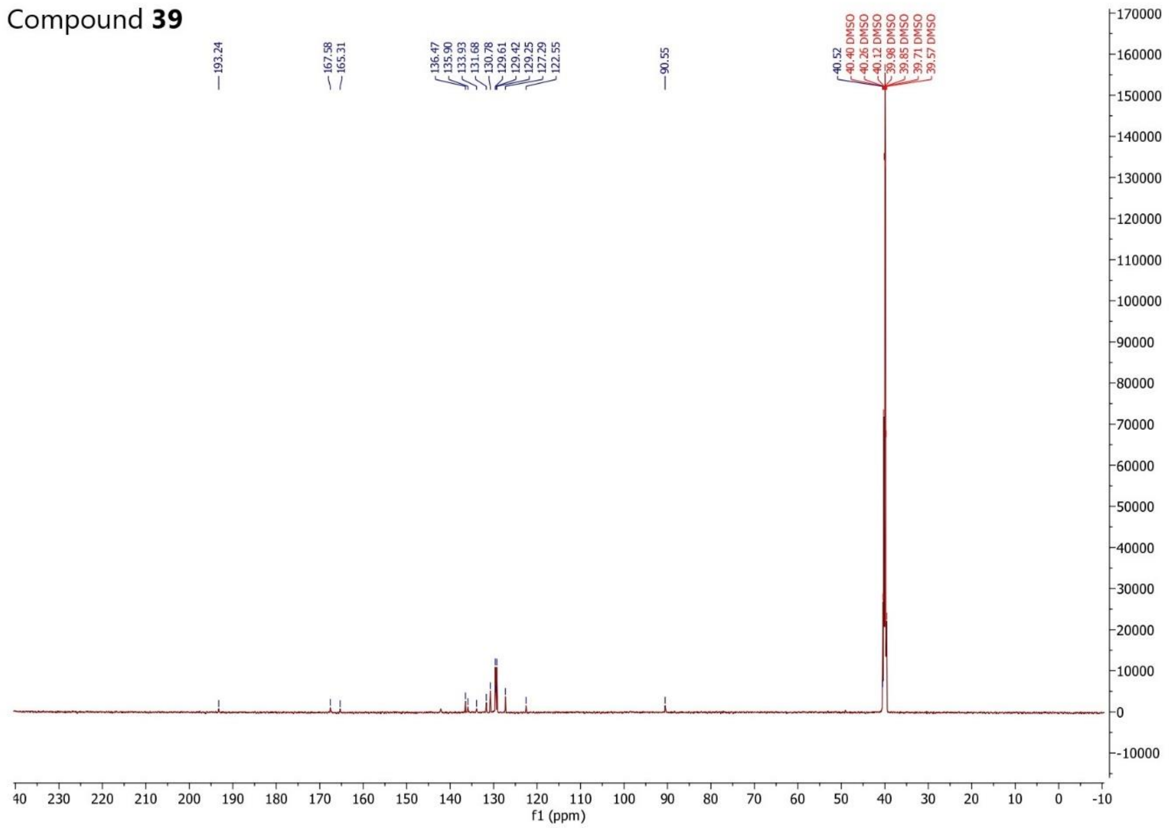

Figure S51. ^{13}C NMR spectrum of compound **39**

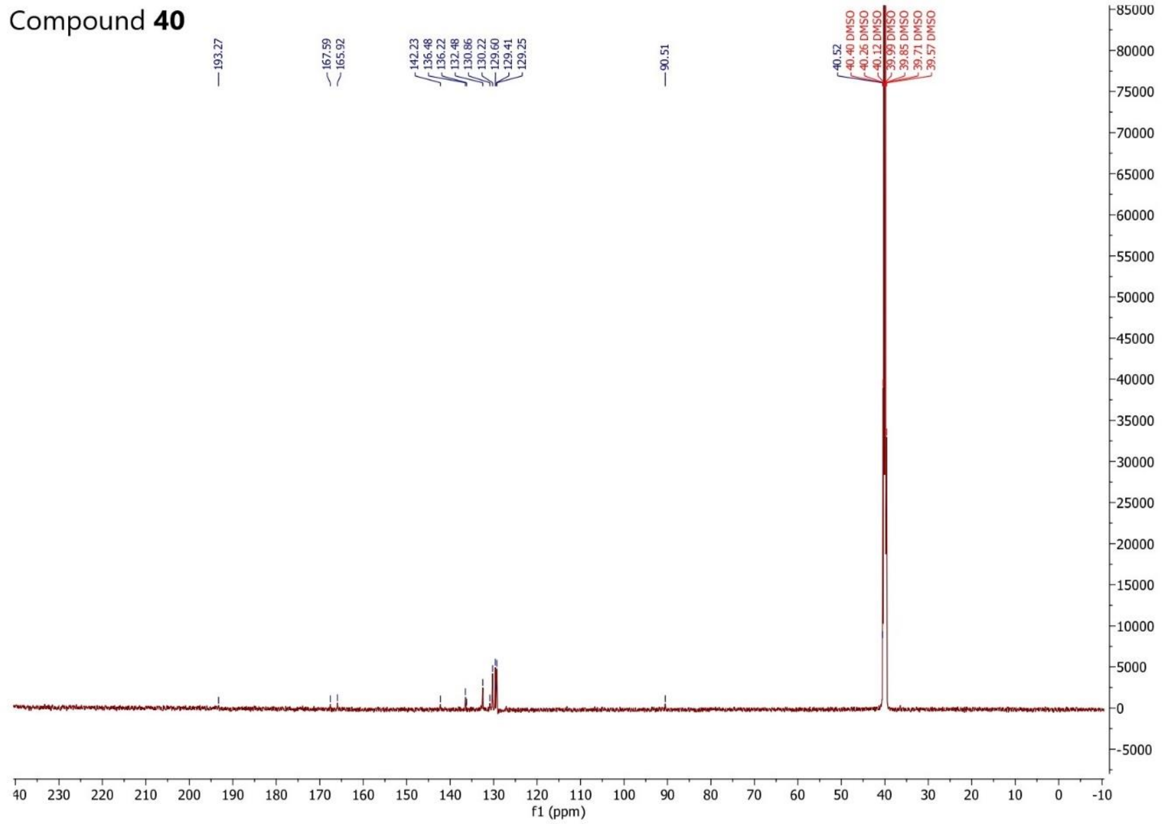

Figure S52. ^{13}C NMR spectrum of compound **40**

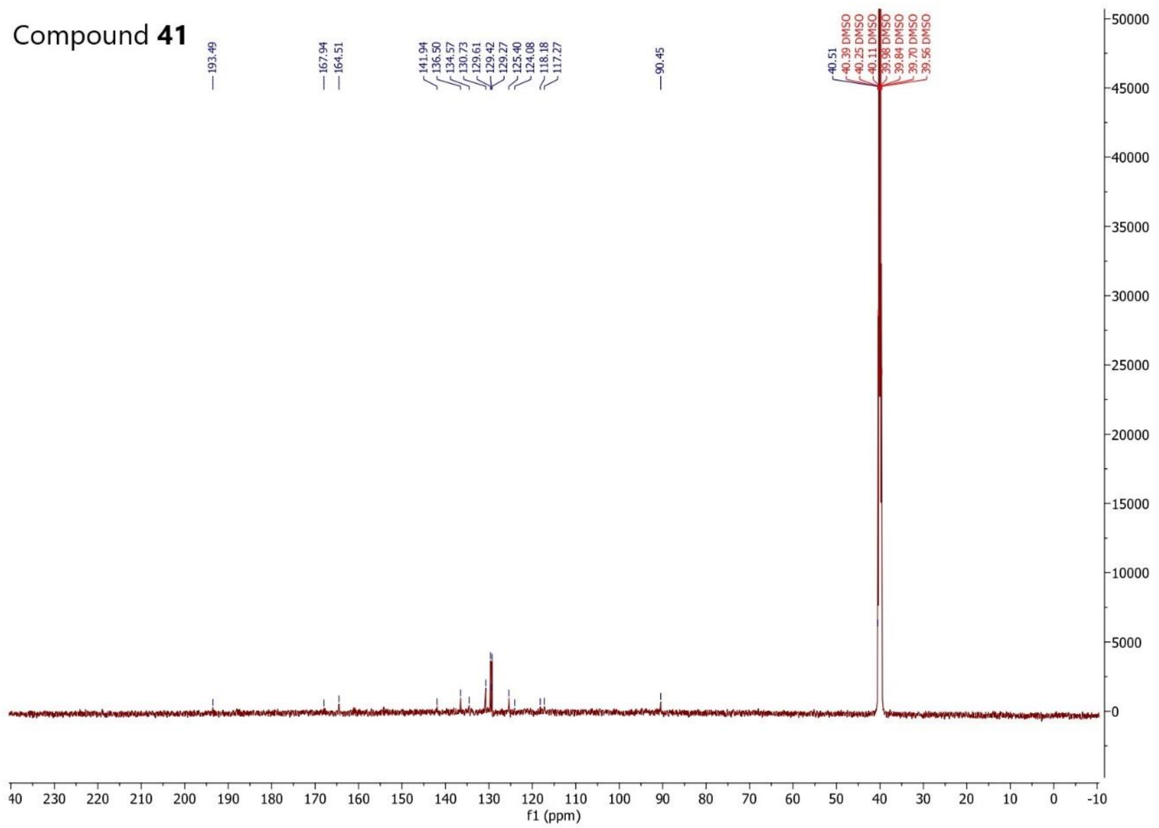

Figure S53. ^{13}C NMR spectrum of compound **41**

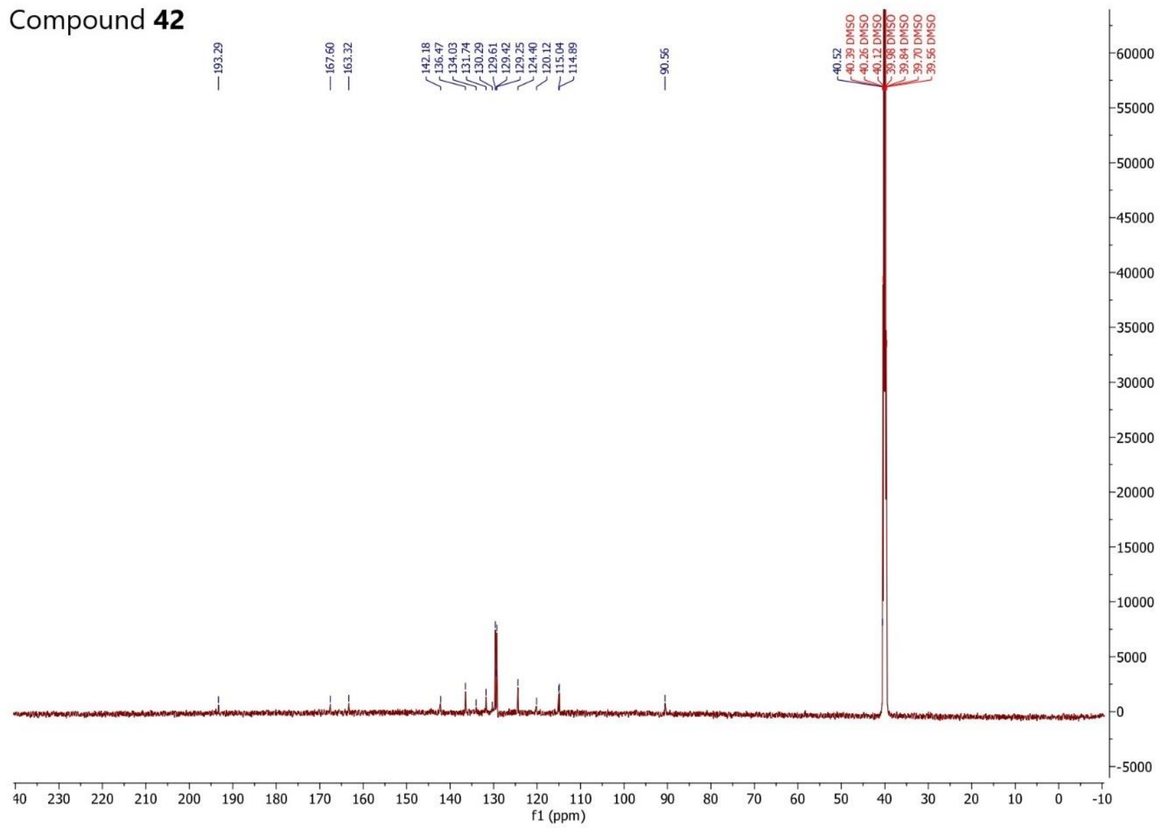

Figure S54. ^{13}C NMR spectrum of compound **42**

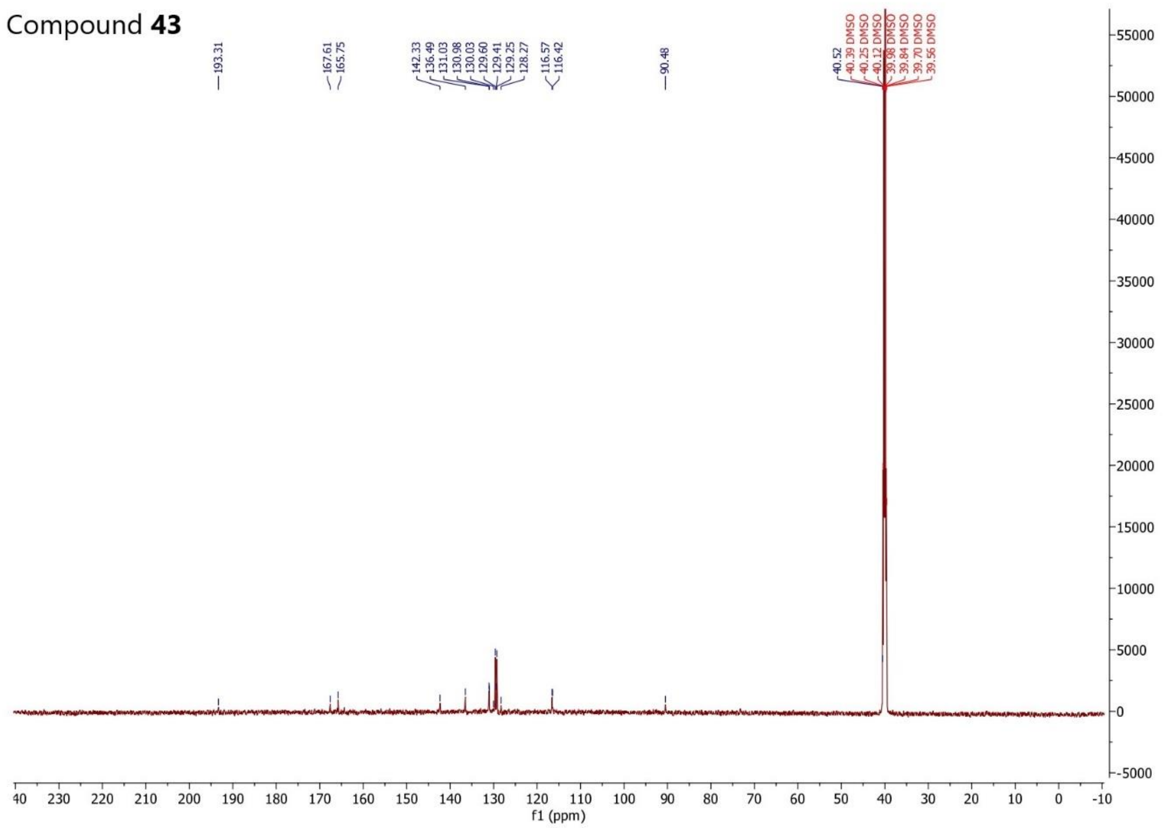

Figure S55. ^{13}C NMR spectrum of compound **43**

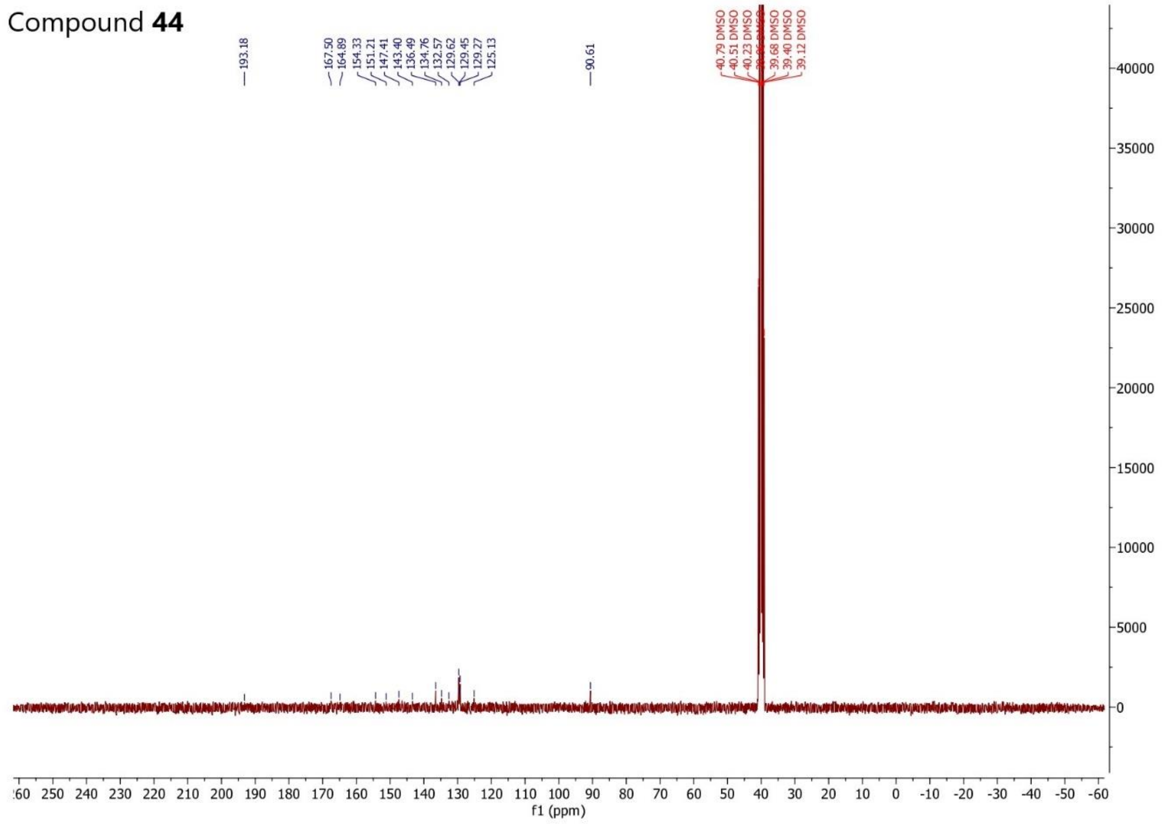

Figure S56. ^{13}C NMR spectrum of compound **44**

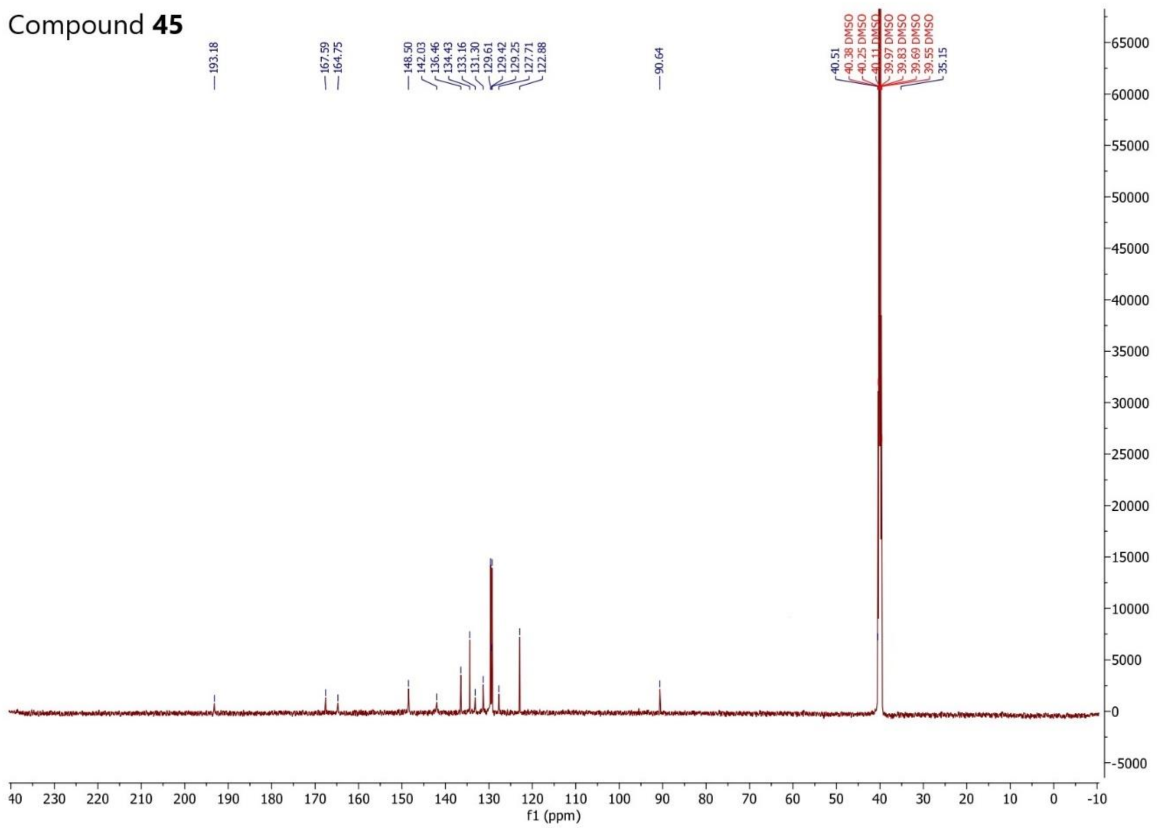

Figure S57. ^{13}C NMR spectrum of compound **45**

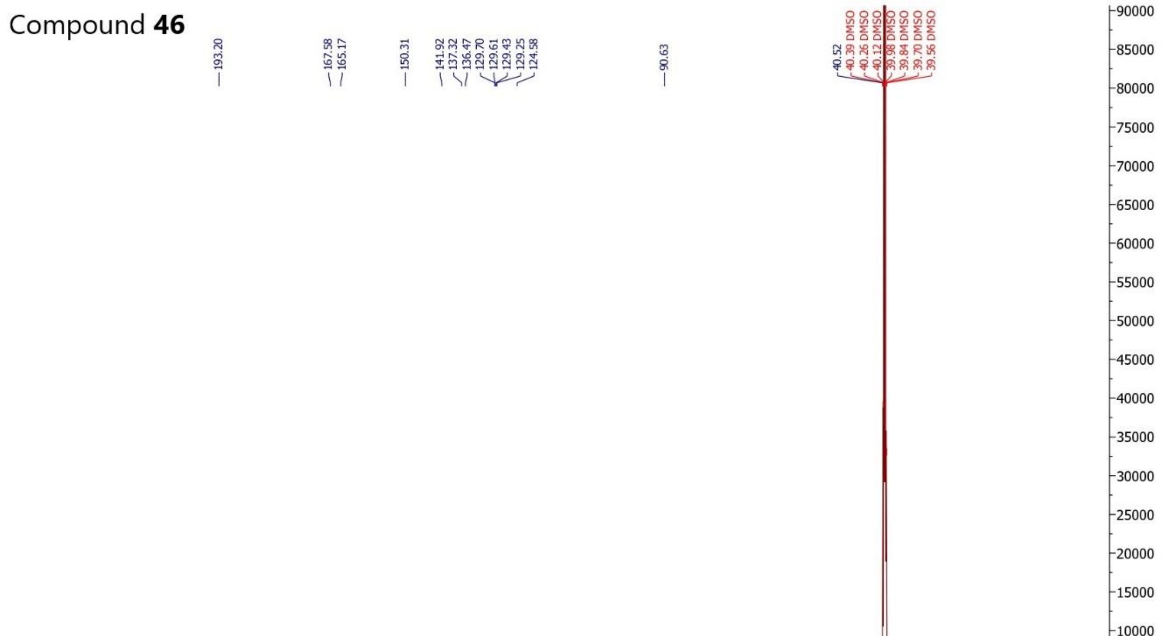

Figure S58. ^{13}C NMR spectrum of compound **46**

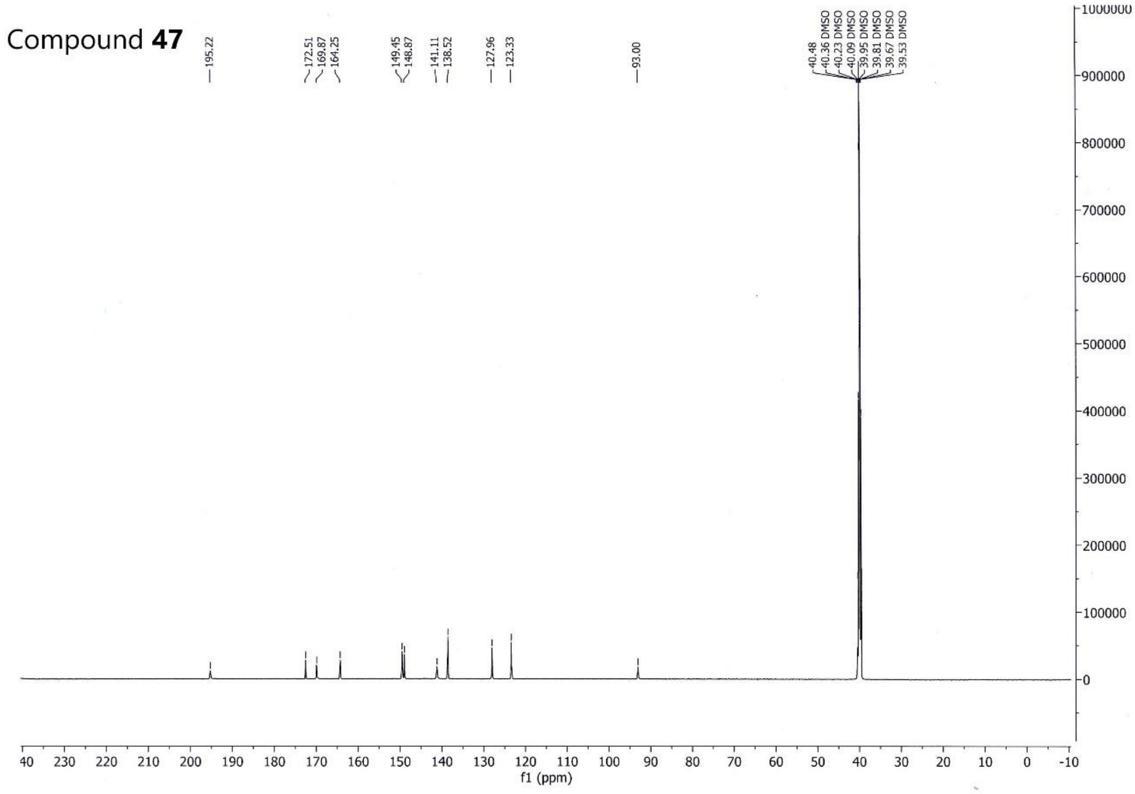

Figure S59. ^{13}C NMR spectrum of compound **47**

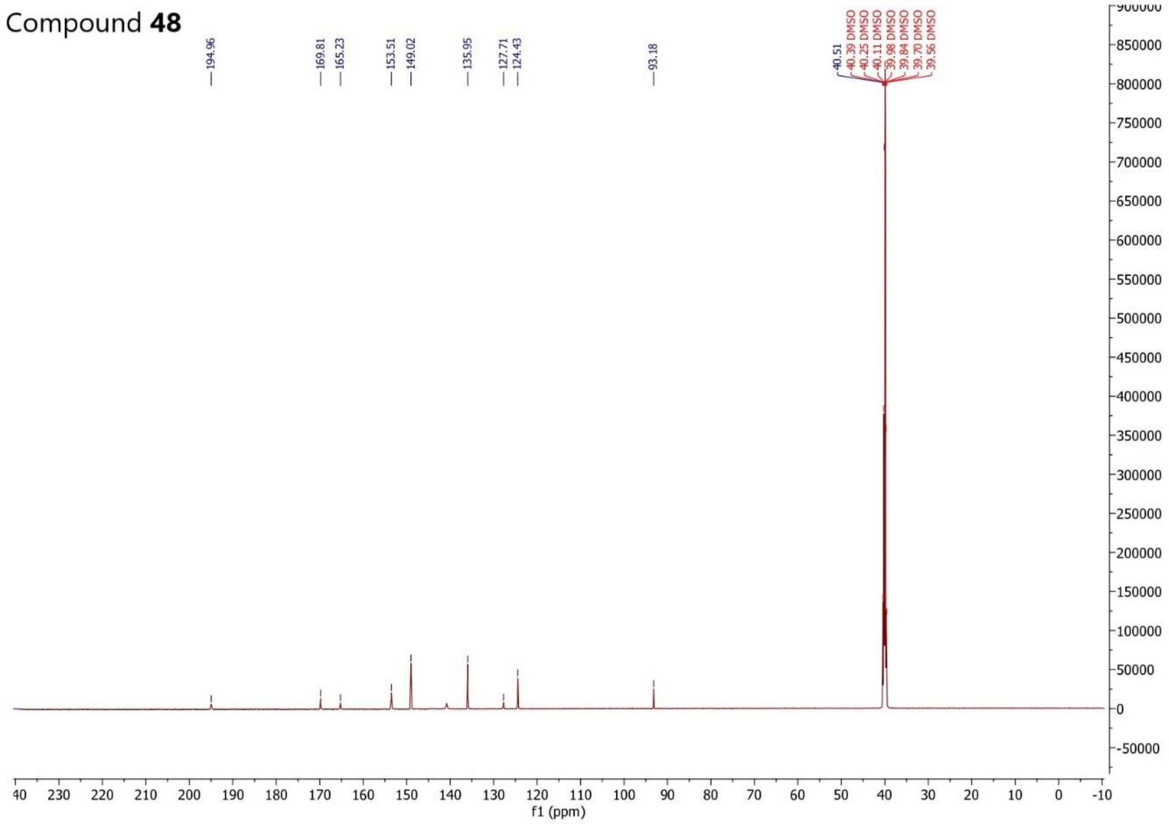

Figure S60. ^{13}C NMR spectrum of compound **48**

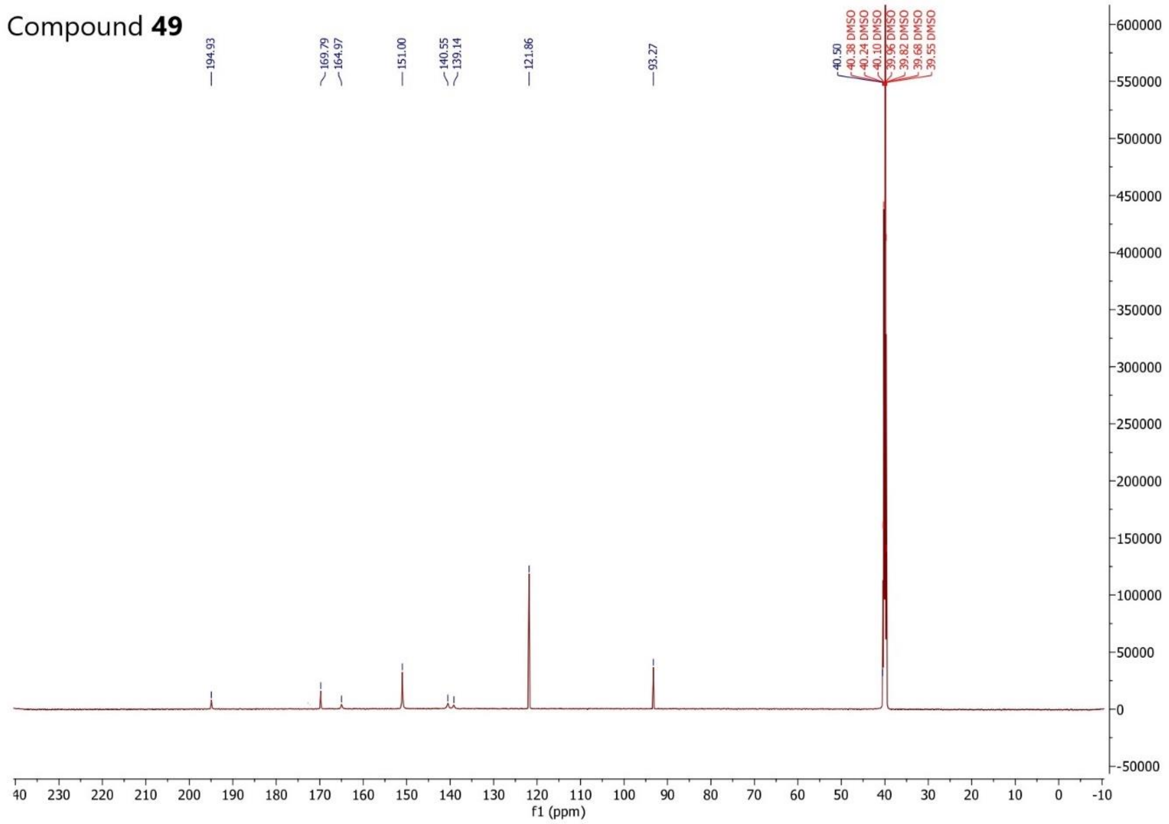

Figure S61. ^{13}C NMR spectrum of compound **49**

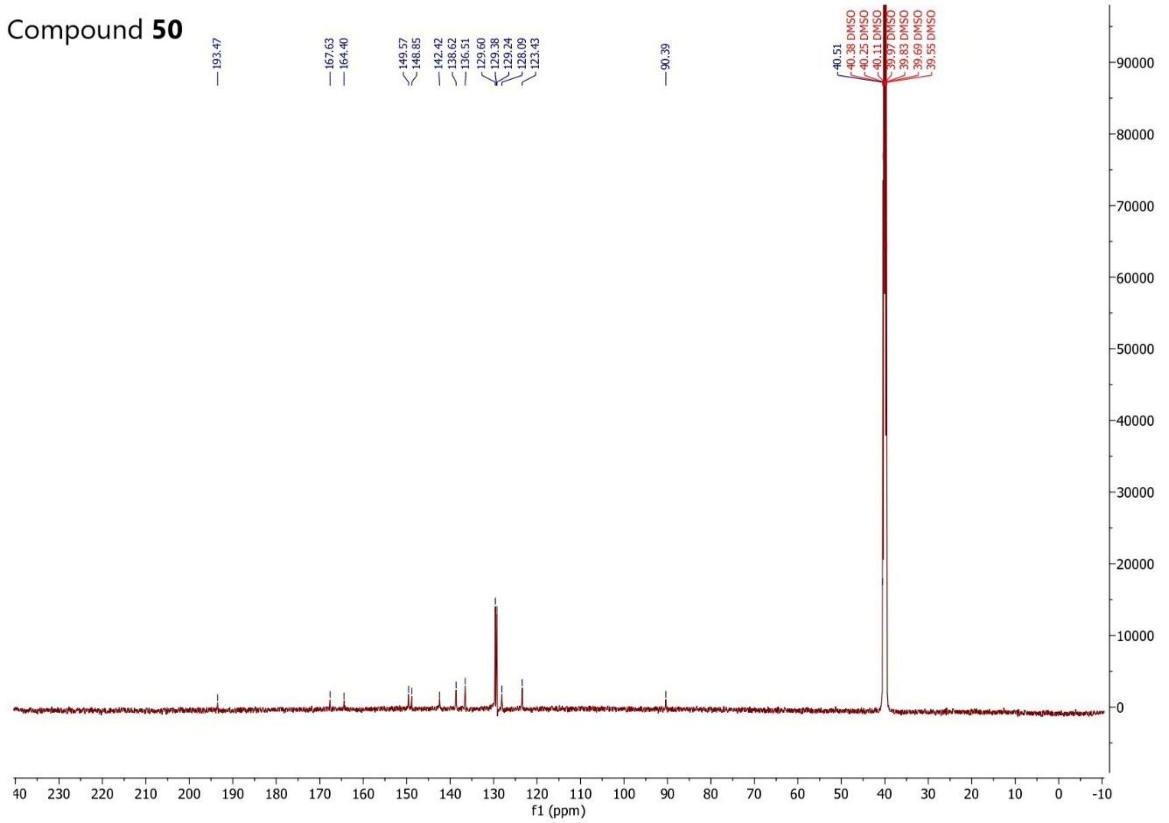

Figure S62. ^{13}C NMR spectrum of compound **50**

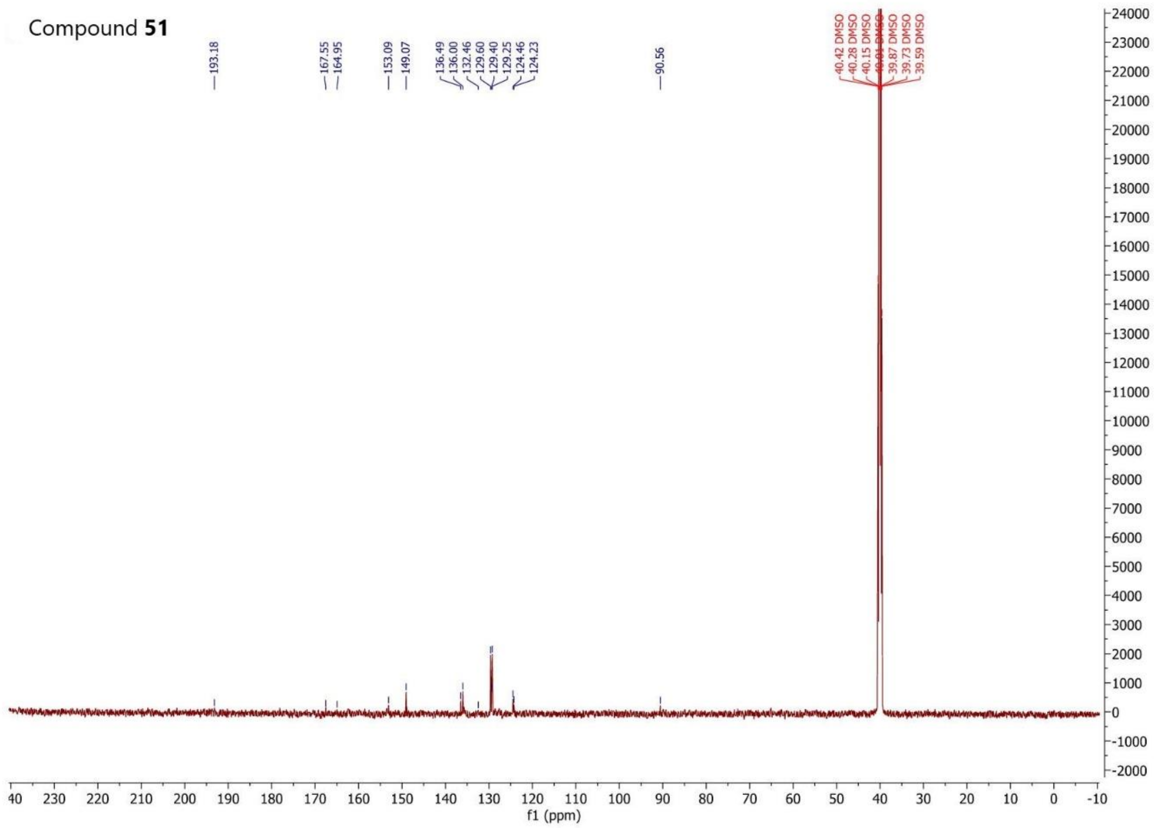

Figure S63. ^{13}C NMR spectrum of compound **51**

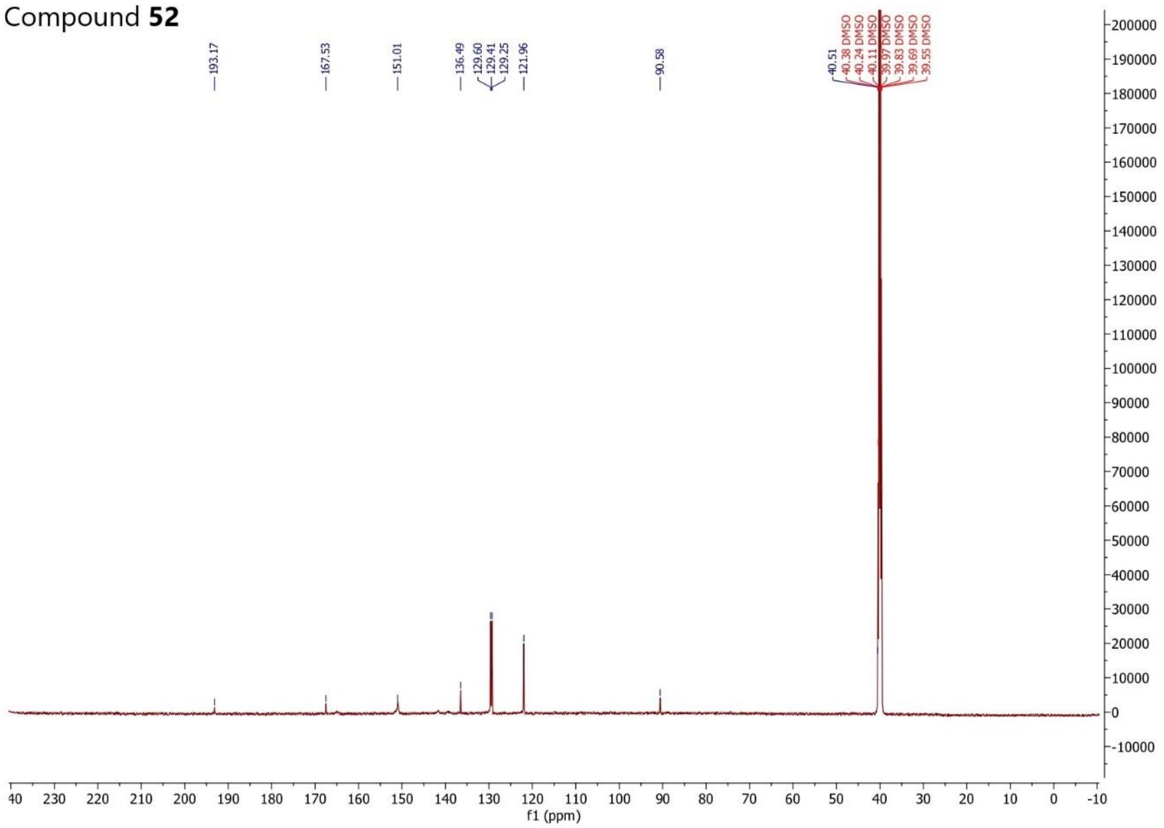

Figure S64. ^{13}C NMR spectrum of compound **52**

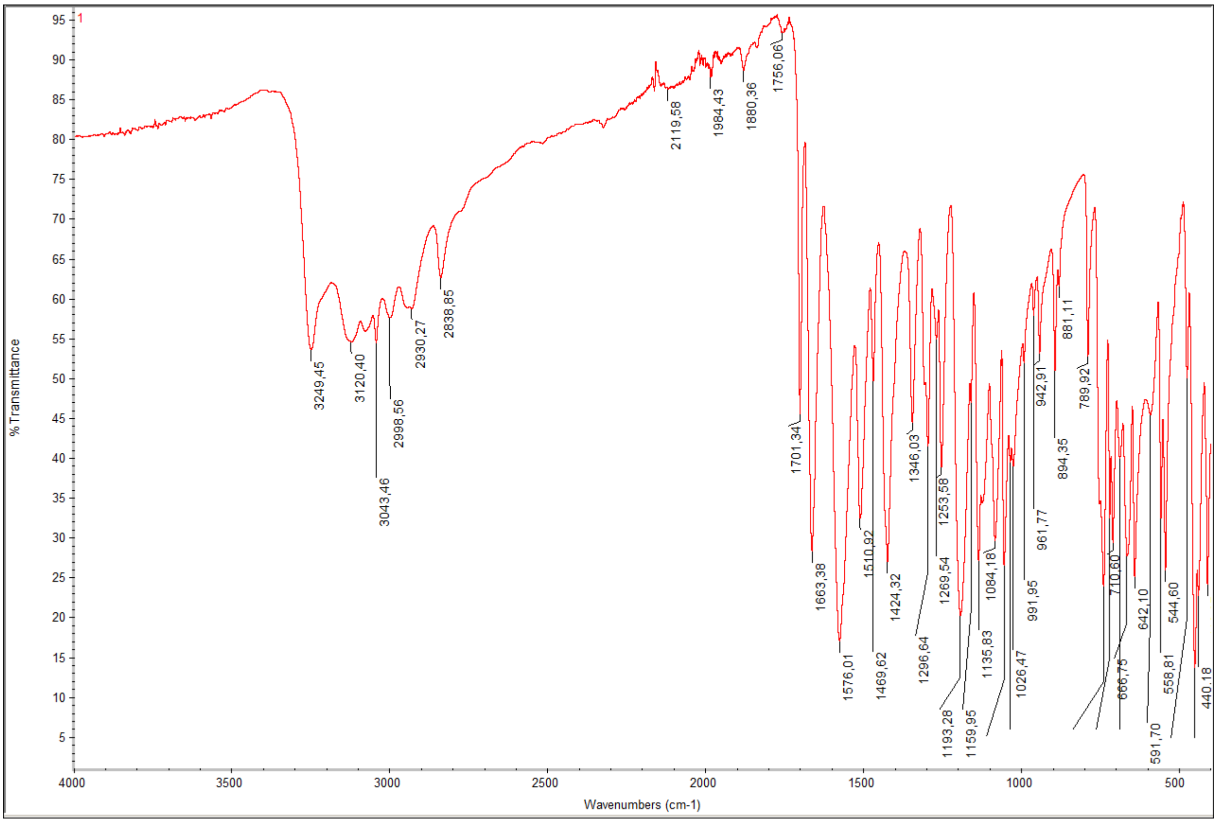

Figure S65. FT-IR spectrum of compound **21**

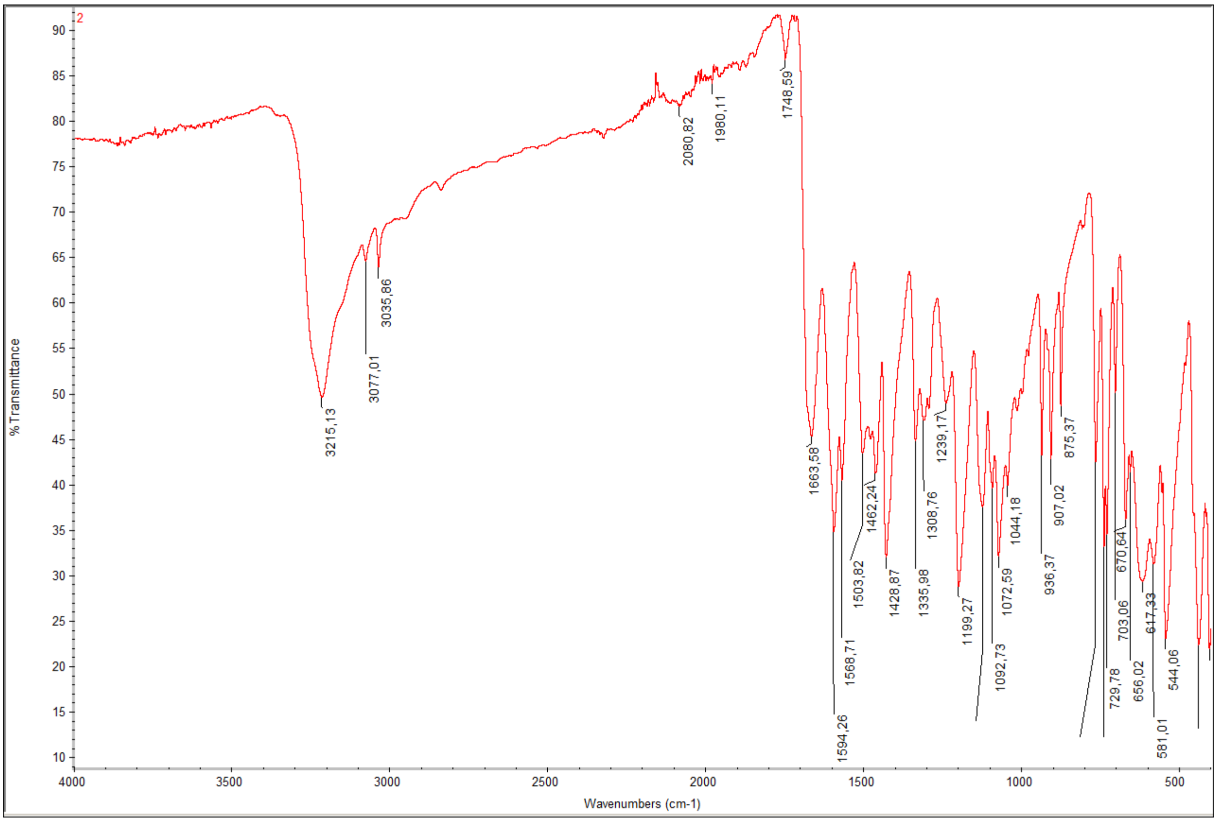

Figure S66. FT-IR spectrum of compound **22**

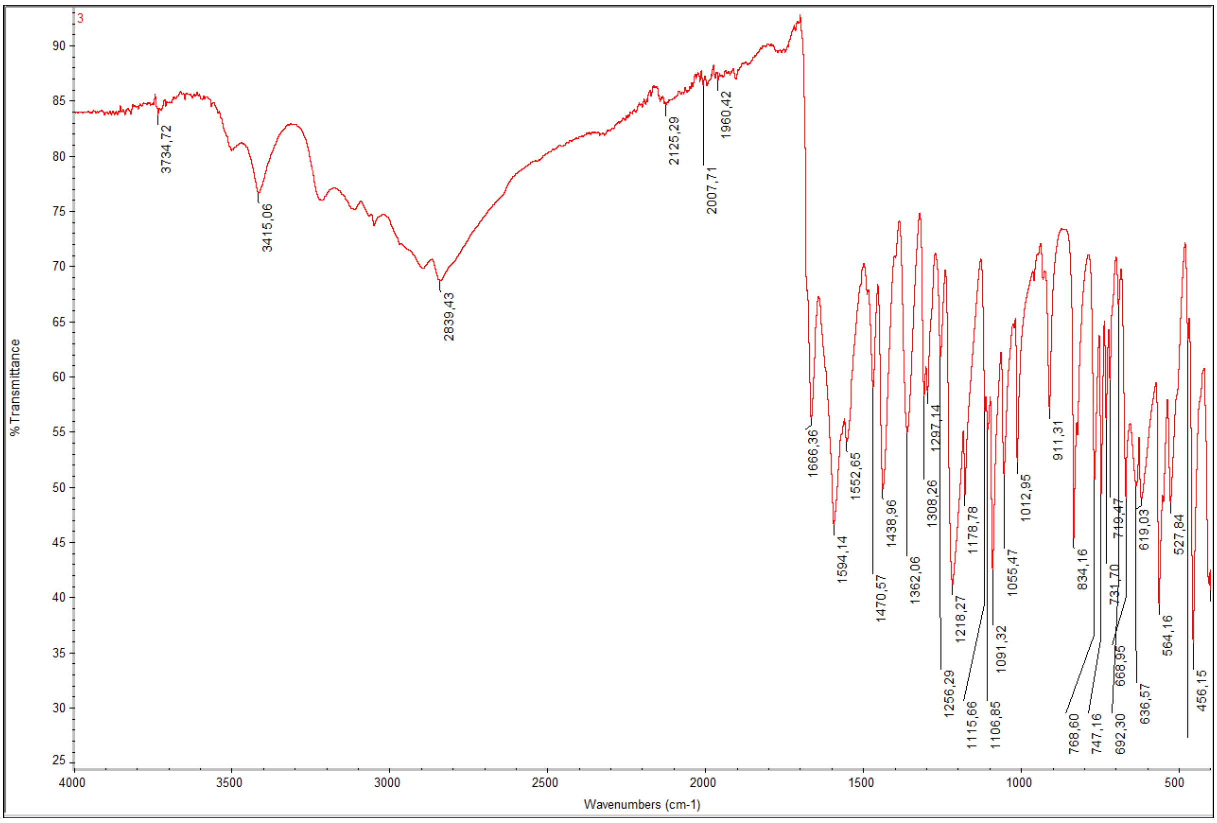

Figure S67. FT-IR spectrum of compound **23**

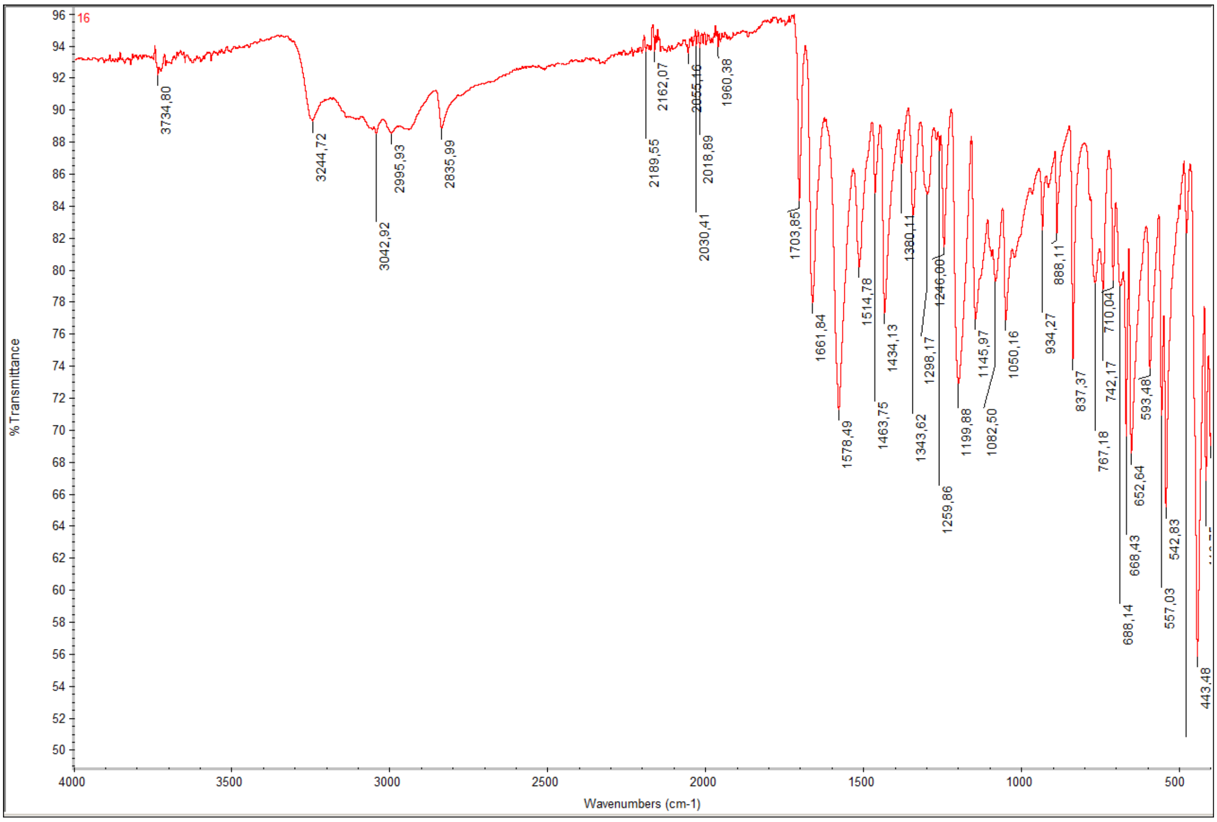

Figure S68. FT-IR spectrum of compound **24**

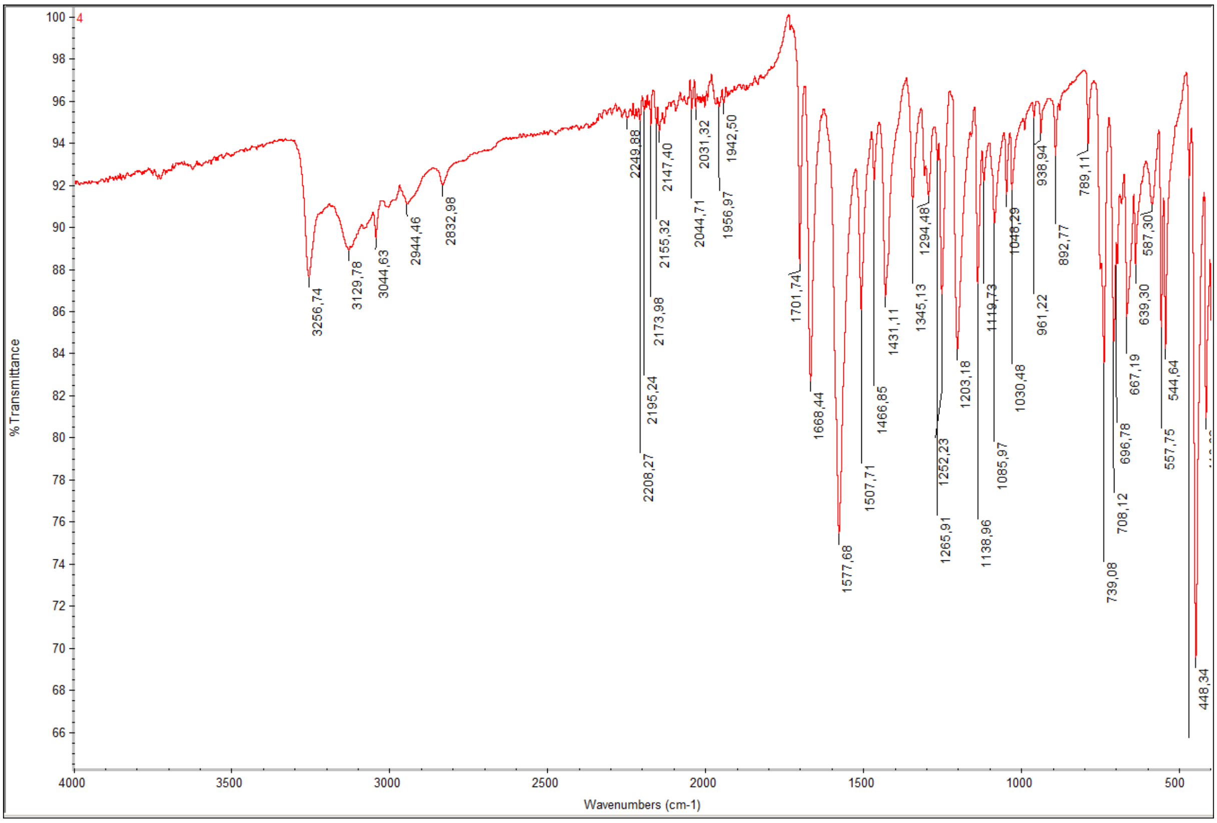

Figure S69. FT-IR spectrum of compound **25**

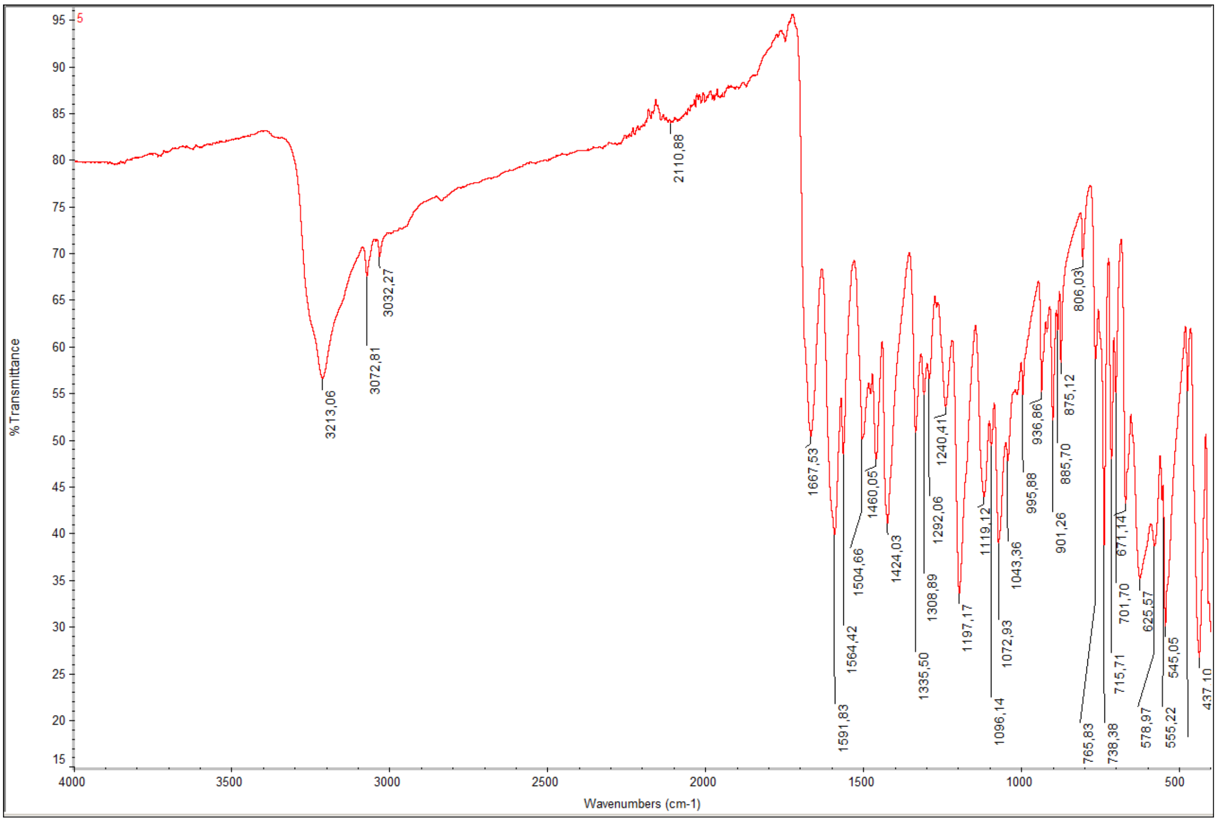

Figure S70. FT-IR spectrum of compound **26**

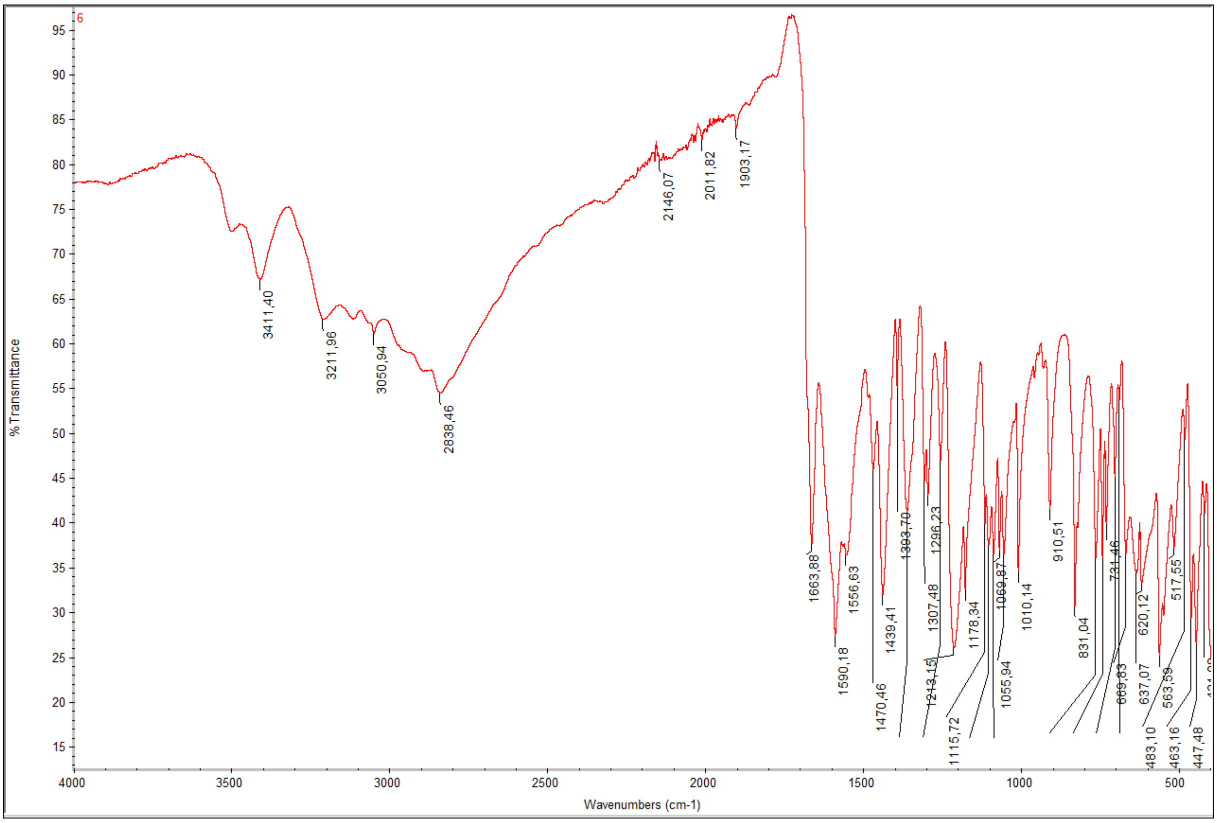

Figure S71. FT-IR spectrum of compound **27**

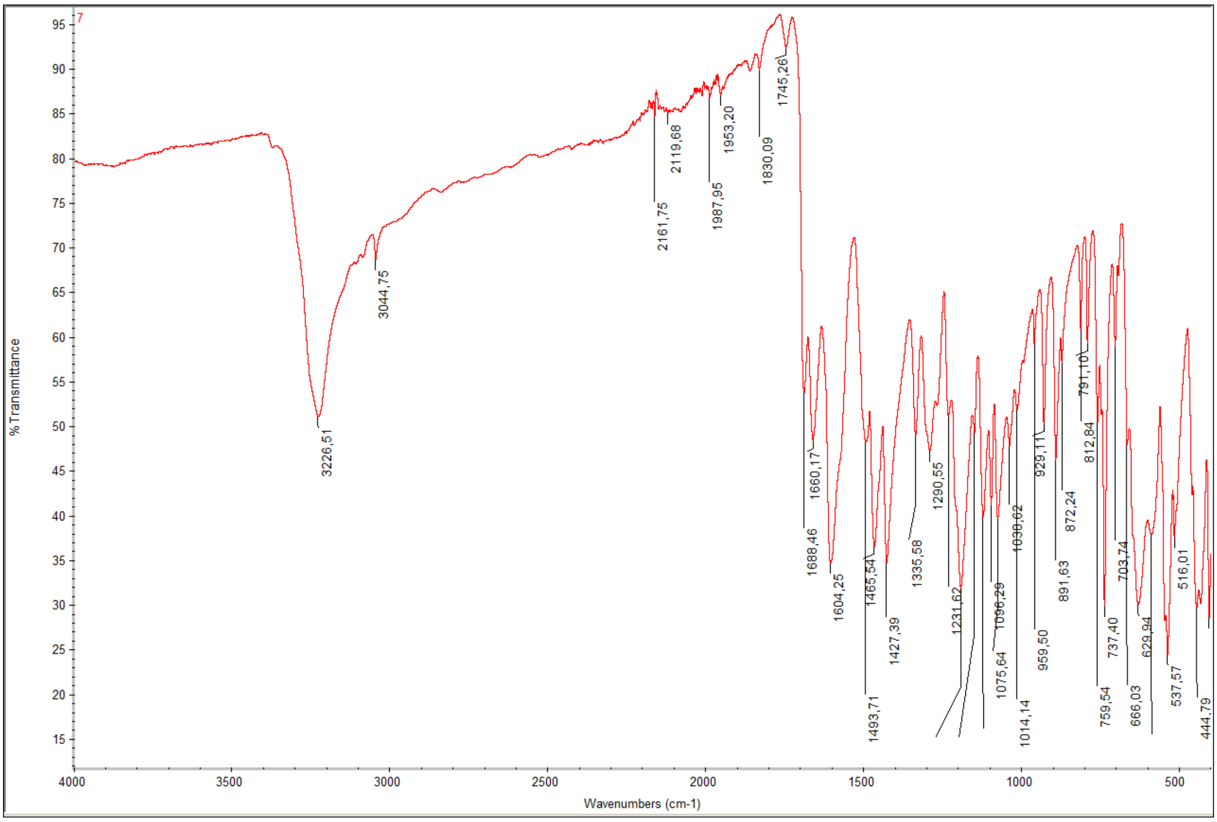

Figure S72. FT-IR spectrum of compound **28**

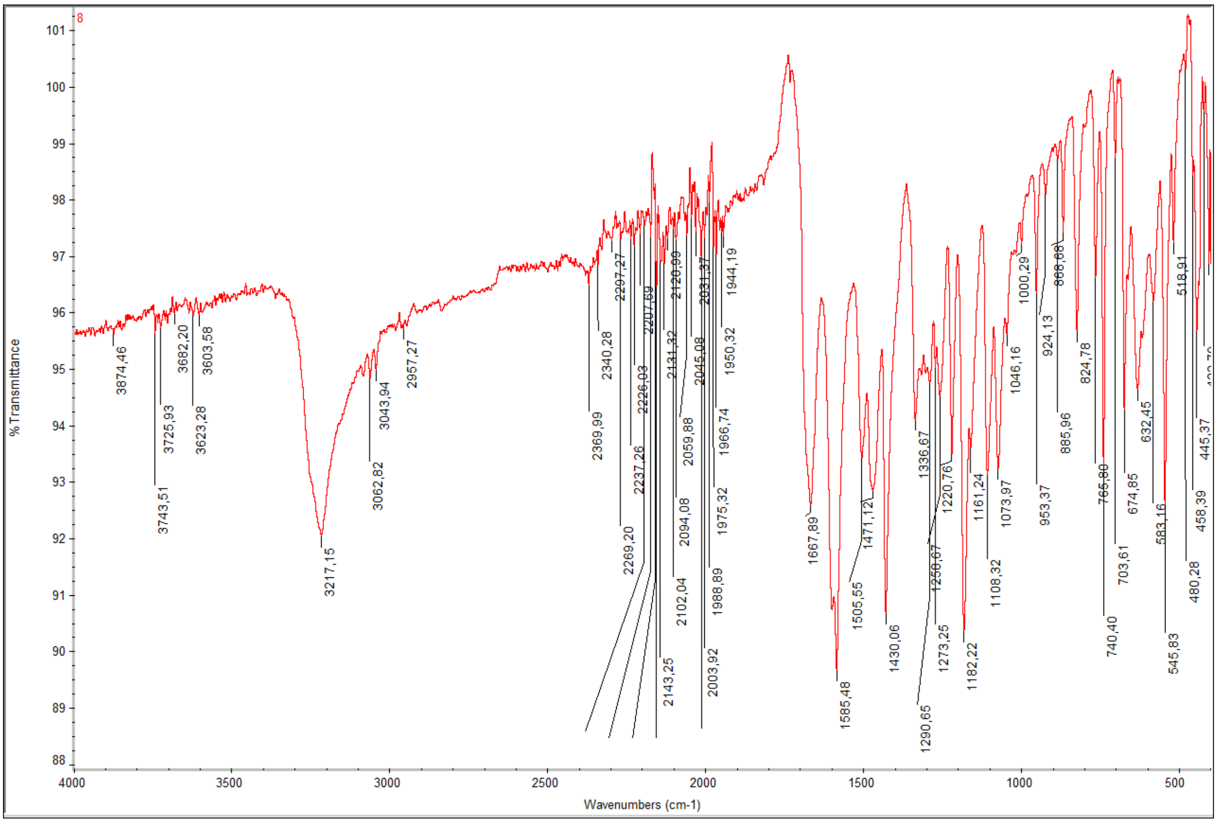

Figure S73. FT-IR spectrum of compound **29**

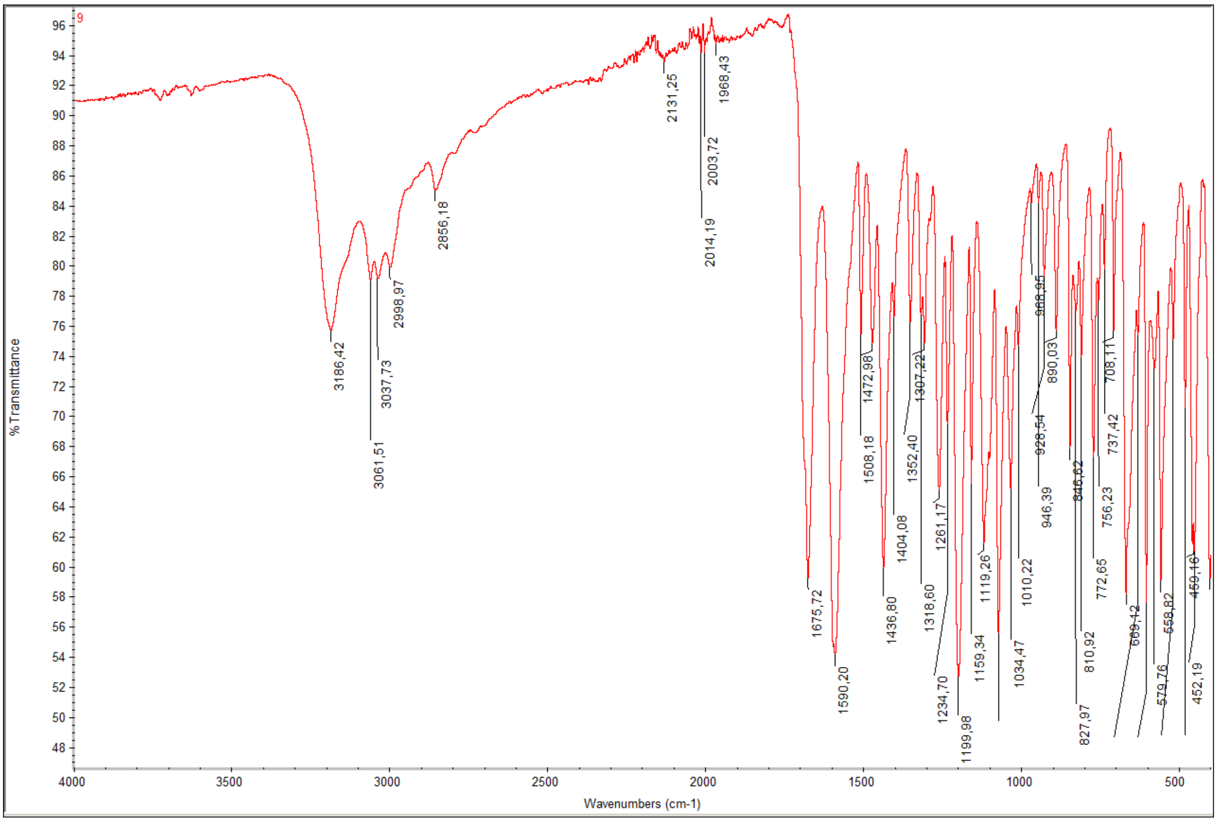

Figure S74. FT-IR spectrum of compound **30**

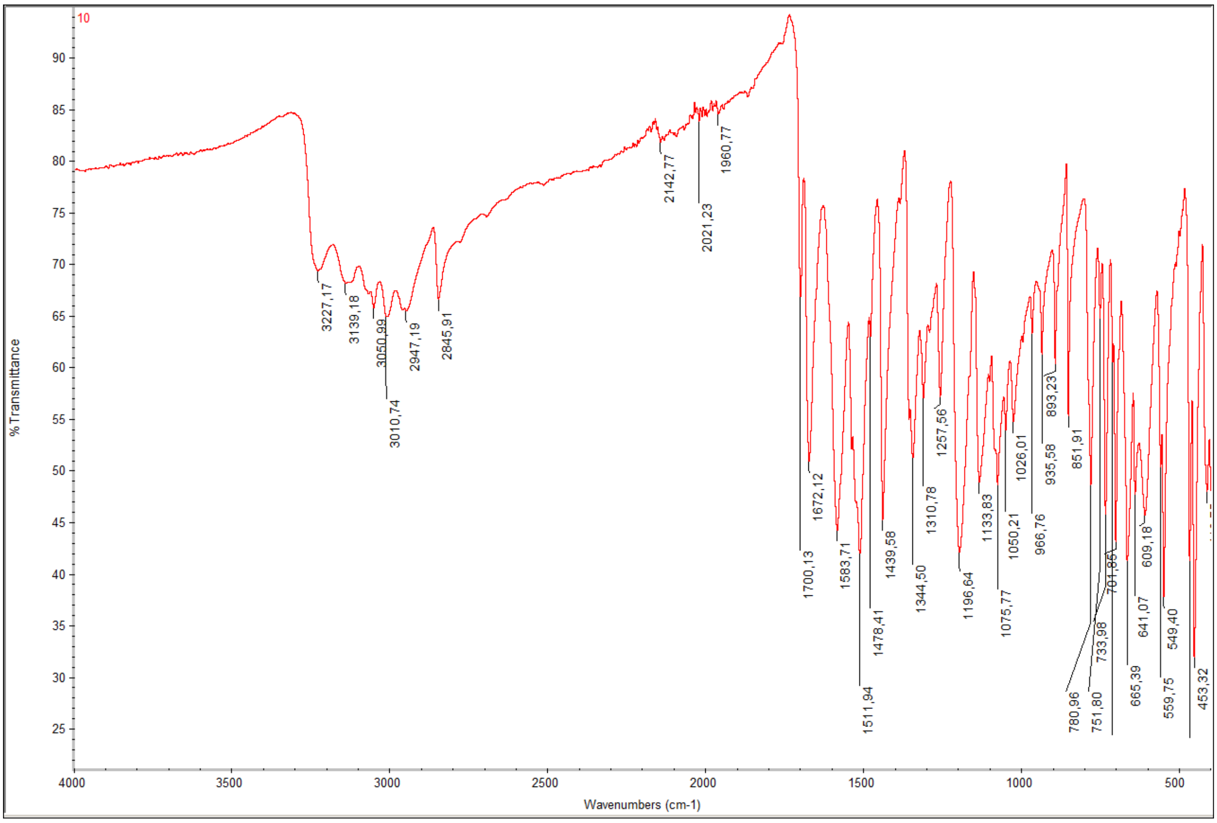

Figure S75. FT-IR spectrum of compound **31**

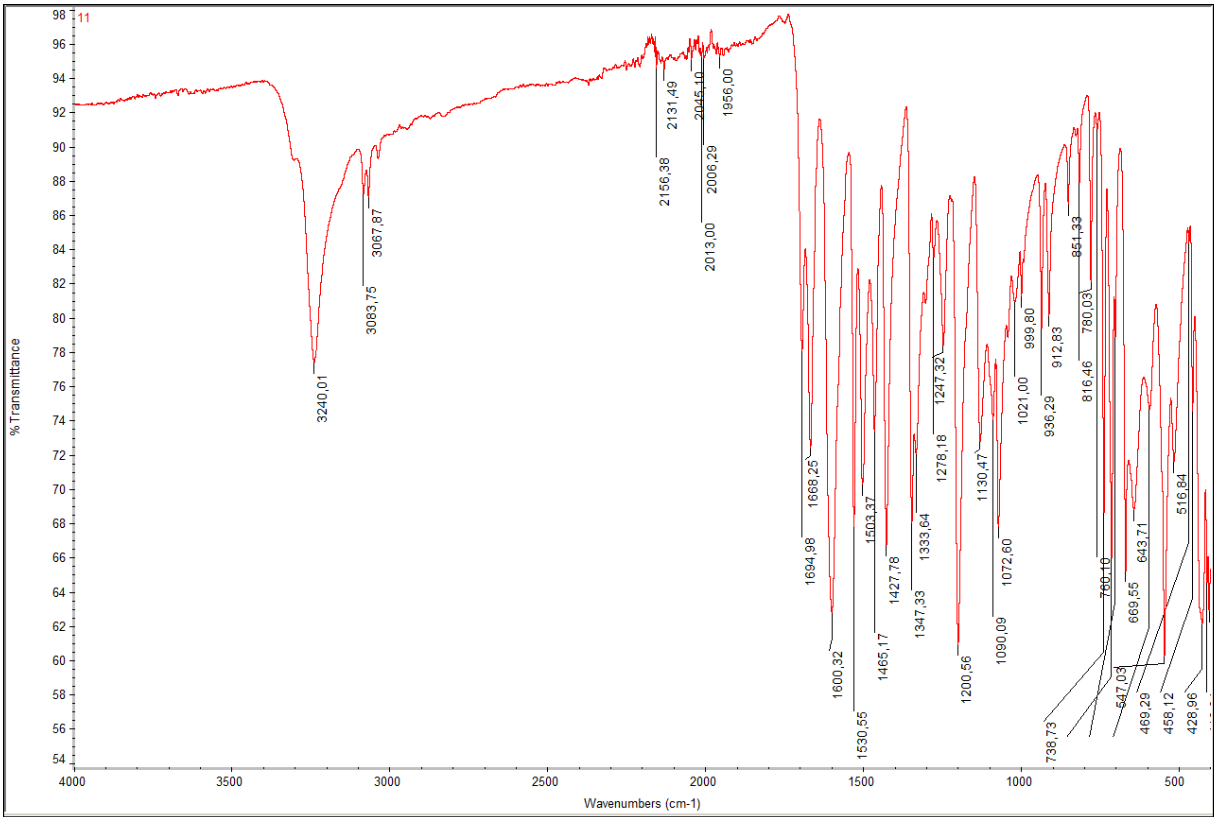

Figure S76. FT-IR spectrum of compound **32**

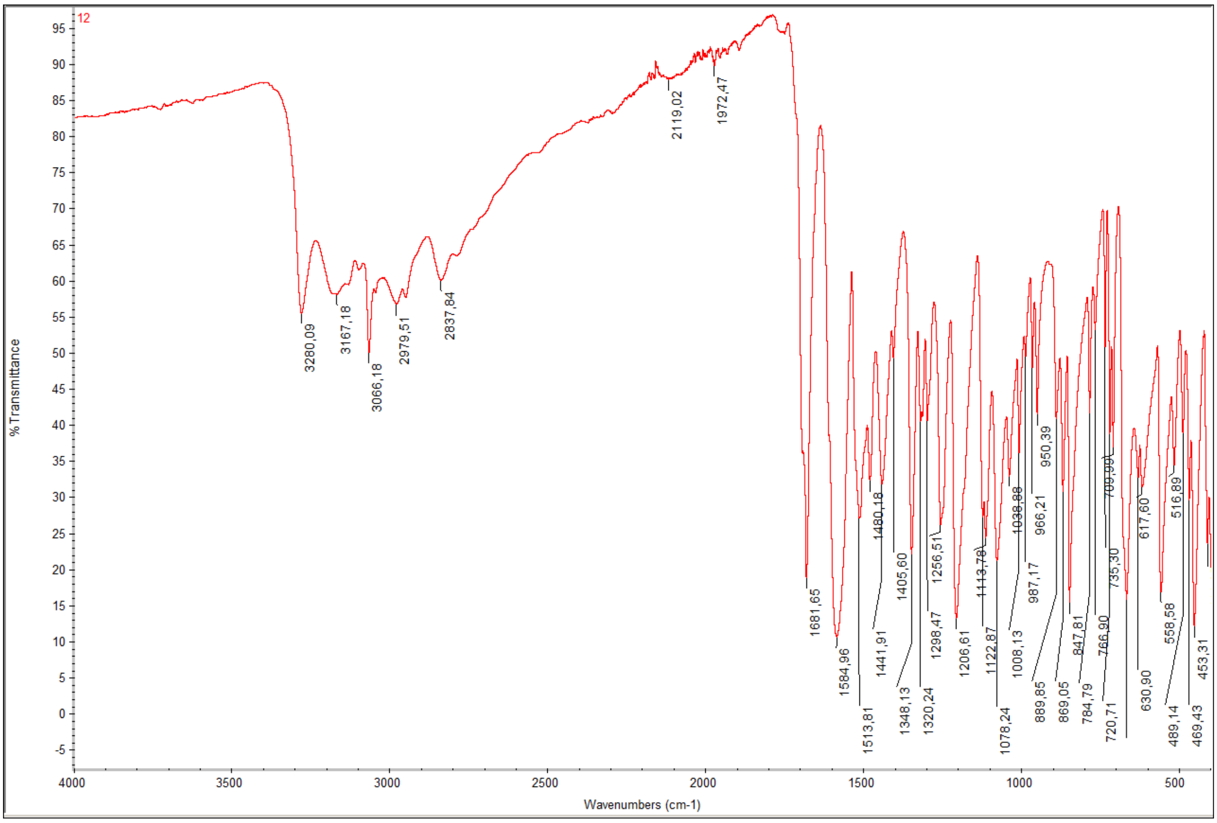

Figure S77. FT-IR spectrum of compound **33**

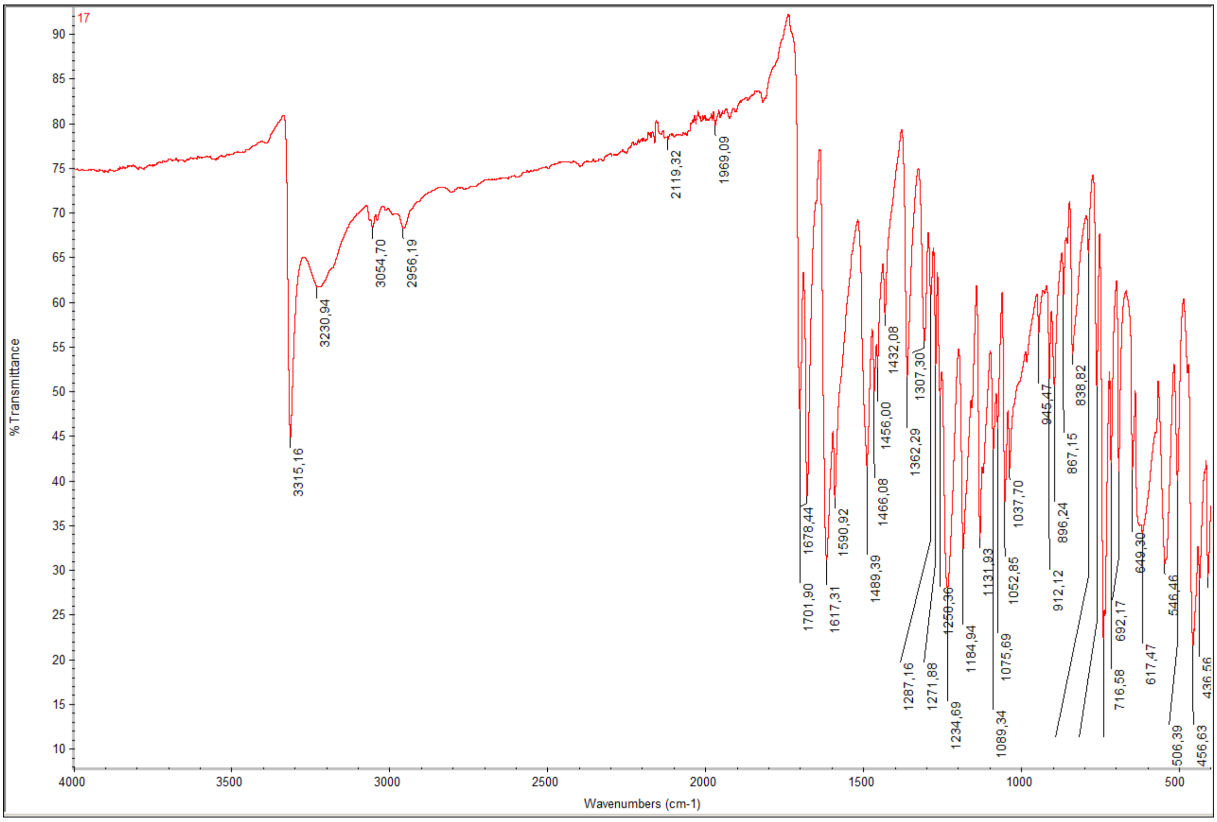

Figure S78. FT-IR spectrum of compound **34**

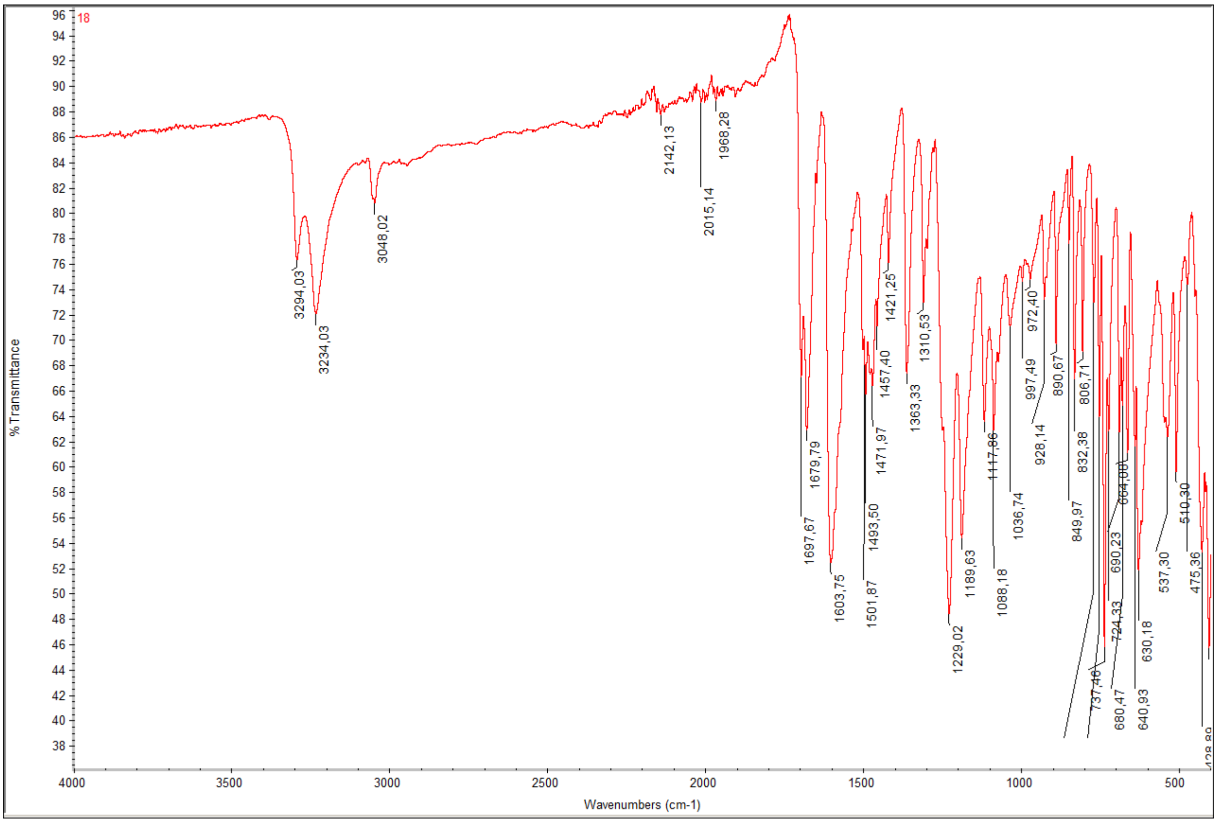

Figure S79. FT-IR spectrum of compound **35**

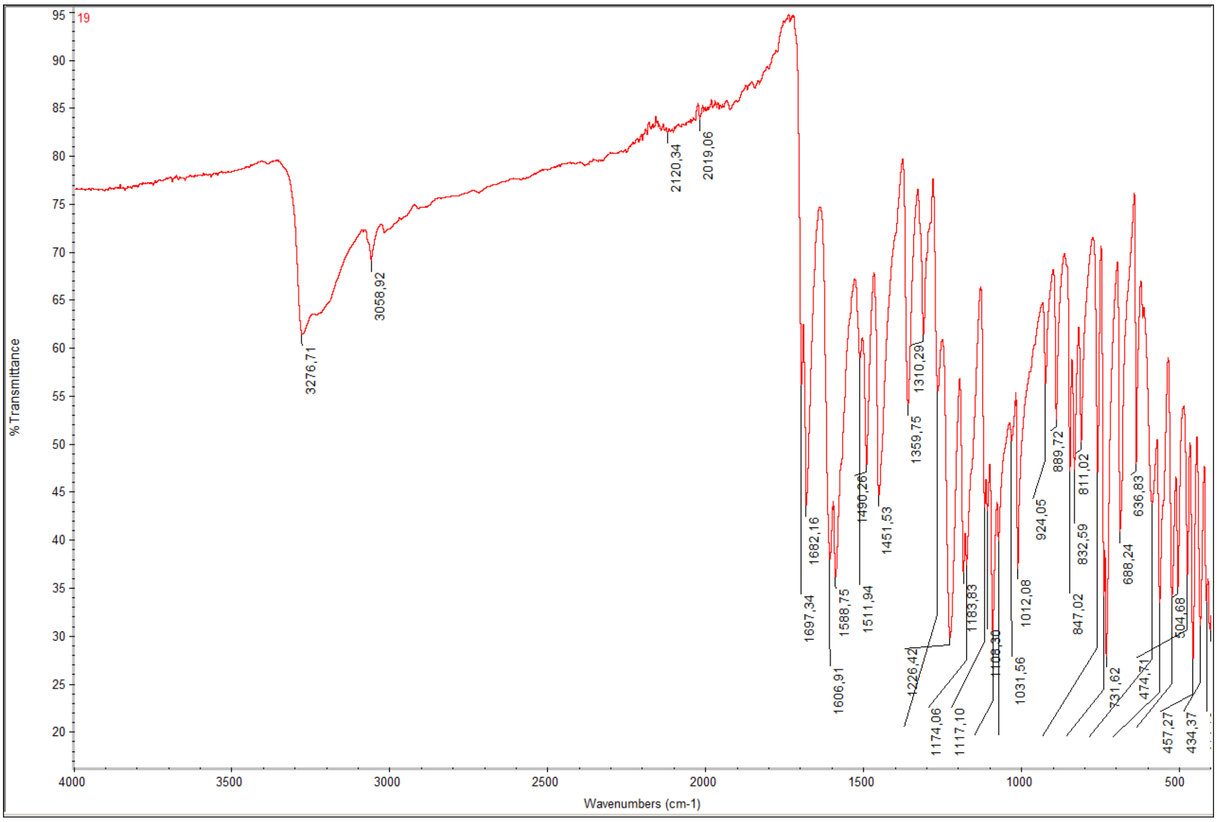

Figure S80. FT-IR spectrum of compound **36**

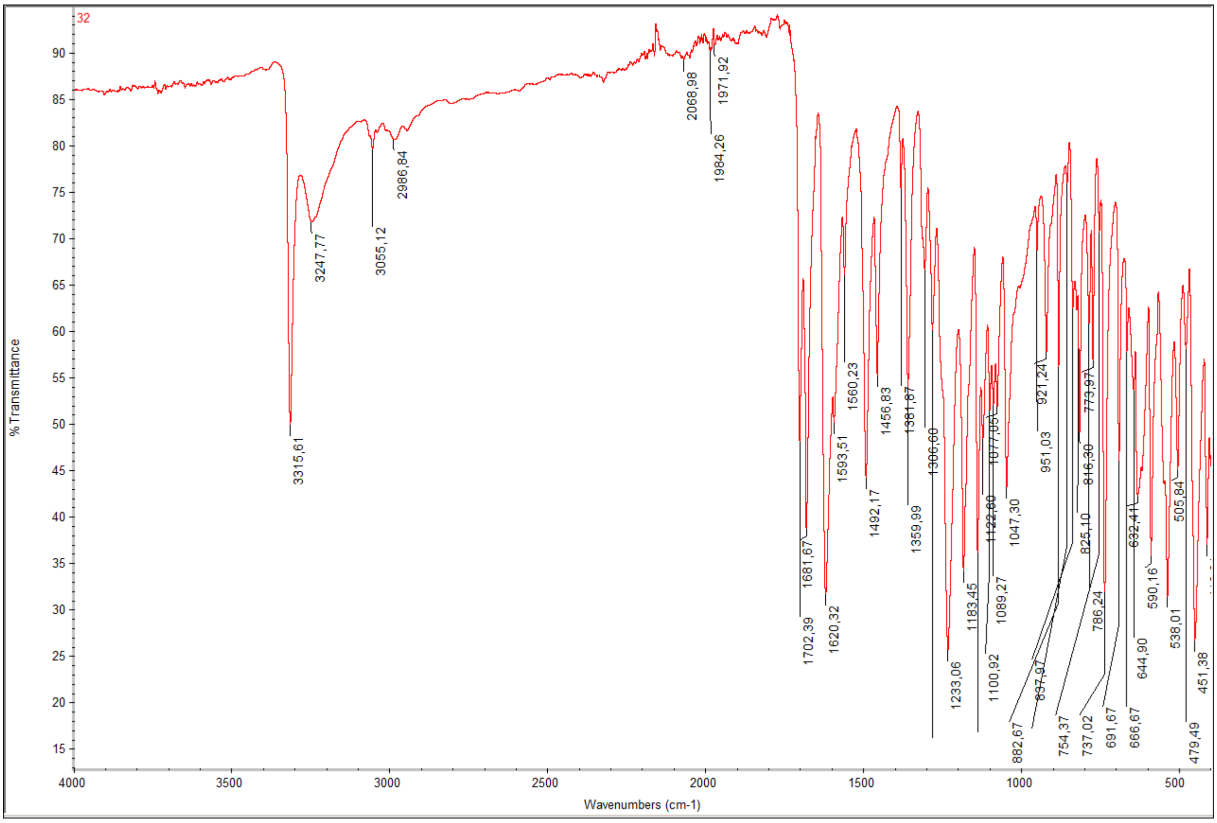

Figure S81. FT-IR spectrum of compound **37**

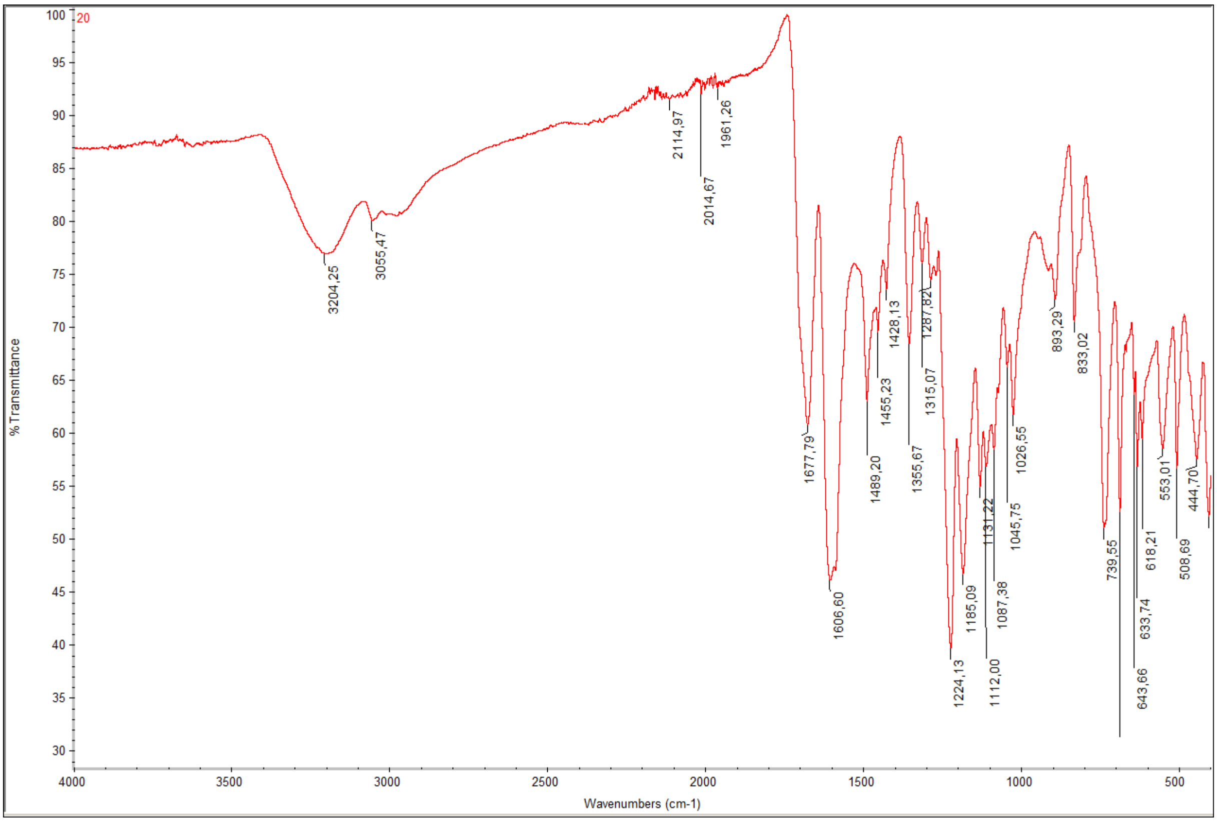

Figure S82. FT-IR spectrum of compound **38**

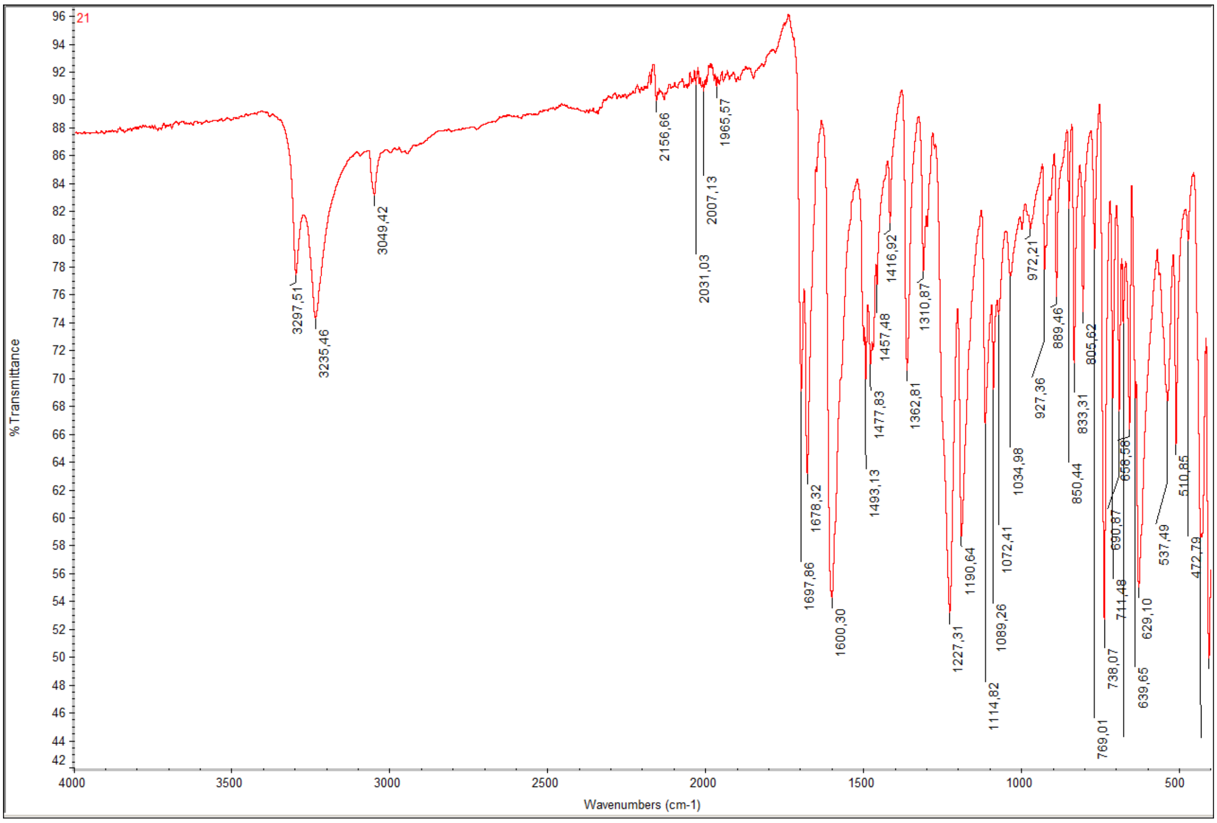

Figure S83. FT-IR spectrum of compound **39**

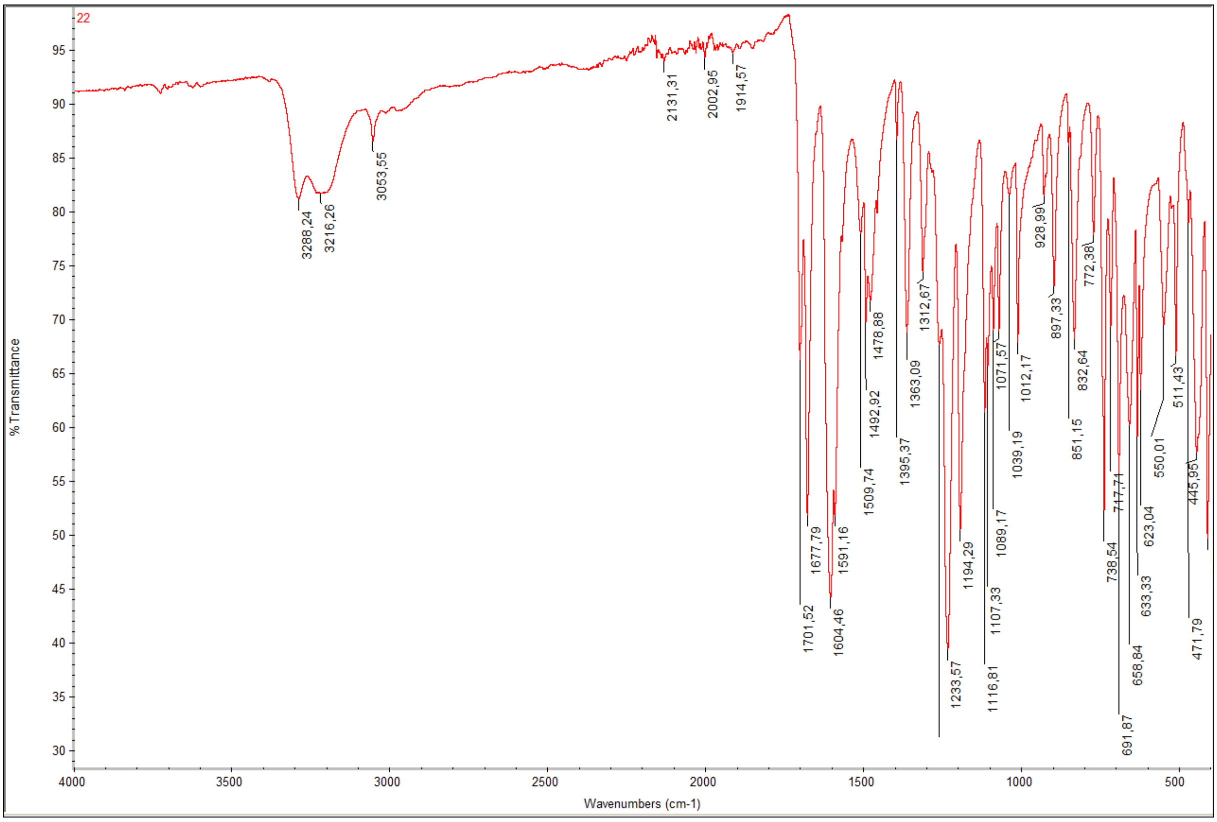

Figure S84. FT-IR spectrum of compound **40**

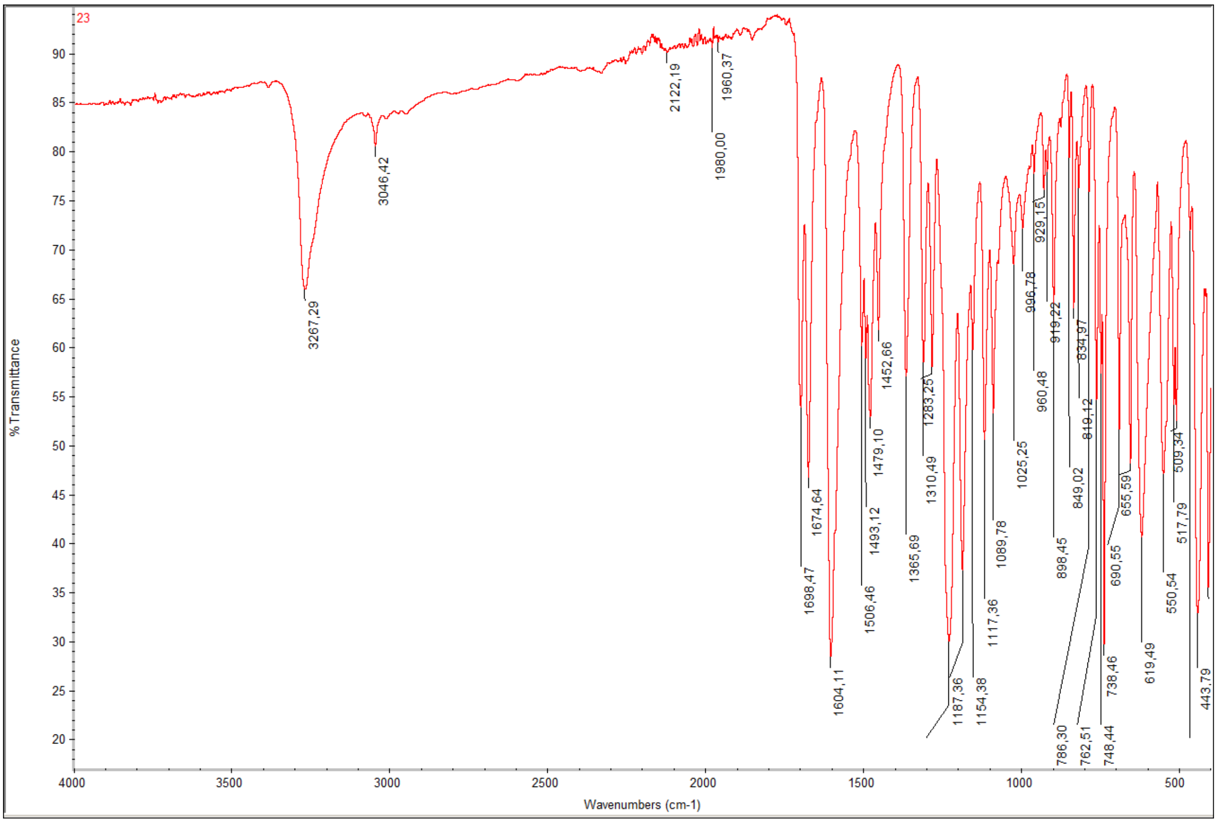

Figure S85. FT-IR spectrum of compound **41**

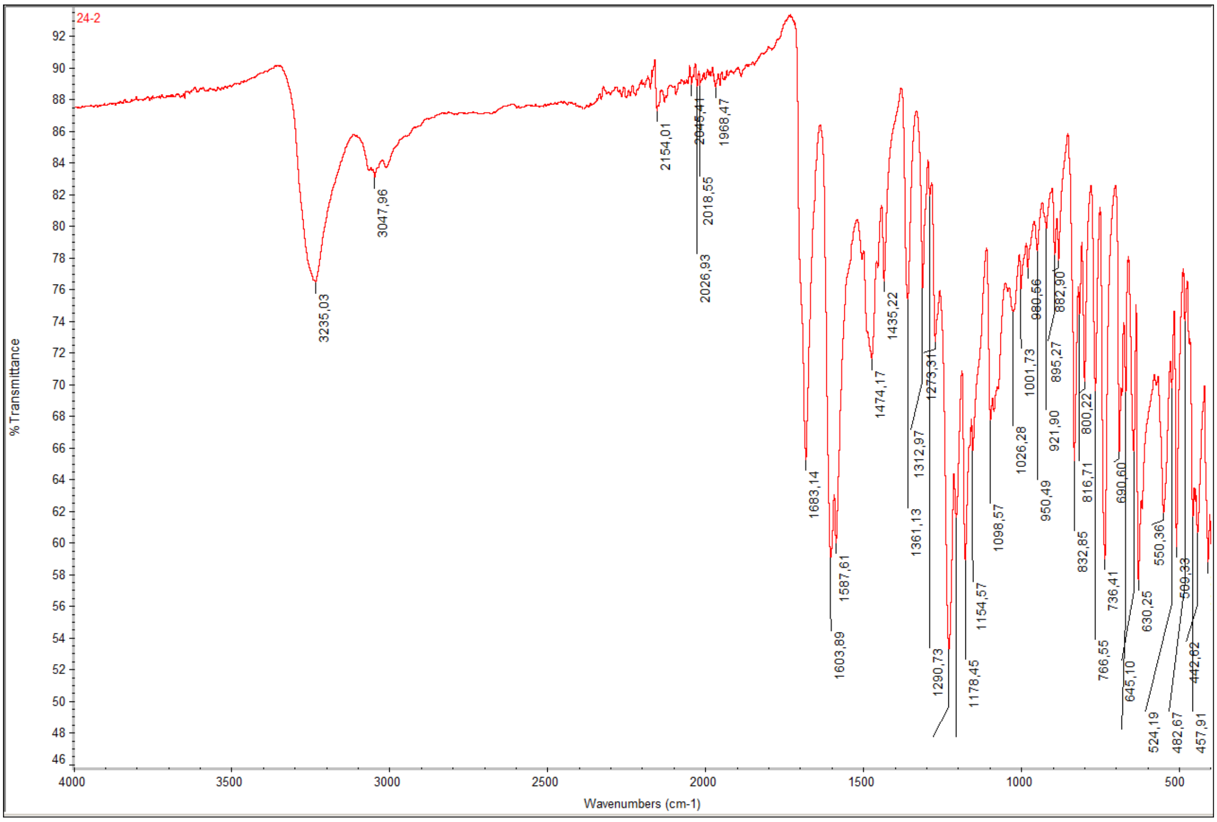

Figure S86. FT-IR spectrum of compound **42**

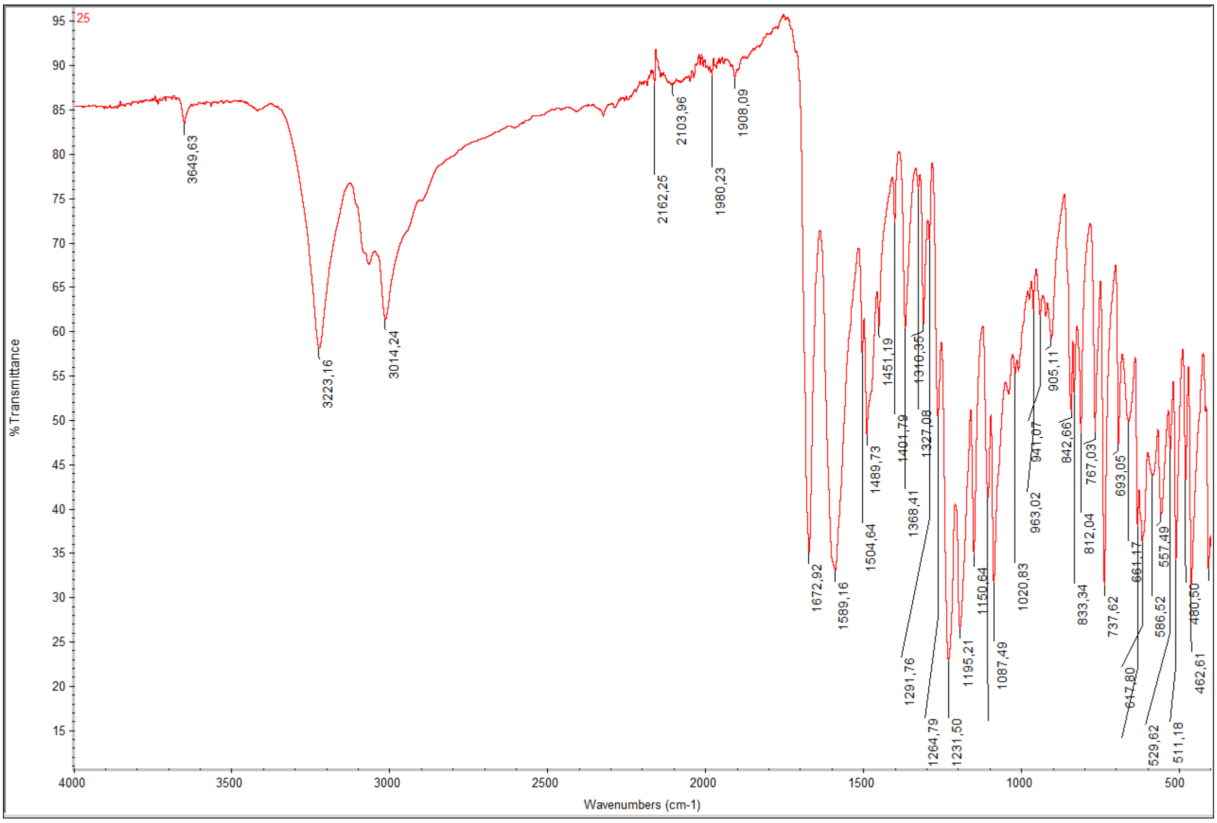

Figure S87. FT-IR spectrum of compound **43**

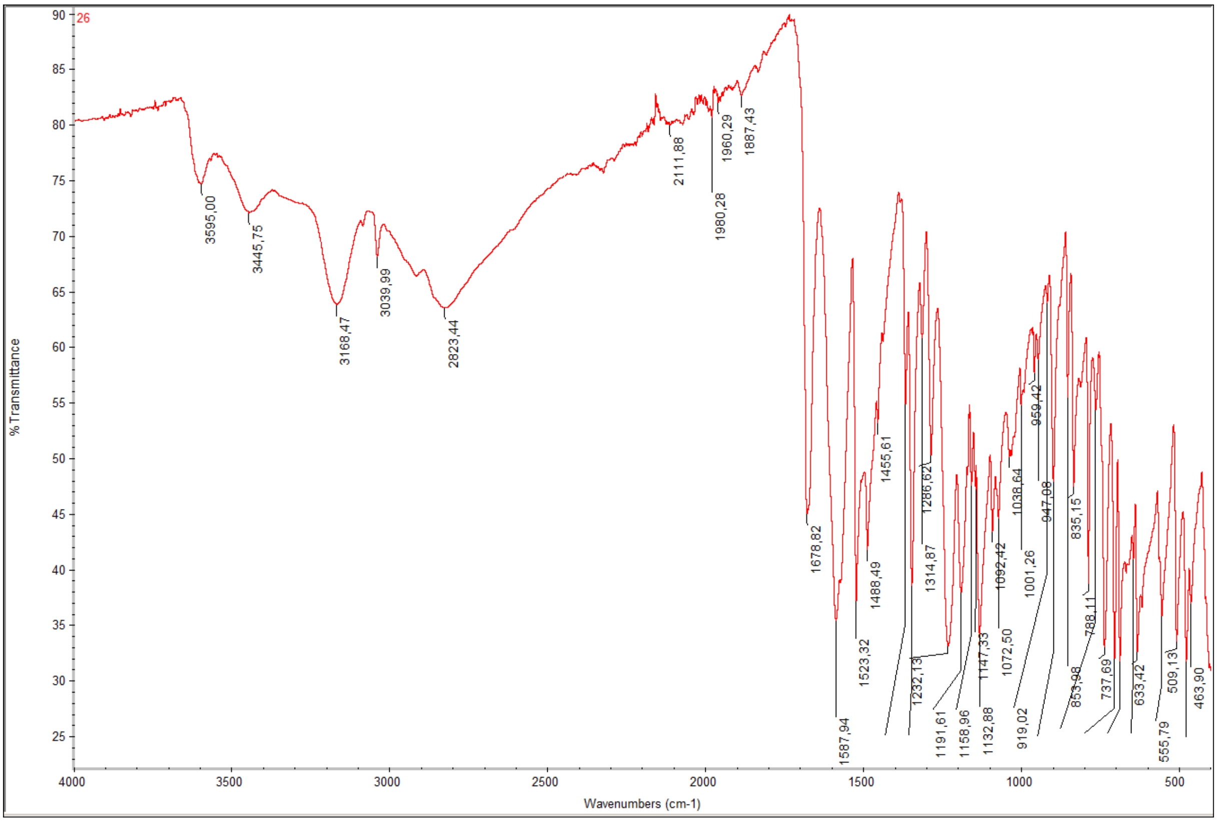

Figure S88. FT-IR spectrum of compound **44**

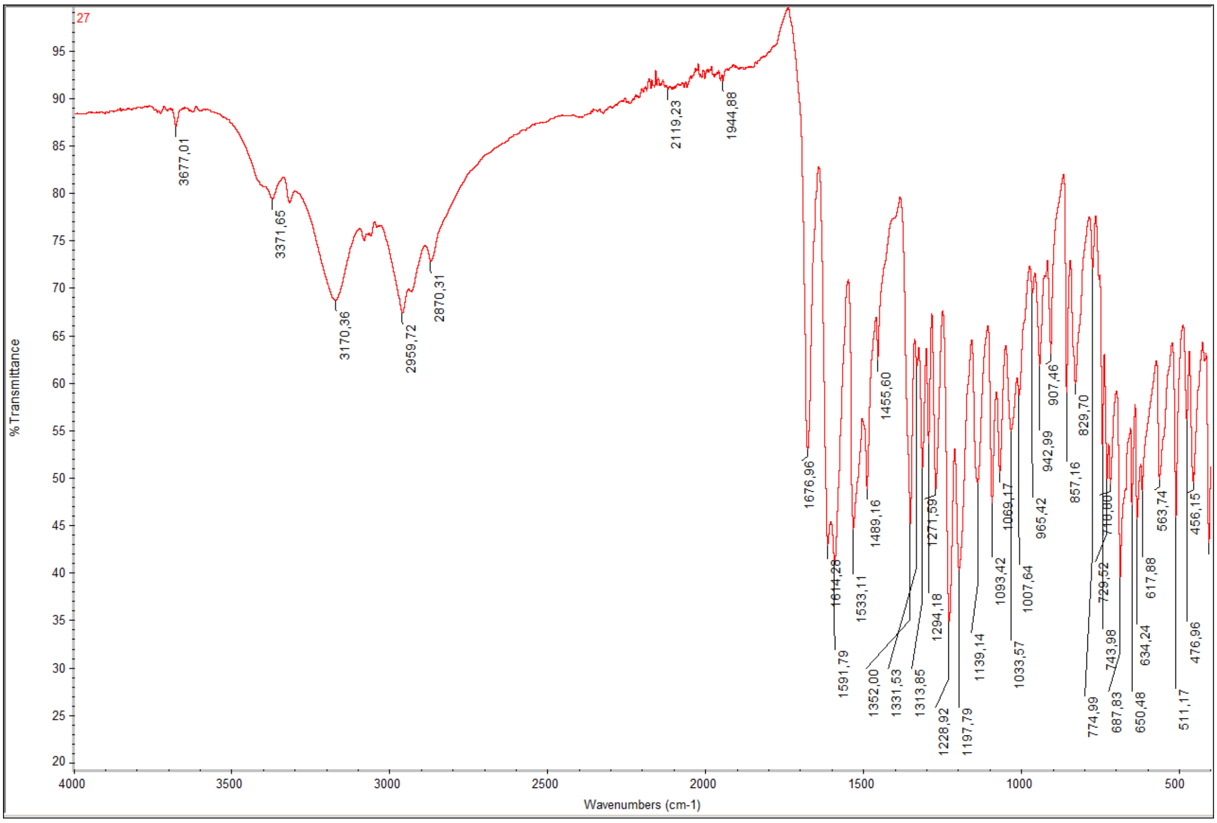

Figure S89. FT-IR spectrum of compound **45**

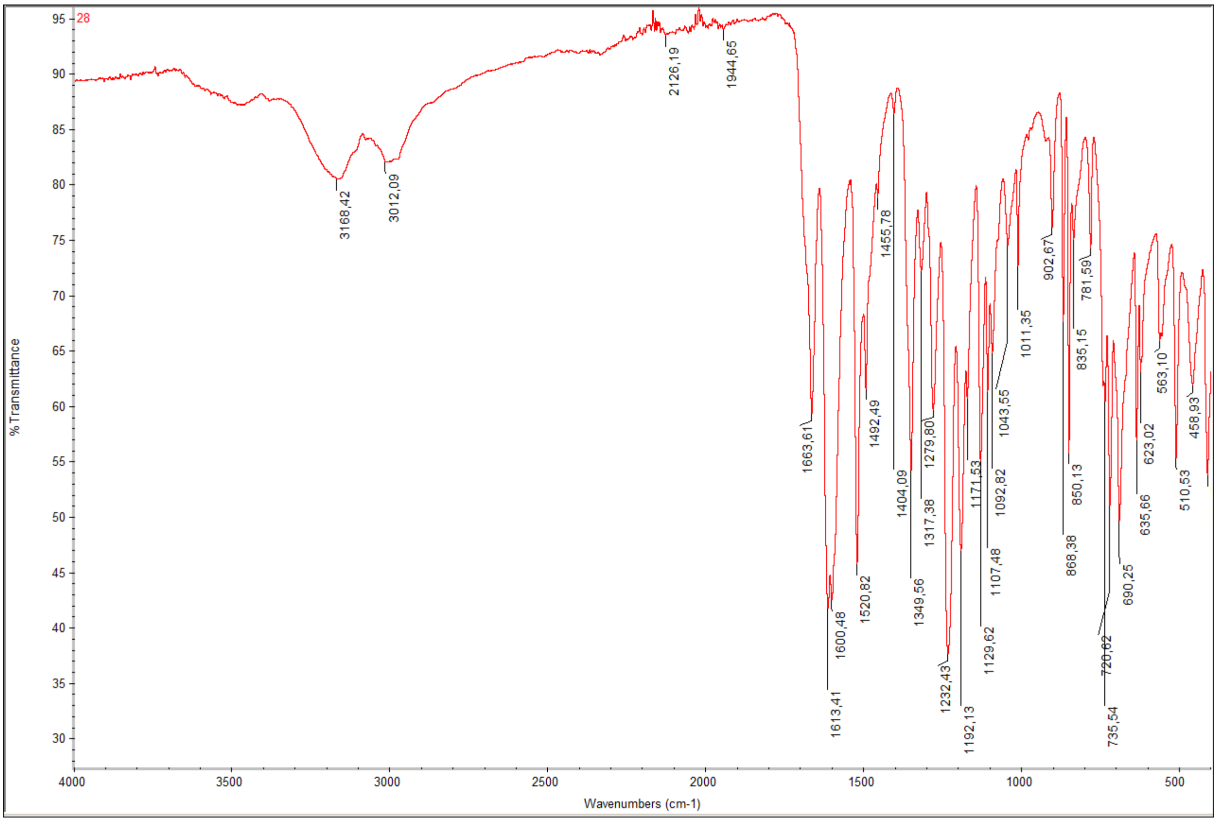

Figure S90. FT-IR spectrum of compound **46**

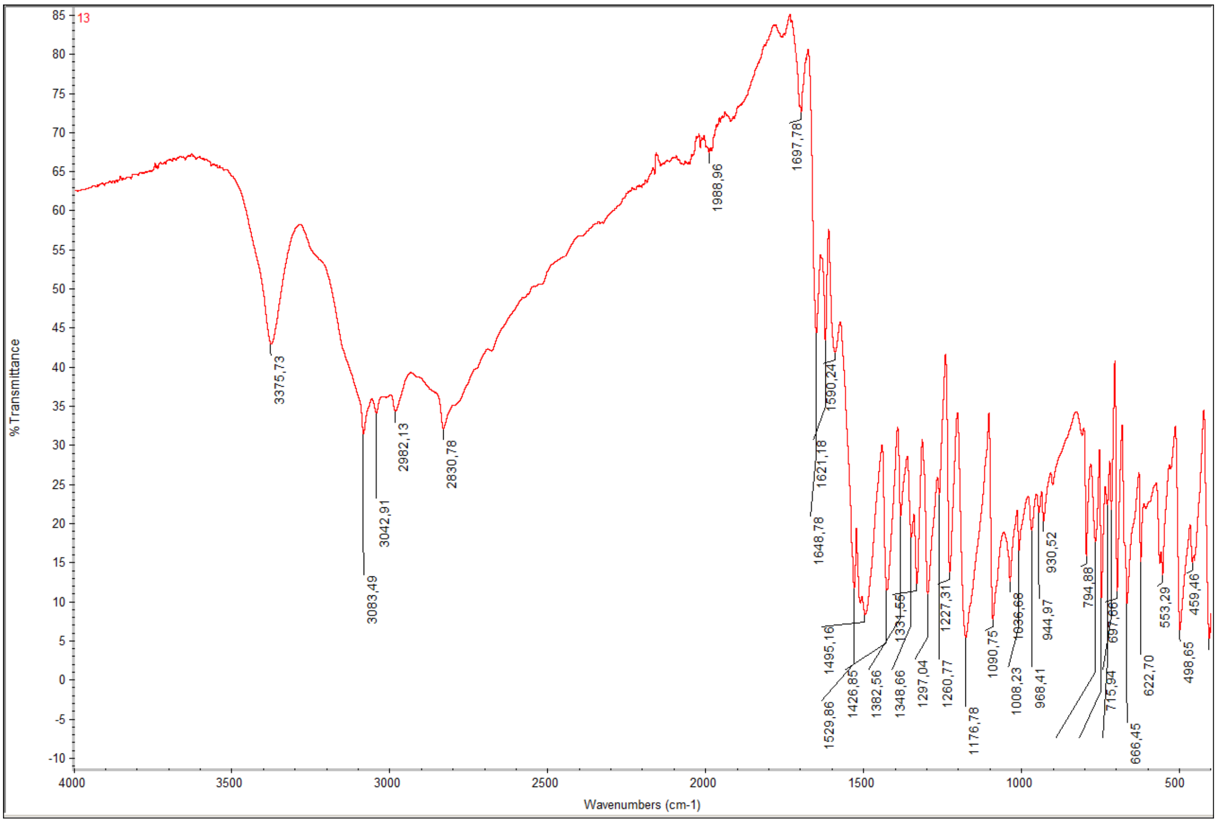

Figure S91. FT-IR spectrum of compound **47**

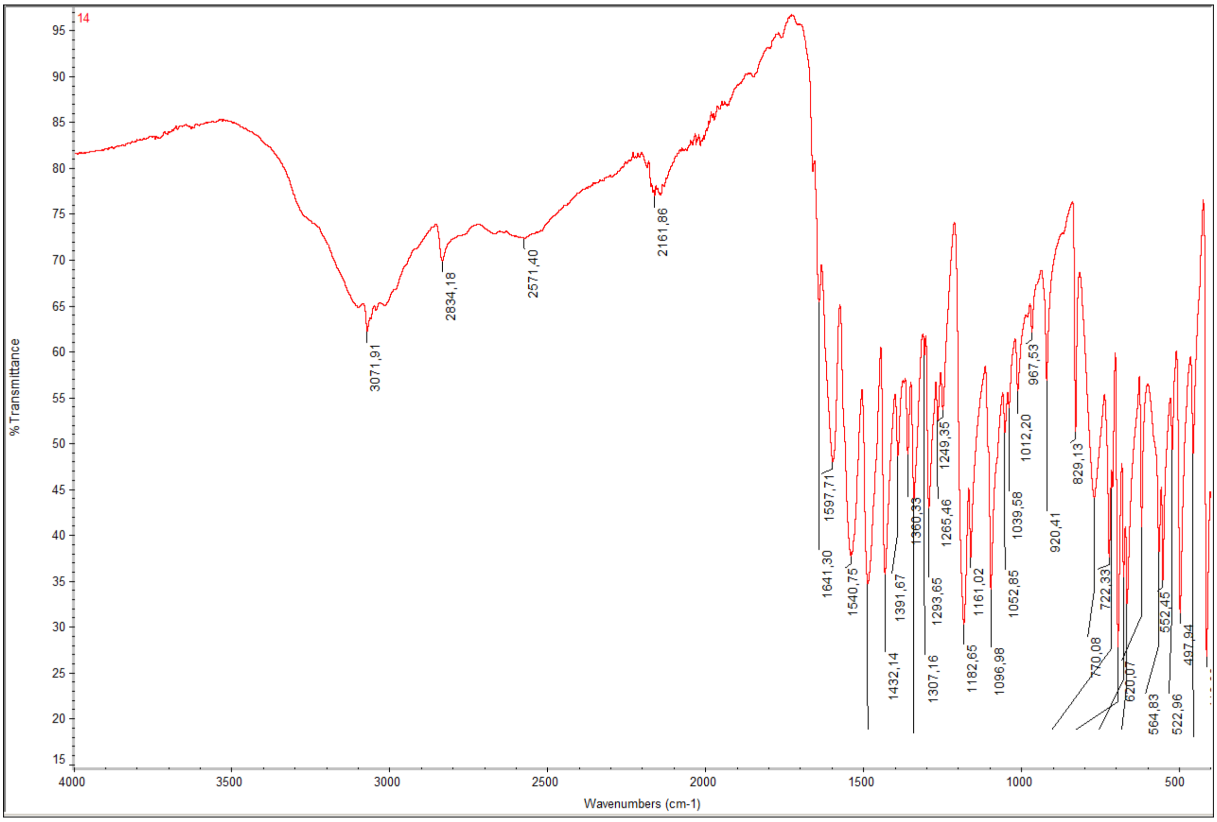

Figure S92. FT-IR spectrum of compound **48**

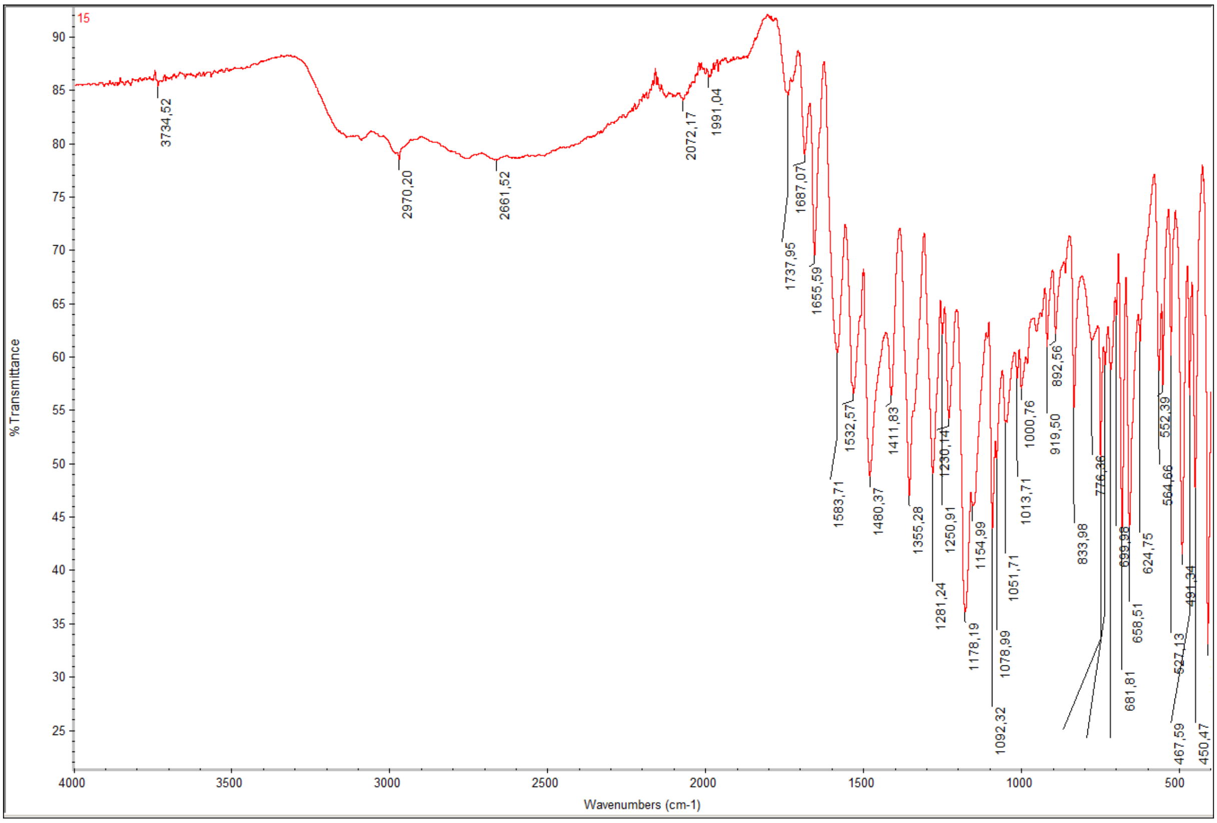

Figure S93. FT-IR spectrum of compound **49**

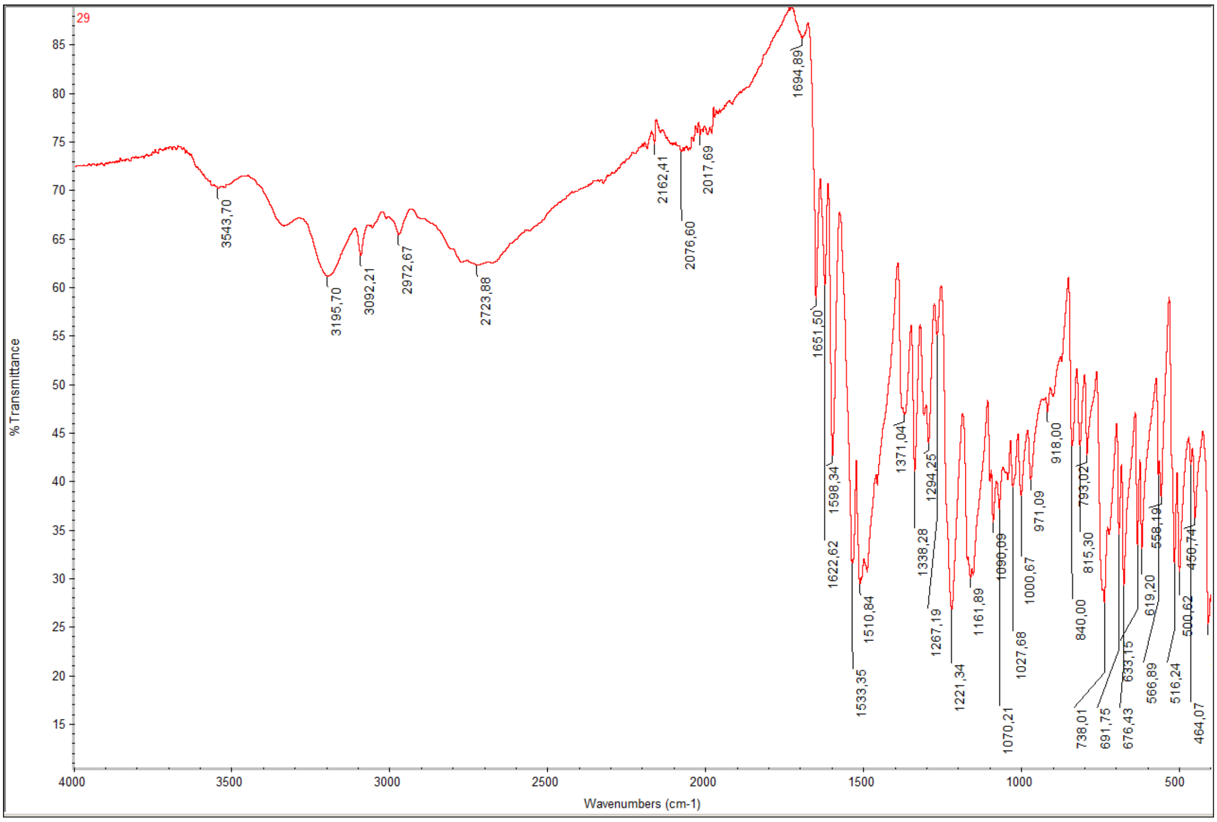

Figure S94. FT-IR spectrum of compound **50**

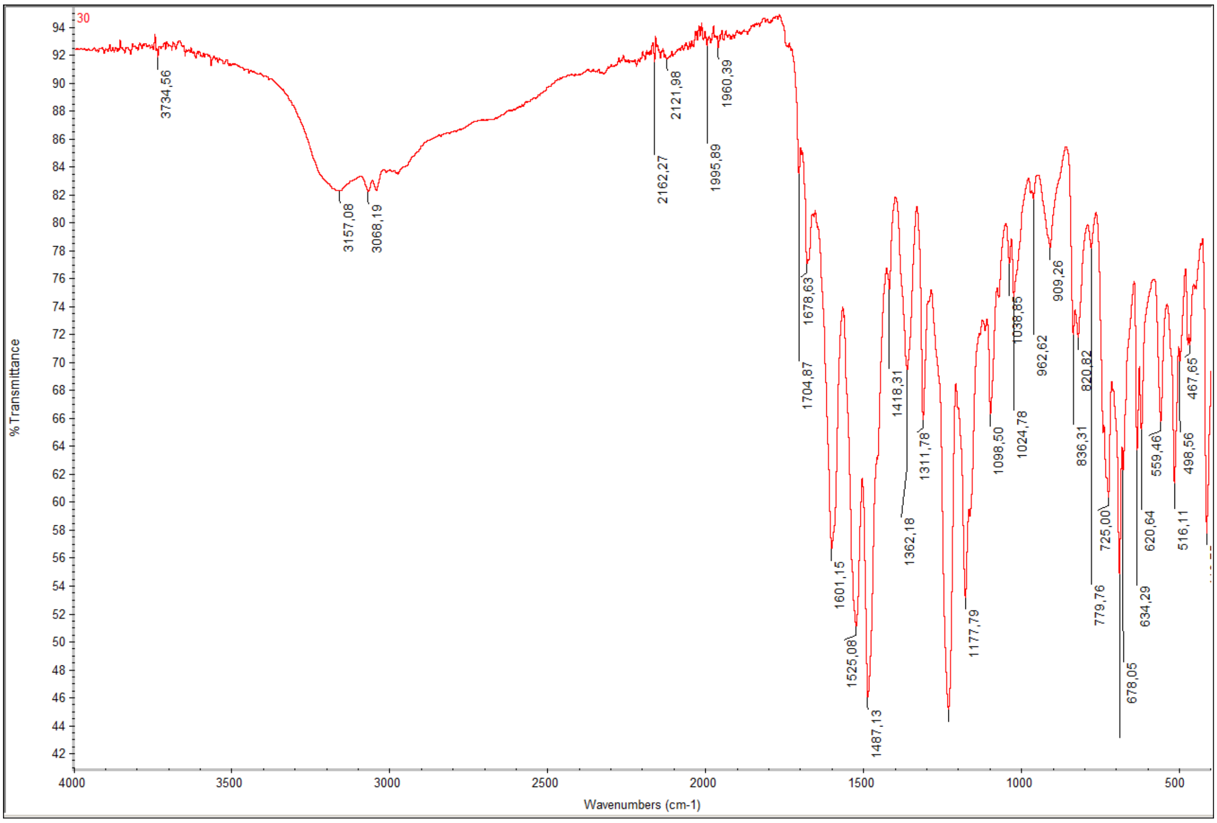

Figure S95. FT-IR spectrum of compound **51**

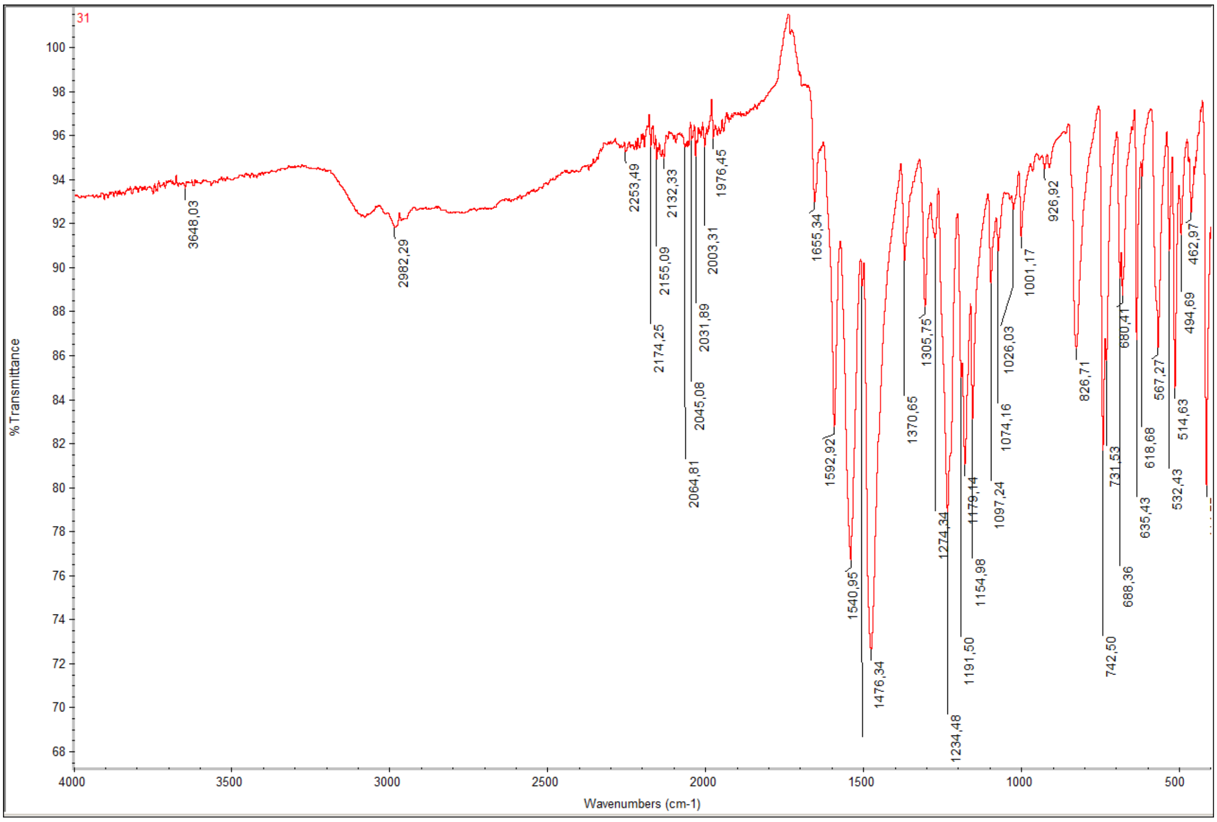

Figure S96. FT-IR spectrum of compound **52**

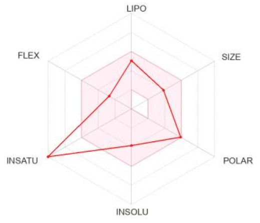

21

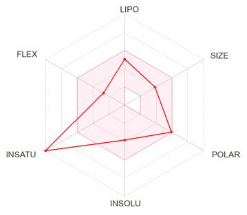

22

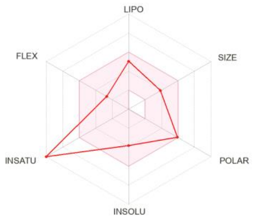

23

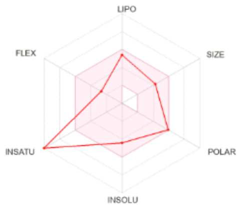

24

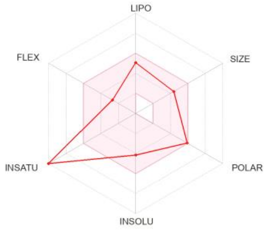

25

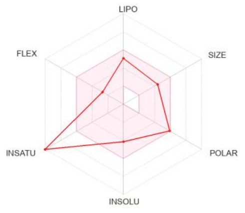

26

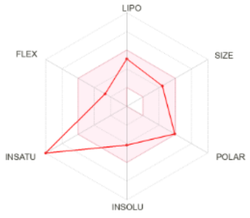

27

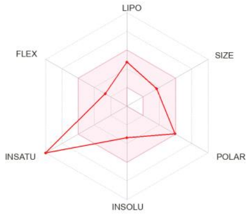

28

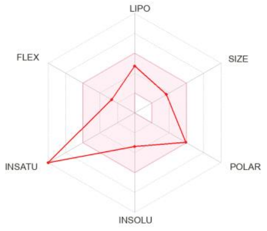

29

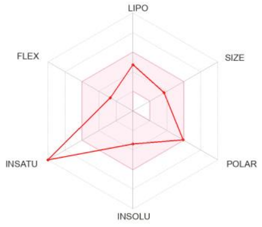

30

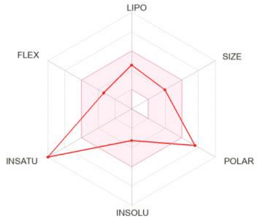

31

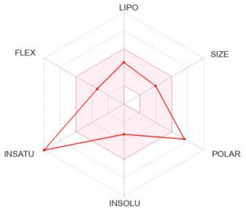

32

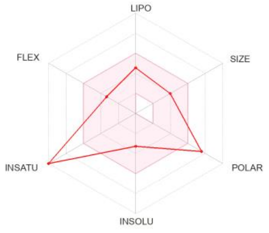

33

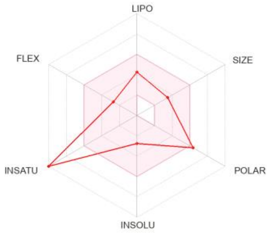

47

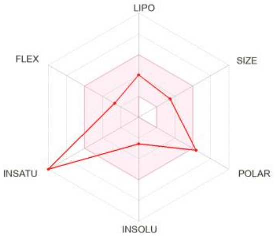

48

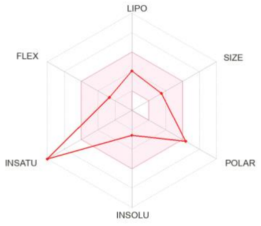

49

Figure S97. Bioavailability radar for 3-unsubstituted rhodanine–hydrazide derivatives (**21**–**33** and **47**–**49**). The pink area represents the optimal range for each of the properties (lipophilicity: XLOGP3 between −0.7 and +5.0, size: M between 150 and 500 g/mol, polarity: TPSA between 20 and 130 A2, solubility: log S not higher than six, saturation: fraction of carbons in the sp3 hybridization not less than 0.25, and flexibility: no more than nine rotatable bonds).

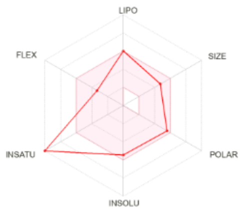

34

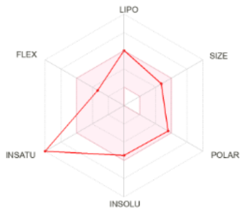

35

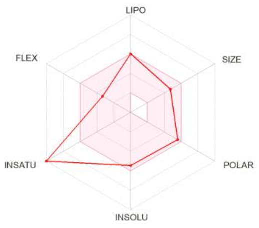

36

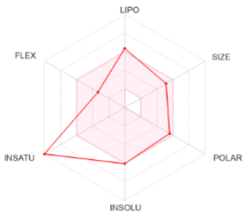

37

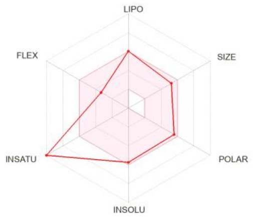

38

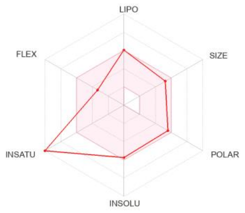

39

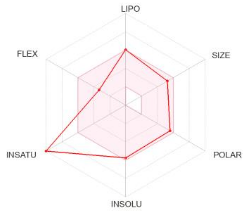

40

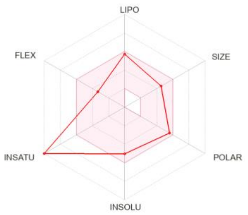

41

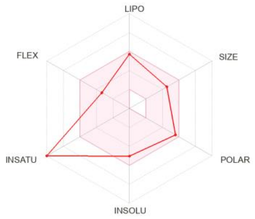

42

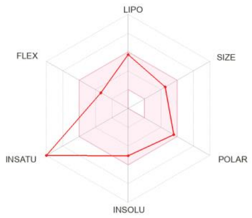

43

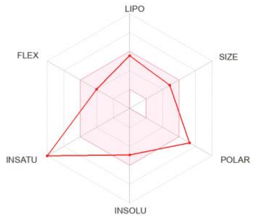

44

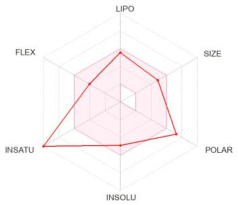

45

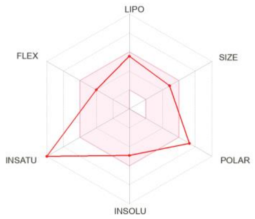

46

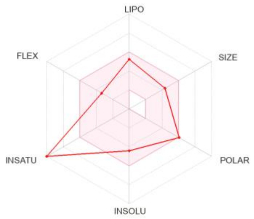

50

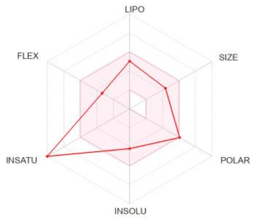

51

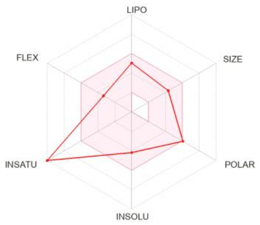

52

Figure S98. Bioavailability radar for 3-substituted rhodanine–hydrazide hybrids (**34**–**46** and **50**–**52**). The pink area represents the optimal range for each of the properties (lipophilicity: XLOGP3 between −0.7 and +5.0, size: M between 150 and 500 g/mol, polarity: TPSA between 20 and 130 A2, solubility: log S not higher than six, saturation: fraction of carbons in the sp3 hybridization not less than 0.25, and flexibility: no more than nine rotatable bonds).

Table S1. Physicochemical properties of compounds **21–52** obtained using the SwissADME server.

Compound	Lipinski Rule of Five				
	HBA[1]	HBD[2]	Molecular weight [g/mol]	Log P (MLOGP)	Violation
**21**	2	3	313.78	1.01	0
**22**	2	3	313.78	1.01	0
**23**	2	3	313.78	1.01	0
**24**	2	3	348.23	1.55	0
**25**	2	3	358.23	1.14	0
**26**	2	3	358.23	1.14	0
**27**	2	3	358.23	1.14	0
**28**	3	3	297.33	0.87	0
**29**	3	3	297.33	0.87	0
**30**	3	3	297.33	0.87	0
**31**	4	3	324.34	-0.47	0
**32**	4	3	324.34	-0.47	0
**33**	4	3	324.34	-0.47	0
**34**	2	2	389.88	2.57	0
**35**	2	2	389.88	2.57	0
**36**	2	2	389.88	2.57	0
**37**	2	2	424.32	3.07	0
**38**	2	2	434.33	2.68	0
**39**	2	2	434.33	2.68	0
**40**	2	2	434.33	2.68	0
**41**	3	2	373.42	2.45	0
**42**	3	2	373.42	2.45	0
**43**	3	2	373.42	2.45	0
**44**	4	2	400.43	1.14	0
**45**	4	2	400.43	1.14	0
**46**	4	2	400.43	1.14	0

**47**	3	3	280.33	-0.65	0
**48**	3	3	280.33	-0.65	0
**49**	3	3	280.33	-0.65	0
**50**	3	2	356.42	1.01	0
**51**	3	2	356.42	1.01	0
**52**	3	2	356.42	1.01	0

[1]HBA – hydrogen bond acceptors; [2]HBD – hydrogen bond donors.

Table S2. MIC and MBC for rhodanine-hydrazide hybrids (**21**–**52**) against Gram-positive bacteria.

Microorganism	Gram-positive bacteria					
	*S.a.**	*S.a.***	*S.e.*	*M.l.*	*B.c.*	*E.f.*
Compound	MIC/MBC (μg/mL)	MIC/MBC (μg/mL)	MIC/MBC (μg/mL)	MIC/MBC (μg/mL)	MIC/MBC (μg/mL)	MIC/MBC (μg/mL)
**21**	250/500	1000/1000	500/>1000	125/250	1000/>1000	1000/>1000
**22**	125/250	250/500	125/125	125/125	125/>1000	62.5/125
**23**	125/125	125/250	125/125	125/125	125/1000	62.5/125
**24**	125/250	250/500	250/250	125/250	250/>1000	62.5/125
**25**	250/1000	1000/1000	500/>1000	125/250	1000/>1000	500/>1000
**26**	125/125	125/125	125/125	125/125	125/>1000	62.5/125
**27**	125/250	250/250	125/250	125/250	250/>1000	125/250
**28**	250/500	>1000/>1000	>1000/>1000	125/250	>1000/>1000	>1000/>1000
**29**	250/500	500/1000	250/250	125/250	500/>1000	62.5/125
**30**	125/250	500/500	250/500	62.5/250	500/>1000	62.5/125
**31**	250/>1000	1000/>1000	1000/>1000	125/250	>1000/>1000	1000/>1000
**32**	125/125	250/500	125/250	125/250	250/>1000	62.5/250
**33**	125/125	500/500	125/>1000	125/125	250/>1000	62.5/125
**34**	500/1000	1000/1000	250/1000	125/500	500/>1000	500/>1000
**35**	125/500	62.5/500	125/250	62.5/125	125/>1000	62.5/250
**36**	125/1000	125/500	125/250	31.3/62.5	125/>1000	62.5/500
**37**	500/1000	500/1000	250/500	125/250	500/>1000	62.5/1000
**38**	500/1000	1000/1000	500/500	125/500	500/>1000	125/>1000
**39**	125/>1000	62.5/1000	125/250	62.5/125	125/>1000	62.5/250
**40**	125/>1000	125/1000	62.5/250	31.3/250	125/>1000	31.3/500
**41**	250/500	500/500	250/500	125/250	250/>1000	500/>1000
**42**	250/500	125/1000	250/500	125/250	250/>1000	500/1000
**43**	250/500	500/1000	250/500	62.5/500	250/>1000	125/500
**44**	500/>1000	500/1000	250/250	250/250	1000/>1000	500/>1000
**45**	250/1000	1000/1000	125/500	125/500	500/>1000	125/>1000
**46**	250/500	500/1000	31.3/125	62.5/250	500/>1000	62.5/500
**47**	>1000/>1000	>1000/>1000	>1000/>1000	250/250	250/>1000	250/>1000
**48**	>1000/ND	>1000/ND	>1000/ND	>1000/ND	>1000/ND	>1000/ND
**49**	>1000/ND	>1000/ND	>1000/ND	>1000/ND	>1000/ND	>1000/ND
**50**	125/>1000	125/>1000	62.5/1000	31.3/31.3	125/1000	125/>1000
**51**	125/1000	1000/1000	500/1000	250/500	500/>1000	250/1000
**52**	250/1000	1000/1000	1000/1000	250/1000	500/>1000	250/500
VAN	0.98/0.98	0.98/0.98	0.98/0.98	0.12/0.12	1.95/3.9	0.98/15.6

*S.a.** – *Staphylococcus aureus* ATCC 25923; *S.a.*** – *Staphylococcus aureus* ATCC BAA1707; *S.e.* – *Staphylococcus epidermidis* ATCC 12228; *M.l.* – *Micrococcus luteus* ATCC 10240; *B.c.* – *Bacillus cereus* ATCC 10876; *E.f.* – *Enterococcus faecalis* ATCC 29212; VAN – Vancomycin.

Table S3. MIC and MBC or MFC for rhodanine-hydrazide hybrids (**21**–**52**) against Gram-negative bacteria and yeasts strains.

MO	Gram-negative bacteria					Yeasts		
	S.t.	*E.c.*	*P.m.*	*K.p.*	*P.a.*	*C.g.*	*C.a.*	*C.p.*
Cpd	MIC/MBC (μg/mL)	MIC/MBC (μg/mL)	MIC/MBC (μg/mL)	MIC/MBC (μg/mL)	MIC/MBC (μg/mL)	MIC/MFC (μg/mL)	MIC/MFC (μg/mL)	MIC/MFC (μg/mL)
**21**	>1000/ND	>1000/ND	>1000/ND	>1000/ND	>1000/ND	>1000/ >1000	500/1000	250/500
**22**	>1000/ND	>1000/ND	1000/1000	>1000/ND	>1000/ND	>1000/ >1000	1000/ >1000	500/500
**23**	>1000/ND	>1000/ND	1000/1000	>1000/ND	>1000/ND	1000/ >1000	500/>1000	125/500
**24**	>1000/ND	>1000/ND	>1000/ND	>1000/ND	>1000/ND	1000/ >1000	500/1000	125/500
**25**	>1000/ND	>1000/ND	>1000/ND	>1000/ND	>1000/ND	1000/ >1000	500/1000	125/500
**26**	>1000/ND	>1000/ND	1000/1000	>1000/ND	>1000/ND	>1000/ >1000	1000/ >1000	250/500
**27**	>1000/ND	>1000/ND	>1000/ND	>1000/ND	>1000/ND	>1000/ >1000	1000/ >1000	31.3/1000
**28**	>1000/ND	>1000/ND	>1000/ND	>1000/ND	>1000/ND	>1000/ >1000	1000/ >1000	250/500
**29**	>1000/ND	>1000/ND	>1000/ND	>1000/ND	>1000/ND	>1000/ >1000	500/>1000	500/500
**30**	>1000/ND	>1000/ND	>1000/ND	>1000/ND	>1000/ND	1000/1000	500/1000	250/250
**31**	>1000/ND	>1000/ND	>1000/ND	>1000/ND	>1000/ND	>1000/ >1000	500/>1000	250/500
**32**	>1000/ND	>1000/ND	>1000/ND	>1000/ND	>1000/ND	1000/1000	250/1000	31.3/500
**33**	>1000/ND	>1000/ND	>1000/ND	>1000/ND	>1000/ND	500/1000	125/1000	31.3/500
**34**	>1000/ND	>1000/ND	>1000/ND	>1000/ND	>1000/ND	125/250	125/500	15.6/500
**35**	>1000/ND	>1000/ND	>1000/ND	>1000/ND	>1000/ND	1000/ >1000	250/>1000	62.5/>1000
**36**	>1000/ND	>1000/ND	>1000/ND	>1000/ND	>1000/ND	500/>1000	250/1000	125/1000
**37**	>1000/ND	>1000/ND	>1000/ND	>1000/ND	>1000/ND	250/>1000	125/500	31.3/500
**38**	>1000/ND	>1000/ND	1000/ >1000	1000/ >1000	>1000/ND	250/500	125/500	31.3/500
**39**	>1000/ND	>1000/ND	>1000/ND	>1000/ND	>1000/ND	1000/ >1000	250/1000	125/1000
**40**	>1000/ND	>1000/ND	>1000/ND	>1000/ND	>1000/ND	1000/ >1000	250/1000	125/1000
**41**	>1000/ND	>1000/ND	>1000/ND	>1000/ND	>1000/ND	250/250	125/250	125/500
**42**	>1000/ND	>1000/ND	>1000/ND	>1000/ND	>1000/ND	500/1000	250/1000	125/1000
**43**	>1000/ND	>1000/ND	>1000/ND	>1000/ND	>1000/ND	250/>1000	250/500	31.3/1000
**44**	500/>1000	>1000/ >1000	1000/ >1000	>1000/ >1000	>1000/ >1000	250/500	62.5/500	15.6/500
**45**	>1000/ND	>1000/ND	>1000/ND	>1000/ND	>1000/ND	1000/ >1000	500/>1000	31.3/>1000
**46**	>1000/ND	>1000/ND	>1000/ND	>1000/ND	>1000/ND	1000/ >1000	500/>1000	250/>1000
**47**	>1000/ND	>1000/ND	>1000/ND	>1000/ND	>1000/ND	1000/1000	1000/1000	1000/1000
**48**	>1000/ND	>1000/ND	>1000/ND	>1000/ND	>1000/ND	>1000/	1000/	500/>1000

						>1000	>1000	
**49**	>1000/ND	>1000/ND	>1000/ND	>1000/ND	>1000/ND	1000/ND	500/>1000	1000/ >1000
**50**	500/>1000	500/>1000	500/>1000	1000/ >1000	>1000/ >1000	250/>1000	250/>1000	31.3/1000
**51**	>1000/ND	>1000/ND	>1000/ND	>1000/ND	>1000/ND	500/1000	125/1000	62.5/500
**52**	>1000/ND	>1000/ND	>1000/ND	>1000/ND	>1000/ND	250/500	125/1000	31.3/500
CIP	0.061/0.061	0.015/0.08	0.03/0.03	0.122/0.24	0.488/0.98	-	-	-
NYS	-	-	-	-	-	0.24/0.48	0.48/0.48	0.24/0.48

MO – microorganism; Cpd – compound; *S.t.* – *Salmonella typhimurium* ATCC 14028; *E.c.* – *Escherichia coli* ATCC 25922; *P.m.* – *Proteus mirabilis* ATCC 12453; *K.p.* – *Klebsiella pneumoniae* ATCC 13883; *P.a.* – *Pseudomonas aeruginosa* ATCC 9027; *C.g.* – *Candida glabrata* ATCC 66032; *C.a.* – *Candida albicans* ATCC 102231; *C.p.* – *Candida parapsilosis* ATCC 22019; CIP – Ciprofloxacin; NYS – Nystatin.
